# Indacenodibenzothiophenes: synthesis, optoelectronic properties and materials applications of molecules with strong antiaromatic character†Electronic supplementary information (ESI) available: Experimental details, spectroscopic data, computational details, device details and copies of ^1^H and ^13^C NMR spectra. CCDC 1451107–1451110. For ESI and crystallographic data in CIF or other electronic format see DOI: 10.1039/c6sc00950f


**DOI:** 10.1039/c6sc00950f

**Published:** 2016-05-13

**Authors:** Jonathan L. Marshall, Kazuyuki Uchida, Conerd K. Frederickson, Christian Schütt, Andrew M. Zeidell, Katelyn P. Goetz, Tristan W. Finn, Karol Jarolimek, Lev N. Zakharov, Chad Risko, Rainer Herges, Oana D. Jurchescu, Michael M. Haley

**Affiliations:** a Department of Chemistry & Biochemistry and Materials Science Institute , University of Oregon , Eugene , Oregon 97403-1253 , USA . Email: haley@uoregon.edu; b Department of Chemistry , Graduate School of Science , Osaka University , Toyonaka , Osaka 560-0043 , Japan; c Otto-Diels-Institute of Organic Chemistry , University of Kiel , Otto-Hahn-Platz 4 , Kiel 24098 , Germany; d Department of Physics , Wake Forest University , Winston-Salem , North Carolina 27109 , USA; e Department of Chemistry and Center for Applied Energy Research , University of Kentucky , Lexington , Kentucky 40506 , USA; f CAMCOR , University of Oregon , Eugene , Oregon 97403-1433 , USA

## Abstract

Indeno[1,2-*b*]fluorenes (IFs), while containing 4n π-electrons, are best described as two aromatic benzene rings fused to a weakly paratropic *s*-indacene core. In this study, we find that replacement of the outer benzene rings of an IF with benzothiophenes allows the antiaromaticity of the central *s*-indacene to strongly reassert itself. Herein we report a combined synthetic, computational, structural, and materials study of *anti*- and *syn*-indacenodibenzothiophenes (IDBTs). We have developed an efficient and scalable synthesis for preparation of a series of aryl- and ethynyl-substituted IDBTs. NICS-XY scans and ACID calculations reveal an increasingly antiaromatic core from [1,2-*b*]IF to *anti*-IDBT, with *syn*-IDBT being nearly as antiaromatic as the parent *s*-indacene. As an initial evaluation, the intermolecular electronic couplings and electronic band structure of a diethynyl *anti*-IDBT derivative reveal the potential for hole and/or electron transport. OFETs constructed using this molecule show the highest hole mobilities yet achieved for a fully conjugated IF derivative.

## Abstract 2

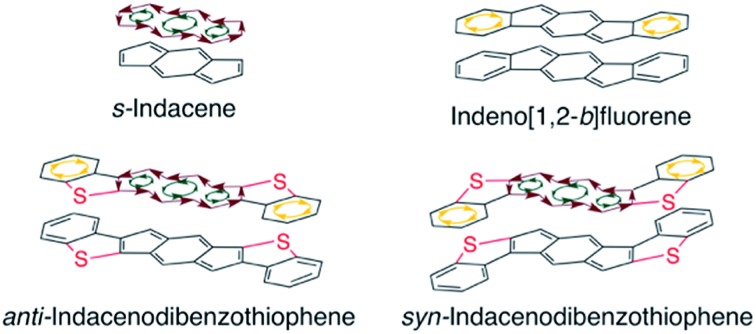
Exchanging fused benzenes for benzothiophenes results in pronounced antiaromaticity in the indacene core.

## Introduction

First predicted by Hückel in 1931^1^ and later proposed by Breslow in 1967,^2^ antiaromaticity describes conjugated cyclic systems containing 4n π electrons that are destabilized in comparison to a suitable reference compound.^3^ Many groups have sought to further expand this concept,3a,c,d,^4^ and the criteria for aromatic and antiaromatic compounds have been succinctly defined by Breslow,3*b* Krygowski,3*e* and Wiberg.4*i* In addition to containing 4n π-electron cyclic conjugation, antiaromatic compounds have decreased delocalization, smaller HOMO–LUMO energy gaps, and increased bond length alternation in comparison to aromatic molecules.3b,e,4i Antiaromatic compounds also exhibit paratropic ^1^H NMR chemical shifts and large, positive nucleus independent chemical shift (NICS) values.3b,4b,d,^5^ Although experimental examples of antiaromatic compounds are quite rare, they nonetheless have attracted the attention of chemists due to theoretical interest and potential materials science applications.4e,5b,^6^

Over the last 20 years, chemists have focused on the rational design of organic electronic materials.^7^ The synthesis and rigorous optical, electronic, and solid-state characterization of small molecule organic semiconductors has enhanced enormously our understanding of the fundamental principles necessary to design materials for high performing organic light emitting diodes (OLEDs), organic field effect transistors (OFETs), organic photovoltaics (OPVs), or other organic electronic devices.7e–k,^8^ Highly conjugated polycyclic hydrocarbons (CPHs) are a fascinating class of compounds often utilized in organic electronics due to their desirable photophysical and electronic properties.7a–d,7h–k,^9^ The study of these CPHs has resulted not only in functional materials, but also provided insight into fundamental chemical principles, such as the nature of the carbon–carbon bond, singlet-biradical character, and aromaticity and antiaromaticity, knowledge that in turn can be applied to designing better performing materials.7a–d,h–k,9a,e,f,^10^

**Fig. 1:**
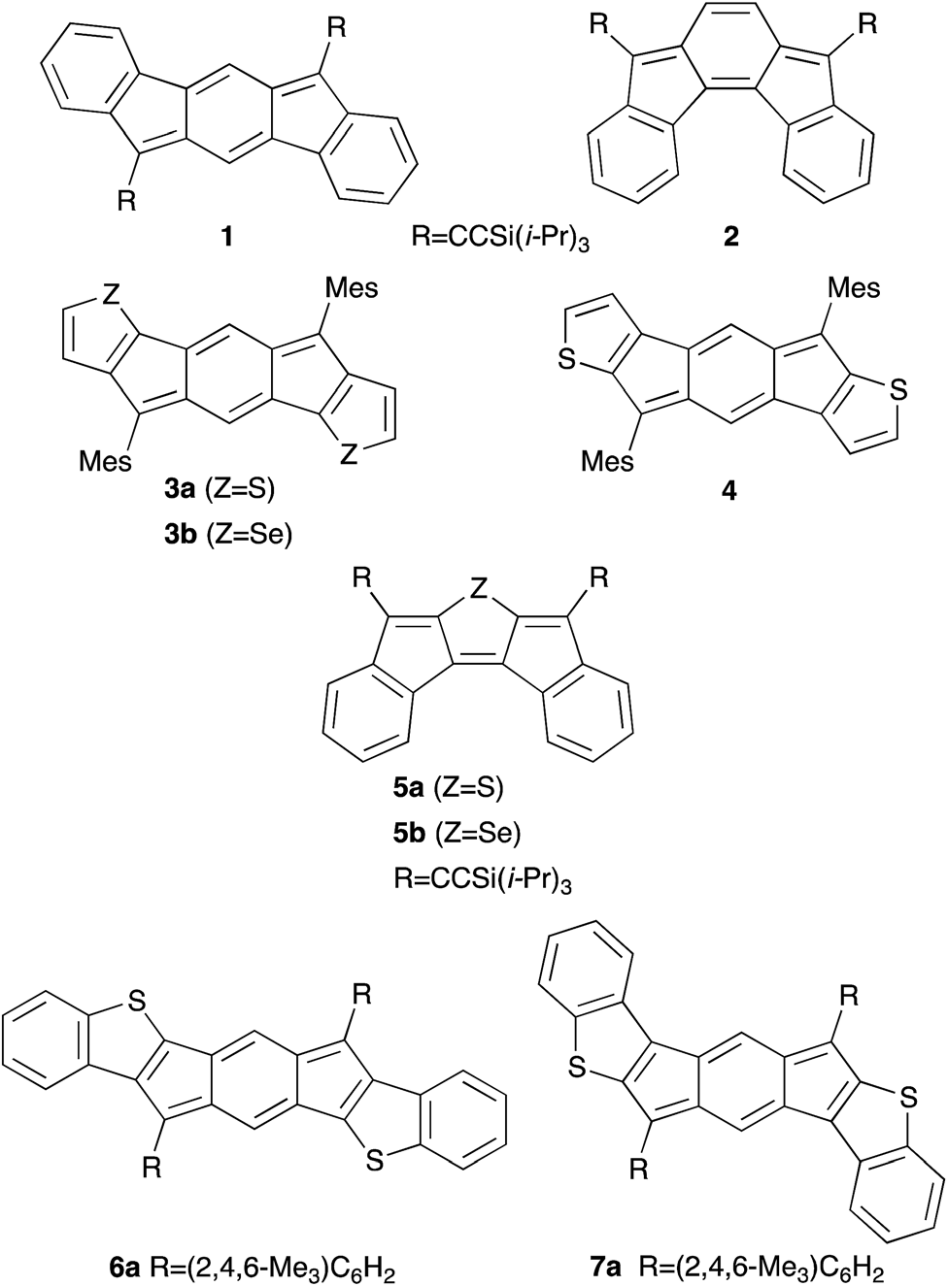
Indeno[1,2-*b*]fluorene ([1,2-*b*]IF) **1**, indeno[2,1-*c*]fluorene ([2,1-*c* IF) **2**, *anti*-indacenodithiophene (*anti*-IDT) **3a**, indacenodiselenophene (IDS) **3b**, *syn*-indacenodithiophene (*syn*-IDT) **4**, diindenothiophene (DI1T) **5a**, diindenoselenophene (DIS) **5b**, *anti*-indacenodibenzothiophene (*anti*-IDBT) **6a**, and *syn*-indacenodibenzothiophene (*syn*-IDBT) **7a**.

Although acenes are widely used in organic electronics, their susceptibility to oxidative and photolytic degradation has led researchers to explore alternative acene-like topologies.7j,9d,^11^ Studies on acene-like structures reveal that decreasing the aromaticity within a ring system or the inclusion of antiaromatic rings into materials could greatly improve their electrical conductivity and increase charge mobilities in the solid state.4e,5b,^6^,^12^ Recently, indenofluorenes, with their 6–5–6–5–6 fused ring systems and overall 4n π electrons, have received particular interest as indeno[1,2-*b*]fluorene (**1**) derivatives show ambipolar charge transport in both single-crystal and thin-film OFETs (Fig. 1).^13^

**Scheme 1:**
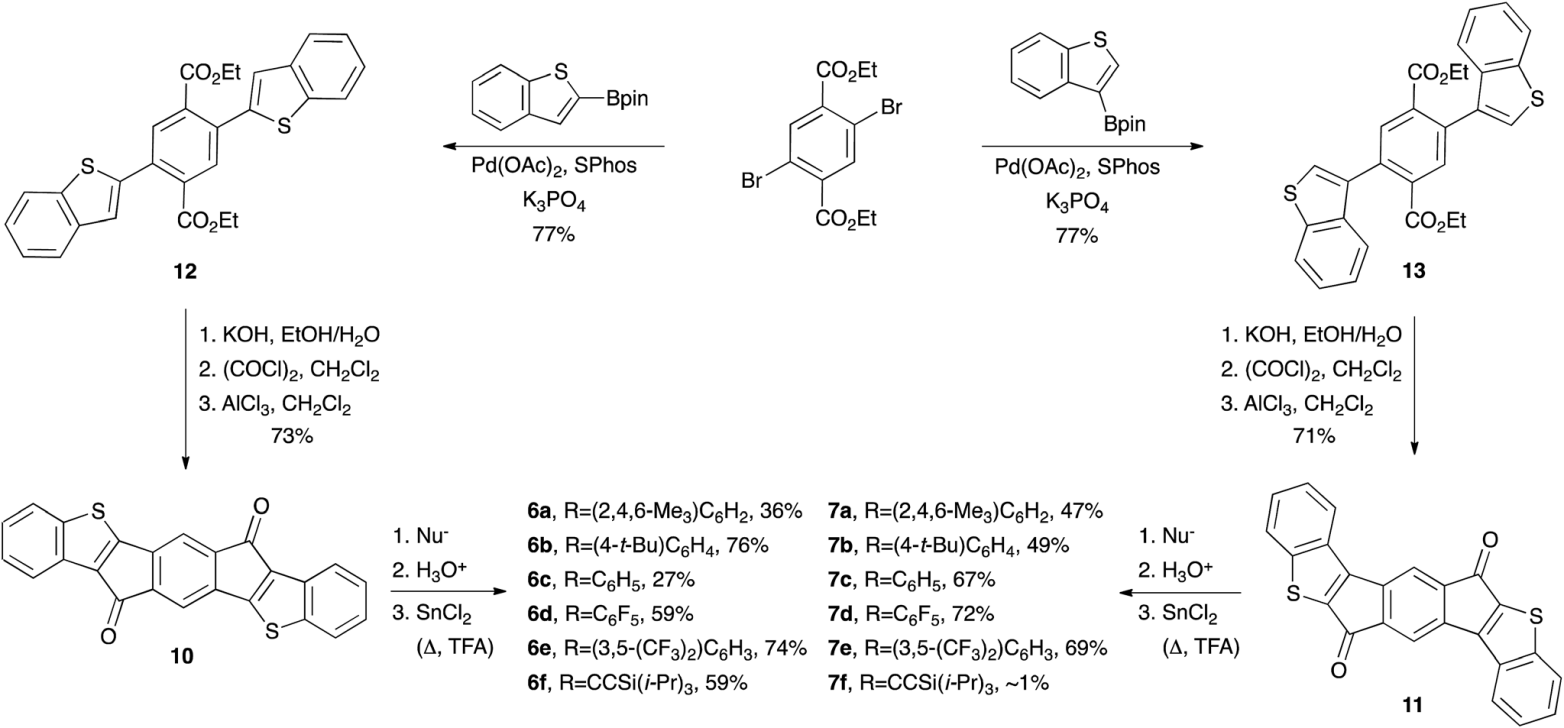
Preparation of IDBTs **6a–f** and **7a–f***via* an improved synthesis of diesters **12** and **13**.

Since 2010, our group^14^ and others10g,i,13b,^15^ have investigated compounds based on the indenofluorene (IF) scaffold (IFs **1–7**Fig. 1).13a,^16^ The inclusion of two carbonaceous five-membered rings imparts an intrinsic ability to accept electrons reversibly,16*i* which in turn has led to the examination of IFs and their derivatives as potential ambipolar and n-type materials. Recently, we explored thieno-fusion on the IF skeleton and published initial reports on the synthesis and characterization of indacenodithiophene and indacenodibenzothiophene (*anti*-IDT **3a** and *syn*-IDT **4**, *anti*-IDBT **6a** and *syn*-IDBT **7a**),16*l* diindenothienoacenes (DI[*n*]T **5a**),16*k* and their selenophene analogues (IDS **3b** and DIS **5b**)16*f* (Fig. 1). Given the low-lying LUMO levels and small electrochemical energy gaps of IDBTs **6a** and **7a**, we were keen to further expand the chemistry of these compounds. We also sought to explore more promising crystal morphologies for the IDBTs by varying the substituents at the apical carbon of the five-membered ring. Herein we report a combined synthetic, computational, structural, and materials study of *anti*-IDBTs **6** and *syn*-IDBTs **7**. We describe in detail the pronounced antiaromaticity of the *s*-indacene core, the preparation of IDBTs **6b–f** and **7b–f** (Scheme 1) along with the improved synthesis of their respective precursors (IDBT diones **10** and **11**), and finally the respective optical, electrochemical, solid-state, and materials properties of the new compounds.

## Results and discussion

### NICS-XY scan computations

**Fig. 2:**
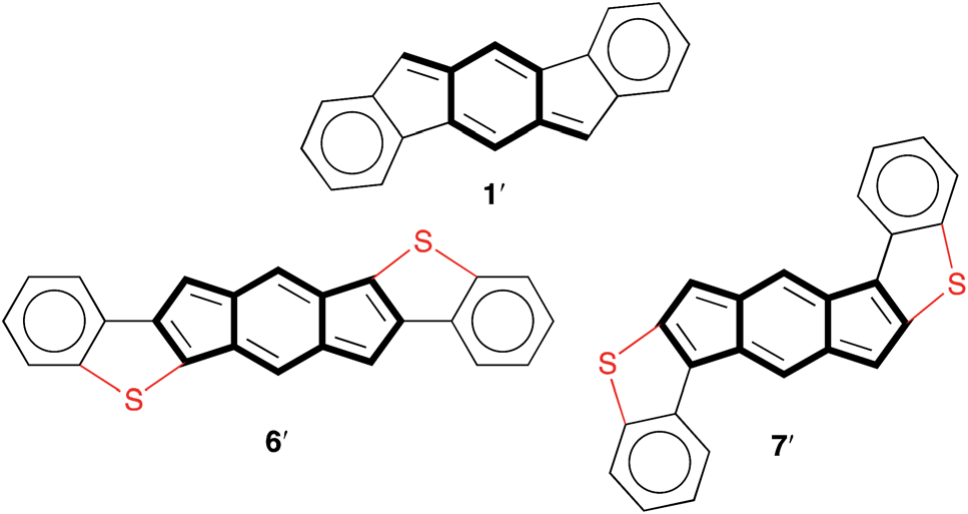
Molecular structures highlighting the *para*-xylylene motif in [1,2-*b*]IF **1′** and the *s*-indacene motif in IDBTs **6′** and **7′**.

**Fig. 3:**
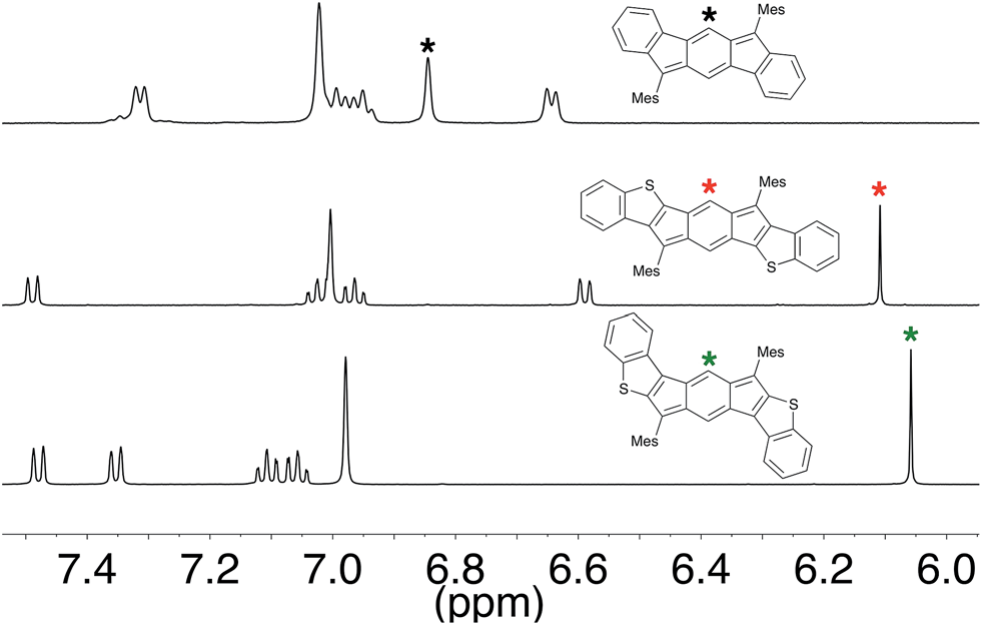
Partial ^1^H NMR spectra (500 MHz, CD_2_Cl_2_, 20 °C) of compounds **1**, **6a**, and **7a**.

Despite the indenofluorene naming convention (*i.e.*, an indene fused to a fluorene), analysis of the ^1^H NMR spectrum,16*i* NICS values and X-ray crystal structure data16a,b of indeno[1,2-*b*]fluorene (**1**) reveal that this compound is more accurately described as a benzo-fused *para*-xylylene derivative (**1′**, Fig. 2). Both *anti*-IDBT **6a** (6.11 ppm) and *syn*-IDBT **7a** (6.06 ppm) show a significant upfield ^1^H NMR chemical shift of the central six-membered ring proton when compared with [1,2-*b*]IF **1** (6.85 ppm) (Fig. 3), suggesting increased paratropicity of the *s*-indacene core of the IDBTs. Given the difficulty of assessing aromaticity/antiaromaticity simply based on NMR chemical shifts alone, we elected to explore computationally the antiaromaticity of IDBTs **6′** and **7′**.

Determination of the NICS value offers a means to assess the aromaticity or antiaromaticity of a ring system.4a–d Negative NICS values indicate a diatropic ring current (aromatic), while positive NICS values indicate a paratropic ring current (antiaromatic). Although NICS values are useful for determining local ring currents, they are unable to expound upon global and semi-global ring currents. Recently, Stanger developed the NICS-XY scan, which can be used to explore these types of ring currents (global, semi-global, local, diatropic or paratropic) in flat π-conjugated systems.^17^

**Fig. 4:**
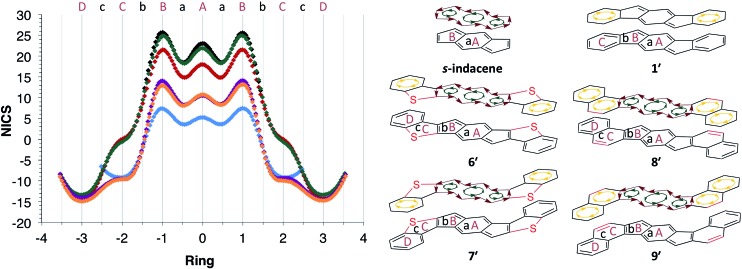
NICS-XY scans and induced ring currents of *s*-indacene (black), [1,2-*b*]IF **1′** (blue), *anti*-IDBT **6′** (red), *syn*-IDBT **7′** (green), *anti*-IDN **8′** (purple) and *syn*-IDN **9′** (orange).

The NICS-XY scans of *s*-indacene, [1,2-*b*]IF **1′**, *anti*-IDBT **6′** and *syn*-IDBT **7′** are shown in Fig. 4, along with those of the isoelectronic hydrocarbon analogues of the IDBTs, namely unknown indacenodinaphthalenes (IDNs) **8′** and **9′**. The B3LYP/6-311+G* NICS-XY values were taken 1.7 Å above the molecular plane and employed the σ-only model to take only π contributions into consideration.^17^,^18^ The NICS-XY scan of *s*-indacene shows two strong (25.7 ppm) paratropic ring currents on the five-membered rings (B rings) and a slightly smaller (23.1 ppm) paratropic ring current over the center six-membered ring (A ring). This NICS-XY scan suggests that *s*-indacene contains a global paratropic ring current as well as local paratropic ring currents over each of the rings with the current over the outer five-membered B rings being the strongest. In contrast, the NICS-XY scan of **1′** reveals two diatropic ring currents (–9.2 ppm) that are clearly visible over the outer most benzene rings (C ring), while the central *s*-indacene motif is weakly paratropic (largest NICS value of 7.4 ppm). This result further confirms our assertion that [1,2-*b*]IF **1′** is two aromatic benzene rings fused to a weakly paratropic *para*-xylylene core.

The pronounced paratropicity of the *s*-indacene core of IDBTs **6′** and **7′** is noticeably evident from their respective NICS-XY scans. *anti*-IDBT **6′** is slightly less paratropic (NICS values of 21.5 ppm on B ring and 18.0 ppm on A ring) than *s*-indacene while showing an overall similar NICS-XY scan. The NICS-XY scan of the *syn*-IDBT **7′** core is nearly indistinguishable from that of the parent *s*-indacene. *syn*-IDBT **7′** possesses NICS-XY values of 24.9 ppm and 21.9 ppm for the B ring and A ring, respectively, compared with 25.7 and 23.1 ppm for *s*-indacene. Both **6′** and **7′** have strong, nearly equal diatropic ring currents (–14.3 and –13.3 ppm, respectively) in the outer-most benzene rings (D ring), while the thiophene ring (C ring) of both **6′** and **7′** is clearly non-aromatic with NICS values of 0.07 and 0.11 ppm, respectively. In contrast, the NICS values for the *s*-indacene core in isoelectronic *anti*-IDN **8′** and *syn*-IDN **9′** are roughly half of the IDBT values—B ring and A ring values are 14.0 and 10.5 ppm for **8′** and 12.9 and 10.6 ppm for **9′**, respectively, reflecting the competition between the paratropic and diatropic ring currents. The diatropic ring currents in the C and D rings of **8′** and **9′** (**8′**: –8.7/–14.1, **9′**: –9.4/–14.9 ppm, respectively), however, are analogous to the NICS values predicted for the C ring of **1′** and the D ring of **6′** and **7′**, as might be expected for the purely hydrocarbon naphthalene unit.

### ACID calculations

**Fig. 5:**
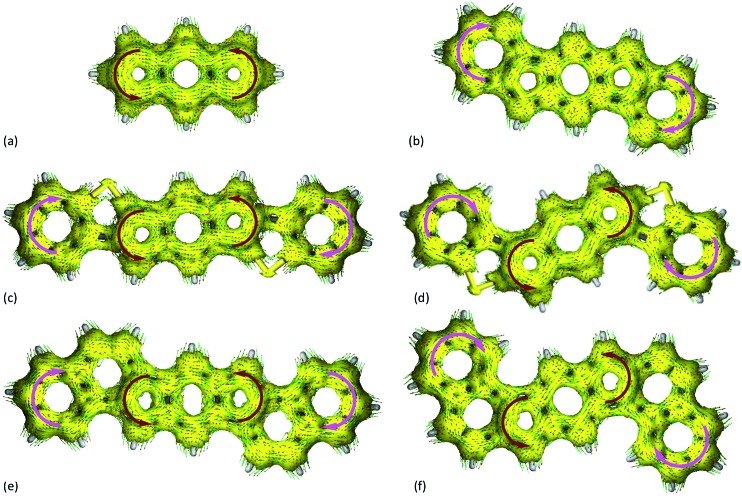
ACID plots of the induced ring currents of (a) *s*-indacene, (b) indeno[1,2-*b*]fluorene **1′**, (c) *anti*-IDBT **6′**, (d) *syn*-IDBT **7′**, (e) *anti*-IDN **8′** and (f) *syn*-IDN **9′**. Note that the magnetic field is chosen to be orthogonal to the ring planes and pointing towards the viewer.

The anisotropy of induced current density (ACID) method^19^ is used to visualize the ring currents^20^ for *s*-indacene, **1′**, and **6′–9′** at the TPSSh/SVP level of theory (Fig. 5).^21^ According to the ACID calculations, the parent *s*-indacene exhibits strong antiaromatic character (shows a counter-clockwise ring current) (Fig. 5a). A critical isosurface value (CIV) can be assigned to weak points in a cyclic system of delocalized electrons.‡
‡It should be noted that NICS-XY values and ACID plot CIV values for antiaromatic compounds are exaggerated and should be treated with some caution. The results presented in this paper should not be seen as a definitive value of antiaromaticity, but rather as a method for comparison between similar systems.
22 A high CIV represents strong conjugation, and a low CIV reflects weak delocalization at a critical point. In the case of *s*-indacene, the isosurface ruptures at a CIV of 0.111 (see ESI† for all CIV data). In contrast, the ACID plot for [1,2-*b*]IF **1′** exhibits a diamagnetic ring current (aromatic) in the outer benzene rings that seem to disturb the paramagnetic ring current of the indacene core (Fig. 5b). The isosurface in the indacene core ruptures earlier (CIV = 0.071) than in that of *s*-indacene, further confirming that [1,2-*b*]IF **1′** is weakly paratropic.

The ACID plot of *anti*-IDBT **6′** exhibits diamagnetic ring currents in the outer benzene rings and a strong paratropic ring current in the indacene core (Fig. 5c). Similarly, the ACID plot of *syn*-IDBT **7′** also exhibits diamagnetic ring currents in the outer benzene rings and a strong paratropic ring current in the indacene core (Fig. 5d). The isosurface of the *s*-indacene core for **6′** ruptures at a CIV of 0.084 and at a CIV of 0.099 for *syn*-IDBT **7′**. The trend of CIVs nicely matches the trend seen in the NICS-XY scans, namely, the antiaromaticity of the *s*-indacene core increases from *anti*-IDBT **6′** to *syn*-IDBT **7′** with **7′** being nearly as antiaromatic as the parent *s*-indacene. For IDNs **8′** and **9′** (Fig. 5e and f) the CIV are 0.076 and 0.074, respectively, falling in-between the values of **1′** and **6′**/**7′**, analogous to the NICS-XY scans (see ESI† for more details).

The combined ^1^H NMR data, NICS-XY scans, and ACID results paint a fairly clear picture of the antiaromatic nature of IDBTs **6′** and **7′**. The NICS-XY and ACID calculations also reconfirm the strong paratropicity of *s*-indacene while further solidifying our argument that this same structural unit within [1,2-*b*]IF **1′** be regarded as weakly paratropic. All three data sets show increasing antiaromaticity of the tricyclic core from [1,2-*b*]IF **1′** to IDNs **8′** and **9′**, then to *anti*-IDBT **6′** and *syn*-IDBT **7′** with the parent *s*-indacene showing the highest degree of paratropicity. Direct benzannelation of the indacene core (as in **1′**) strongly reduces the antiaromaticity of the indacene unit because the diatropic (clockwise) ring current of benzene encounters the paratropic (counter-clockwise) current of the indacene at the bond of annelation (and the benzene obviously dominates the conflicting currents). This conflict is mitigated somewhat in naphtho-fused IDNs **8′** and **9′**; however, the thiophene rings in **6′** and **7′** essentially isolate the conflicting ring currents, leaving the paratropic *s*-indacene current almost intact. Given these results, we believe that IDBTs **6′** and **7′** are best described as substituted *s*-indacenes with the appended phenyl groups planarized by thioether linkages (Fig. 2, 4). Although not a significant difference, calculations at the B3LYP/cc-pVDZ level of theory indicate that **6′** is more stable than **7′** by 1.29 kcal mol^–1^.

### Synthesis

Encouraged by the computational studies, we set out to prepare new derivatives of **6** and **7**. The typical strategy for the synthesis of IFs and their related congeners is addition of a nucleophile to the corresponding dione followed by a SnCl_2_-mediated reductive dearomatization. Our initial report on the assembly of diones **10** and **11** utilized a Stille cross-coupling to construct key precursors **12** and **13**, respectively.16*l* We have improved the preparation of diesters **12** and **13** by employing a Suzuki–Miyaura cross-coupling (Scheme 1), allowing us to selectively synthesize diones **10** or **11** on gram scale from commercially available starting materials without the use of column chromatography. Concurrent work on a separate project suggested that performing the final SnCl_2_-mediated reduction under rigorous anhydrous and anaerobic conditions could induce a significant increase in product yield. As a prototypical example, the synthesis of compound **6f** proceeds in 12% yield when using N_2_-sparged toluene as received, whereas performing the final reduction under rigorous anaerobic and anhydrous conditions furnishes **6f** in 59% yield. This strategy was extended to a series of diaryl- and diethynyl-substituted IDBTs possessing electron-withdrawing or donating groups (**6a–f**, **7a–f**).§
§In our hands, isolation of **7f** proved to be highly problematic as we were unable to obtain more than 1–2 mg of moderately pure material. Further attempts at purification resulted in complete degradation of the material and multiple attempts were necessary to produce the data provided in this manuscript. The reduction for electron-withdrawing arenes such as (**6d**, **e** and **7d**, **e**) was sluggish and required the addition of a small amount of trifluoroacetic acid for the reaction to proceed smoothly.

### Optoelectronic properties

**Fig. 6:**
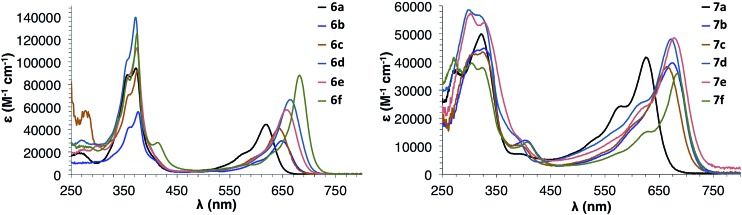
Electronic absorption spectra of *anti*-IDBTs **6a–f** (left) and *syn*-IDBTs **7a–f** (right) in CH_2_Cl_2_.

Fig. 6 shows the electronic absorption spectra for IDBTs **6a–f** and **7a–f**. These data, along with the experimentally determined electrochemical data, are summarized in Table 1. *anti*-IDBTs **6a–f** have a major absorption centered around 375 nm (3.31 eV) while *syn*-IDBTs **7a–f** exhibit a major absorption from 330 to 300 nm (3.76 to 4.13 eV). As with the previously reported mesityl derivatives **6a** and **7a**, **6b–f** and **7b–f** show low energy absorptions ranging from 683 to 643 nm (1.82 to 1.93 eV). Time-dependent density functional theory (TDDFT) calculations at the B3LYP/cc-pVDZ level of theory show that these S_0_ → S_2_ one-electron excitations are predominantly HOMO → LUMO or HOMO–1 → LUMO transitions, depending on the nature of the substituent appended to the IDBT backbone (see ESI† for more details).

**Table 1:** Electrochemical and optical data for IDBTs **6a–f** and **7a–f**

Compd	Electrochemical						Optical^*a*^		
	*E* 1 red (V)	*E* 2 red (V)	*E* 1 ox (V)	*E*_HOMO_ (eV)	*E*_LUMO_ (eV)	*E*_gap_ (eV)	*λ*_max_ (nm)	*λ*_onset_ (nm)	*E*_gap_ (eV)
**6a** ^*b*^ ^,^ ^*d*^	–0.80	–1.62^*c*^	0.92	–5.56	–3.84	1.72	618	649	1.91
**6b** ^*b*^ ^,^ ^*e*^	–0.64	–1.40^*c*^	1.01	–5.56	–4.00	1.65	648	685	1.81
**6c** ^*b*^ ^,^ ^*d*^	–0.61	–1.32^*c*^	1.08^*c*^	–5.72	–4.03	1.69	643	678	1.83
**6d** ^*b*^ ^,^ ^*f*^	–0.41	–1.15	1.10	–5.74	–4.23	1.52	664	702	1.77
**6e** ^*b*^ ^,^ ^*e*^	–0.35	–0.97	1.36^*c*^	–6.00	–4.29	1.71	658	694	1.79
**6f** ^*b*^ ^,^ ^*f*^	–0.46	–1.24^*c*^	0.97	–5.61	–4.18	1.44	682	709	1.75
**7a** ^*b*^ ^,^ ^*d*^	–0.61	–1.24^*c*^	0.98^*c*^	–5.62	–4.03	1.59	626	664	1.89
**7b** ^*b*^ ^,^ ^*e*^	–0.49	–1.08	1.02	–5.66	–4.15	1.51	673	719	1.72
**7c** ^*b*^ ^,^ ^*e*^	–0.46	–1.01	1.04^*c*^	–5.68	–4.19	1.50	665	712	1.74
**7d** ^*b*^ ^,^ ^*f*^	–0.33	–0.96	1.02	–5.66	–4.31	1.36	672	715	1.74
**7e** ^*b*^ ^,^ ^*e*^	–0.16	–0.64	1.29^*c*^	–5.93	–4.48	1.46	677	722	1.72
**7f** ^*b*^ ^,^ ^*f*^	–0.37	–1.03^*c*^	1.00	–5.64	–4.27	1.38	683	718	1.73

^*a*^Spectra were obtained in CH_2_Cl_2_. The optical HOMO/LUMO gap/absorbance onset was determined as the intersection of the *x*-axis and a tangent line passing through the inflection point of the lowest energy absorption.

^*b*^CVs were recorded at a scan rate of 50 mV s^–1^ with a glassy carbon working electrode, a Pt coil counter electrode, and a Ag wire pseudo-reference. Values reported as the half-wave potential (*vs.* SCE) using the Fc/Fc^+^ couple (0.46 V in CH_2_Cl_2_, 0.56 V in THF) as an internal standard. HOMO and LUMO energy levels in eV were approximated using SCE = –4.68 eV *vs.* vacuum and *E*_1/2_ values for reversible processes or *E*_p_ values for irreversible processes.

^*c*^Reported as *V* at peak current, not half-wave potential.

^*d*^1–5 mM of analyte in 0.1 M Bu_4_NOTf/CH_2_Cl_2_.

^*e*^1–5 mM of analyte in 0.1 M Bu_4_NBF_4_/THF.

^*f*^1–5 mM of analyte in 0.1 M Bu_4_NBF_4_/CH_2_Cl_2_.

Interestingly, there are differences in the low energy absorptions of the *anti*-IDBTs compared to the *syn*-IDBTs. The *anti*-IDBTs (**6b–f**) have a *λ*_max_ ranging from 643–682 nm (39 nm [0.11 eV] range) whereas the *syn*-IDBTs (**7b–f**) have a *λ*_max_ ranging from 665 to 683 nm (18 nm [0.04 eV] range). This difference is reflected in the onset of absorbance for the two isomers: the *anti* isomers have an onset of absorption ranging from 678 to 709 nm (31 nm [0.07 eV] range) while the *syn* isomers have an onset of absorption ranging from 712 to 722 nm (10 nm [0.03 eV] range). From the computational data, however, one cannot ascertain ‘more variability’ in one IDBT family *versus* the other. For the computed S_0_ → S_1_ excitation energies, the range for IDBTs **6a–f** is 0.27 eV while that in IDBTs **7a–f** is 0.25; a similar story unfolds for the adiabatic ionization potentials (see below), where the range for IDBTs **6a–f** is 0.60 eV while that for IDBTs **7a–f** is 0.58 eV. Moreover, the trends in the S_0_ → S_1_ transition energies and adiabatic ionization potentials are nearly identical for both families.

As observed with all other reported IFs, IDBTs **6a–f** and **7a–f** are non-emissive, a fact consistent with the lack of oscillator strength (*f*) determined for the S_0_ → S_1_ transitions in all TDDFT calculations on the IDBTs.^23^ The S_0_ → S_1_ excitations in the IDBTs consist of a one-electron transition between orbitals of (nearly) the same symmetry (*b*_g_, based on the *C*_2h_ symmetry of **6′** and **7′**, see below), making the excitations orbitally forbidden.

As was seen in our study of mesityl-substituted [1,2-*b*]IF **1**, both **6a** and **7a** are hypsochromically shifted (*ca.* ∼40 nm) from **6b–f** and **7b–f**. This is easily explained by examination of the crystal structures of **7a***vs.***7b**. The mesityl groups of **6a** and **7a** are nearly orthogonal to the core of the molecule with a dihedral angle between the average planes of the aryl group and IDBT core of 75.0° and 59.6°, respectively, whereas **7b** has dihedral angles of 33.2° and 29.3°. The large dihedral angle of the mesityl group to the IDBT core in **6a** and **7a** limits electronic communication between the orthogonal π systems, effectively limiting the delocalization of the π orbitals of **6a** and **7a** when compared to the other aryl substituents (see the discussion of the molecular orbitals below for more details).

### Electrochemistry

**Fig. 7:**
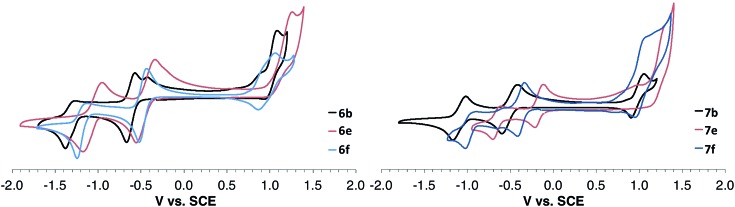
CV data of *anti*-IDBTs **6b**, **6e**, and **6f** (left) and *syn*-IDBT **7b**, **7e**, and **7f** (right).

All IDBTs undergo one reversible reduction in solution. A second reduction is observed for all of the IDBTs; however, its reversibility is dependent upon the aryl or ethynyl substituent. IDBTs **6a–f** and **7a–f** all display an oxidation wave ranging from irreversible to reversible depending on the aryl or ethynyl substituent. Cyclic voltammograms (CVs) of **6b**, **e**, **f** and **7b**, **e**, **f** are displayed in Fig. 7 (see the ESI† for CVs of **6a**, **c**, **d** and **7a**, **c**, **d**). All electrochemical data are compiled in Table 1. *E*1red values range from –0.80 to –0.16 V *vs.* SCE (**6a** and **7e**, respectively) and *E*1ox values range from 0.92 to 1.36 V *vs.* SCE (**6a** and **6e**, respectively). Electron-withdrawing groups shift *E*1red and *E*1ox to more positive values while electron-donating groups shift *E*1red and *E*1ox to more negative values.

Once again, interesting electronic differences between the *syn*- and *anti*-isomers are observed. The CV determined *E*1red values for the *syn*-IDBTs are consistently more energetically stabilized when compared to their *anti*-IDBT counterparts, and the electrochemically determined *E*_gap_ (*E*1ox – *E*1red) is consistently smaller for the *syn*-IDBTs. The *E*1red values of the diaryl *anti*-IDBTs (**6a–f**) have an average *E*1red 0.15 eV higher than their *syn*-IDBT (**7a–f**) counterparts.¶
¶For a discussion on the differences between optically determined, electrochemically-determined and computationally determined HOMO/LUMO levels, energy gaps, and their terminology, please see: J.-L. Bredas, *Mater. Horiz.* 2014, **1**, 17. As with the optical transitions, the computed ionization potentials (IP) and electron affinities (EA) at the B3LYP/cc-PVDZ level of theory follow the experimental trends quite well (see ESI†), with the *syn*-IDBTs generally possessing larger EAs (and more energetically stabilized LUMO energies) than the *anti*-IDBT counterparts with the same chemical functionalization.

Analysis of the frontier molecular orbitals provides insight behind the observed optoelectronic characteristics. Select frontier molecular orbitals for **6′** and **7′**, each with *C*_2h_ symmetry, are given in the ESI.† For each system, the splitting between the HOMO–1 (*a*_u_ symmetry) and HOMO (*b*_g_ symmetry) is rather small, 0.13 eV for **6′** and 0.26 eV for **7′**, with the HOMO energies for **6′** and **7′** nearly identical. The LUMO of **7′**, however, is 0.13 eV more energetically stable than that of **6′** (both LUMOs have *b*_g_ symmetry), a result that already provides insight into the larger EAs/smaller reduction potentials observed for the *syn*-IDBT series. In both cases, the HOMO and LUMO mainly reside on the carbon framework, with the sulfur playing a larger role in the LUMO of **6′** and the HOMO of **7′**. Notably, the various chemical substituents, depending on the relative donor or acceptor strength (and configuration of the phenyl group), impart changes to the molecular orbitals. First, the energetic gap between the HOMO and HOMO–1 is reduced across the full molecular series with respect to the parent **6′** and **7′** species, with the donor substituents in **6b**, **6c**, and **7b** leading to an energetic repositioning of the HOMO and HOMO–1, *i.e.*, the HOMO is the *a*_u_ symmetric orbital in these systems. It may be expected that the donor strength of the mesityl substituents in **6a** and **7a** could also lead to such an inversion; however, the lack of extension of the HOMO or HOMO–1 onto the phenyl rings, due to the orthogonal orientation of the mesityl units, prevents this from occurring and the frontier orbitals simply become energetically destabilized when compared to **6′** and **7′** through inductive effects. The reordering of the orbitals leads to the varying descriptions of the S_0_ → S_1_ and S_0_ → S_2_ described above. A combination of wave function delocalization on the phenyl rings and inductive effects due to the fluorine atoms in **6d**/**7d** and **6e**/**7e** lead to large energetic stabilization of the frontier orbitals, and subsequently the larger IPs/smaller oxidation potentials and larger EAs/smaller reduction potentials of these systems *versus* the other phenyl substituents. The picture differs somewhat when comparing **6f** and **7f**: in both cases, the LUMO extends onto the ethynyl arm, energetically stabilizing the LUMO when compared to the parent *anti*- and *syn*-IDBT systems. In **6f**, however, the HOMO and HOMO–1 switch their order and the *a*_u_ parent orbital extends onto the ethynyl arm, destabilizing the HOMO, while this does not appear to be the case in **7f** (though the energetic splitting between the HOMO and HOMO–1 is quite small, 0.02 eV). Overall, the molecular orbitals reveal the rather complex interplay between the base *anti*- and *syn*-IDBT structures and the nature of the chemical substituents that in turn determine the relative electrochemical and optical characteristics observed experimentally.

### Solid-state structures

**Fig. 8:**
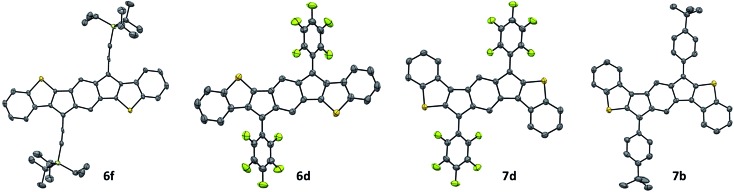
Molecular structures of **6f**, **6d**, **7d**, and **7b**; hydrogen atoms omitted for clarity. Ellipsoids drawn at 50% probability level.

**Fig. 9:**
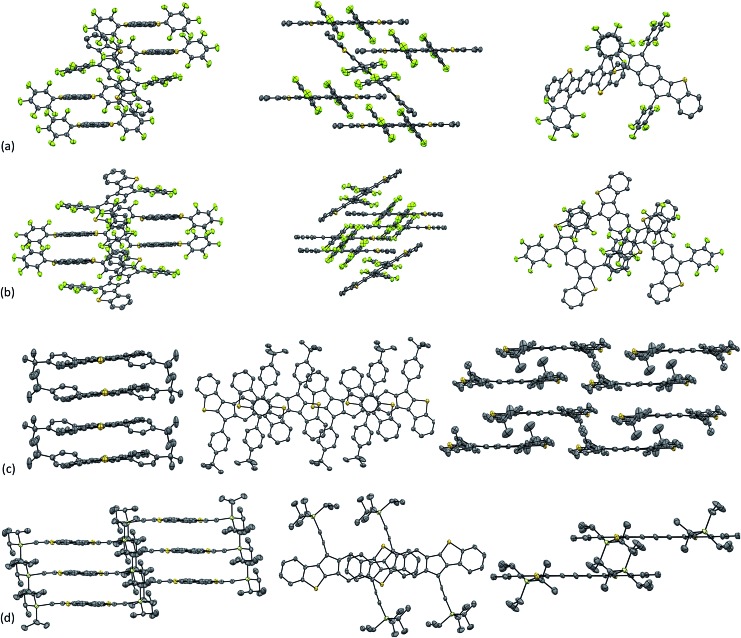
Solid state crystal packing diagrams of (a) *anti*-IDBT **6d**, (b) *syn*-IDBT **7d**, (c) *syn*-IDBT **7b**, and (d) *anti*-IDBT **6f**. Hydrogen atoms omitted for clarity. Ellipsoids drawn at 50% probability level.

Single crystals of **6f** and **7b** suitable for X-ray diffraction (XRD) were obtained *via* the slow diffusion of CH_3_CN into CHCl_3_, while single crystals of **6d** and **7d** were grown *via* the slow evaporation of a concentrated solution in chlorobenzene at –40 °C. The molecular structures of **6d**, **f** and **7b**, **d** are shown in Fig. 8. As expected, varying the substituents of the IDBT core greatly influences the observed packing motif (Fig. 9). The solid-state morphologies of both **6d** and **7d** appear to be largely driven by arene/perfluoroarene interactions (Fig. 9a and b). *anti*-IDBT **6d** packs in 1-D chains with a distance between average planes of the IDBT core of 3.48 Å. The perfluorophenyl groups of one 1-D chain are centered over the aromatic aryl group (D ring) of the neighboring chain. The distance between the center of the perfluorophenyl ring and center of the D ring is 3.76 Å, which is in good agreement with the center-to-center distances (3.4–3.8 Å) observed for other 1 : 1 arene/perfluoroarene crystal structures.^24^ The dihedral angle between the plane of the IDBT core and the plane of the perfluorophenyl ring of **6d** is 54.9°, significantly smaller than the dihedral angle between the average plane of the mesityl group and IDBT core of **6a** (75.0°).

The molecular structure of *syn*-IDBT **7d** contains three symmetrically independent molecules with two of these forming a 1-D chain. The distance between the average planes of the 1-D chain is 3.57 Å. The third molecule bridges the 1-D chains with the center of the perfluorophenyl rings of the bridging molecule 3.51–3.74 Å from the center of the D ring for the molecules of the 1-D chain. The dihedral angle between the average plane of the perfluorophenyl ring and the plane of the IDBT core ranges from 44.0–47.5°. This dihedral angle is significantly smaller than the same angle observed in **6d** and can be explained by a weak S···F interaction.^25^ The distance between the sulfur of the thiophene ring (C ring) and the nearest fluorine of the perfluorophenyl ring ranges from 2.98–3.03 Å, well below the S–F van der Waals radius of 3.27 Å.

*syn*-IDBT **7b** appears to pack on the cusp between a 1-D and 2-D structure (1-D chain of…B–A–B′…) with distances of 3.49 and 3.42 Å between the average planes (B–A and A–B′, respectively) (Fig. 9c). The close C–S contacts between 1-D columns are 3.60 and 3.51 Å, while the close C–C contacts are 3.55 and 3.45 Å (B–A and A–B′, respectively). The centroids of the IDBT cores are offset by 3.11 and 4.67 Å (B–A and A–B′). Interestingly, there appear to be multiple S–S interactions between the 1-D chains with S–S distances of 3.48, 3.65 and 3.68 Å (S–S van der Walls radius is 3.60 Å).

*anti*-IDBT **6f** packs in 1-D slip-stacked columns with a distance between the average planes of the IDBT core of 3.36 Å and short C–C contacts of 3.36 Å (Fig. 9d). There is a large amount of molecular overlap in the packing of **6f** with the center of the outer benzene ring shifted only 1.15 Å from the center of the central *s*-indacene ring. Given the large overlap and sub-van der Waals C–C contacts in **6f**, we elected to focus on this compound for potential device fabrication.

### Electronic band structure

**Fig. 10:**
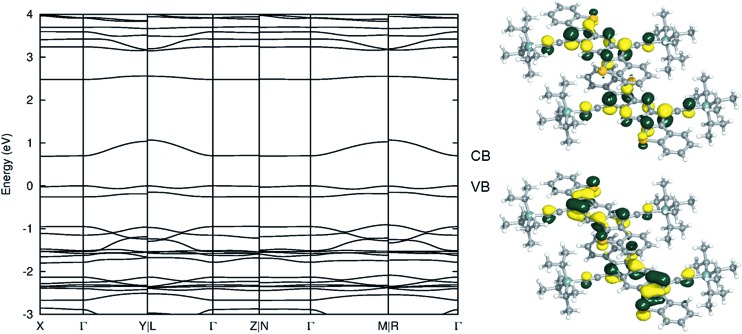
(Left) Electronic band structure for **6f** in the triclinic lattice (TRI_1a_). The valence band [VB] and conduction band [CB] are labeled for clarity. The origin of the energy axis is set at the top of VB. (Right) Pictorial representations of the **6f** dimer HOMO (bottom) and LUMO (top).

As an initial evaluation, the intermolecular electronic couplings (*t*) and electronic band structure of **6f** reveal the potential for hole and/or electron transport (the band structures for **6d** and **7d** are in the ESI†). Using the fragment orbital approach^26^ at the B3LYP/cc-pVDZ level of theory, the HOMO:HOMO electronic coupling (for hole transport) of the π-stacked dimer is found to be 50 meV, while the LUMO:LUMO electronic coupling (for electron transport) is twice as large at 100 meV. These electronic couplings are on par with those of other high performing organic semiconductor materials. Examination of the electronic band structure along various crystallographic directions of the triclinic (TRI_1a_) lattice (Fig. 10), using the experimental unit cell and atomic coordinates, reveals that the valence band dispersion is 78 meV, while the conduction band dispersion is 383 meV; relaxation of the atomic coordinates leads to a decrease of the band gap, while the band dispersions remain unaffected (see the ESI† for further details). Note that the valence band maximum is at *Γ*, while the conduction band minimum is at *X*; therefore, the band gap is indirect, but only because the conduction band following *Γ*–*X* is slightly sloped downwards (the *Γ*–*Γ* band gap is marginally larger). Overall, the magnitudes of the electronic couplings (and band widths) suggest that **6f** could possess reasonable hole and electron transport characteristics.

### Organic field effect transistors

**Fig. 11:**
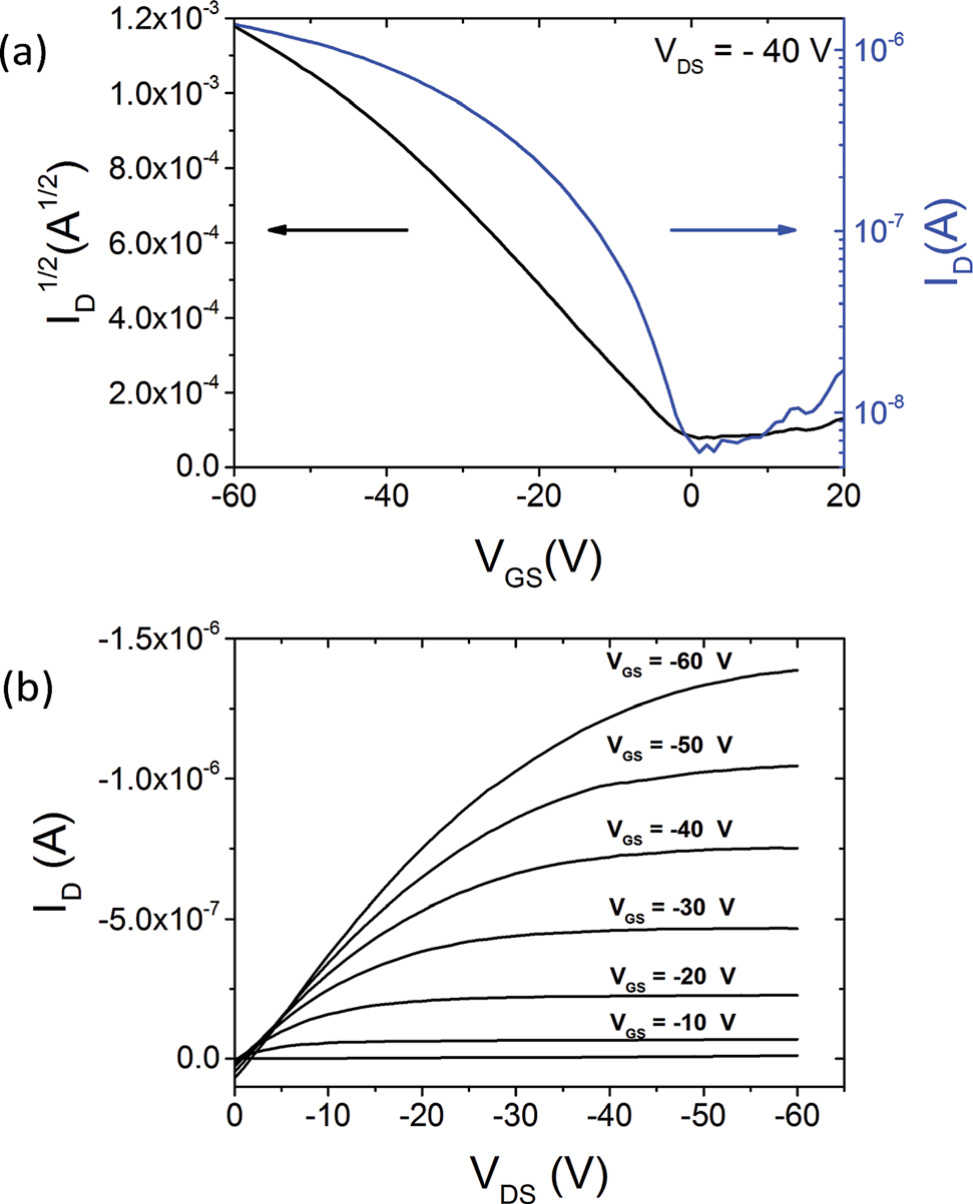
Transfer (a) and transport (b) characteristics of an OFET using *anti*-IDBT **6f**. This device demonstrated a hole mobility of 0.44 cm^2^ V^–1^ s^–1^, and a threshold voltage of *V*_th_ = 1.33 V.

Top-gate, bottom-contact OFETs of **6f** were fabricated on SiO_2_ substrates. Source and drain contacts were patterned by photolithography, with 5 nm of Ti and 40 nm Au deposited by e-beam evaporation. *anti*-IDBT **6f** (1 wt% in room-temperature chlorobenzene) was then spin-coated onto this substrate. Undiluted Cytop fluoropolymer was used as a gate dielectric and spin-coated over the organic semiconductor. Our earlier work has shown that these processing conditions yield a dielectric thickness of 1400 nm.^27^ The OFETs were subsequently annealed at 110 °C for one hour in a vacuum oven to cross-link the dielectric layer and left to cool overnight. A 60 nm layer of Au was thermally evaporated through a shadow mask aligned over the conduction channel, and this served as the gate electrode. The resulting OFETs were measured under ambient conditions, and an example for current–voltage characteristics is included in Fig. 11. We calculated the field-effect mobility *μ* from the saturation regime of device operation, at an applied drain-to-source voltage of *V*_DS_ = –40 V by standard procedures.^28^Fig. 11a shows the evolution of the drain current *I*_D_ with the applied gate-to-source voltage *V*_GS_ for this particular device of channel length *L* = 20 μm and channel width *W* = 35 μm. This device exhibits a hole mobility of *μ* = 0.44 cm^2^ V^–1^ s^–1^, and a threshold voltage of *V*_th_ = 1.33 V. Measurements over 10 devices yielded an average hole mobility of *μ*_avg_ = 0.14 cm^2^ V^–1^ s^–1^ ± 0.12 cm^2^ V^–1^ s^–1^. The low threshold voltage indicates a low density of trap states at the semiconductor–dielectric interface, which is to be expected for the transistors with Cytop gate dielectric.^27^Fig. 11b shows the transport characteristics for the same device. The transition from linear to saturation regime is clear for all investigated gate voltages. The non-linearity in the low-voltage region may originate from parasitic contact effects.^29^ These contact effects could be minimized by using surface treatments on the contacts which could also increase the measured mobility of devices; therefore, the values quoted for charge carrier mobility should be seen as a lower bound. The electrical measurements show hole-only charge transport. The lack of a measured electron transport likely originates from the fact that Au contacts were used, and while the ∼5 eV work function of Au aligns well with the estimated solid-state IP of **6f**, the energy barrier to inject electrons is too large (see Table 1).^30^

## Conclusions

In summary, our analysis of the NICS-XY scans and ACID plots of IDBTs **6′** and **7′** indicate a strong antiaromatic ring current in the central *s*-indacene core of these molecules, and that IDBTs **6** and **7** are best thought as phenyl-substituted *s*-indacene derivatives with thioether linkages planarizing the appended phenyl groups to the indacene core. We have improved the synthesis of dione precursors **10** and **11** to yield multigram quantities without the use of column chromatography and demonstrated that up to 500 mg (**6f**) of the final IDBT can be produced in one batch. We have undertaken a detailed experimental and computational analysis of IDBT **6a–f** and **7a–f** and demonstrated that the optoelectronic, electrochemical characteristics and the solid-state morphology can be significantly altered by the choice of aryl/ethynyl substitution. Analysis of the intermolecular electronic couplings and electronic band structure of **6f** indicated that it could perform well as the active element in an OFET device. Fabrication of a device with **6f** produced the highest hole mobilities recorded to date using a fully conjugated indenofluorene derivative. Future work will focus on further exploration of the antiaromatic properties of IDBTs **6** and **7** as well as benzothiophene fusion as a means to control aromaticity/antiaromaticity in other IF related structures.

## Supplementary Material

Supplementary information

Crystal structure data
