# Supplementary material for: Indacenodibenzothiophenes: synthesis, optoelectronic properties and materials applications of molecules with strong antiaromatic character
Source: Chem Sci. 2016 May 13;7(8):5547–58. doi: 10.1039/c6sc00950f (PMC5207214; doi:10.1039/c6sc00950f)
Supplement: Supplementary file 1 [file SC-007-C6SC00950F-s001.pdf]

Electronic Supplementary Material (ESI) for Chemical Science.
This journal is © The Royal Society of Chemistry 2016

Electronic Supplementary Information for:

Indacenodibenzothiophenes: Synthesis, Optoelectronic Properties and Materials Applications of Molecules With Strong Antiaromatic Character

Jonathan L. Marshall,[†] Kazuyuki Uchida,[‡] Conerd K. Frederickson,[†] Christian Schütt,[§] Andrew M. Zeidell,[±] Katelyn P. Goetz,[±] Lev N. Zakharov,[II] Chad Risko,[#] Rainer Herges,[§] Oana D. Jurchescu,[±] and Michael M. Haley[†]*

[†] Department of Chemistry & Biochemistry and Materials Science Institute, University of Oregon, Eugene, Oregon 97403-1253, United States

[‡] Department of Chemistry, Graduate School of Science, Osaka University, Toyonaka, Osaka 560-0043, Japan

[§] Institute of Organic Chemistry, University of Kiel, Otto-Hahn-Platz 4, Kiel 24098, Germany

[±] Department of Physics, Wake Forest University, Winston-Salem, North Carolina 27109, United States

[II] CAMCOR, University of Oregon, Eugene, Oregon 97403-1433, United States

[#] Department of Chemistry, University of Kentucky, Lexington, Kentucky 40506, United States

Table of Contents **page**

Experimental Details	S2
Cyclic Voltammetry	S9
X-ray Crystallography	S11
NICS-XY Scan Computations	S13
Cartesian Coordinates and Energy Data For NICS-XY Geometries	S15
ACID Plot Computations	S21
Cartesian Coordinates of TPSSh/SVP Optimized Structures	S26
DFT and TDDFT Computational Details	S29
References	S35
Copies of NMR Spectra	S37

Experimental Details

General. All air-sensitive manipulations were carried out under an inert atmosphere using either standard Schlenk technique or an N_2-filled drybox. For air sensitive reactions, THF and toluene were refluxed with Na benzophenone ketyl for 24 h prior to distillation and use. For all manipulations performed in an N_2-filled drybox, THF and toluene were refluxed with Na benzophenone ketyl for 24 h prior to distillation and then degassed via freeze pump thaw cycles. Dichloromethane and chlorobenzene were dried over CaH_2 overnight, distilled under N_2 and then degassed via freeze pump thaw cycles. For column chromatography, silica gel (240-300 mesh) was used. All other reagents were purchased from Sigma-Aldrich, TCI, Strem, Matrix Chemicals, or GFS Chemicals and used as received without further purification. NMR spectra were recorded on a Varian Inova 500 (^{1}H: 500 MHz, ^{13}C: 126 MHz), a Bruker Avance III HD 500 equipped with a Prodigy multinuclear cryoprobe (^{1}H: 500 MHz, ^{13}C: 126 MHz) or Bruker Avance III HD 600 equipped with a Prodigy multinuclear cryoprobe (^{1}H: 600 MHz, ^{13}C: 151 MHz) NMR spectrometers. ^{1}H and ^{13}C chemical shifts (δ) are expressed in ppm relative to the residual $CHCl_3$ (^{1}H: 7.26 ppm, ^{13}C: 77.16 ppm), $CHCl_2CHCl_2$ (^{1}H: 6.00 ppm, ^{13}C: 73.78), C_6H_6 (^{1}H: 7.16 ppm), and CH_2Cl_2, (^{1}H: 5.32 ppm, ^{13}C: 54.00 ppm) reference. ^{19}F chemical shifts (δ) are expressed in ppm and were externally referenced to α,α,α-trifluorotoluene (−63.72 ppm). UV-Vis spectra were recorded on an Agilent Technologies Cary 60 UV-Vis spectrometer in HPLC grade CH_2Cl_2. High-resolution mass spectra were recorded on a JEOL MS-Route mass spectrometer at the Mass Spectrometry Facilities and Services Core of the Environmental Health and Sciences Center at Oregon State University. From intermediate diesters **12** and **13**, diones **10** and **11** were synthesized, respectively, according to a previously described procedure.[1]

In several instances, the high insolubility of the IDBTs hindered acquisition of NMR spectra. To obtain suitable spectra for compounds **6b-e** and **7b**,**c**,**e**, the spectra were obtained at 70 °C (upper temperature limit for the multinuclear cryoprobe) or 100 °C in 1,1,2,2-tetrachloroethane-*d*$_2$ ($CDCl_2CDCl_2$). The peaks of interest were often of the same magnitude as the ^{13}C satellite peaks of the deuterated solvent. These requirements have the side effect of greatly exaggerating impurities present in the 99.8% 1,1,2,2-tetrachloroethane-*d*$_2$ or H grease impurities present in the samples from our Schlenk line.

Improved Synthesis of Diester Intermediates 12 and 13

General Procedure A: A three necked round bottomed flask fitted with a condenser was charged with diethyl 2,5-dibromoisophthalate[2] (3.50 g, 9.20 mmol, 1 equiv.), the appropriate benzothiophene cross-coupling partner (5.75 g, 22.10 mmol, 2.4 equiv.), SPhos ligand (0.153 g, 0.368 mmol, 0.04 equiv.), K_3PO_4 (12.08 g, 36.8 mmol, 4.4 equiv.) and toluene/H_2O (10:1, 120 mL). After bubbling N_2 through this mixture for 1 h, $Pd(OAc)_2$ (0.0413 g, 0.184 mmol, 0.02 equiv.) was added and the reaction vessel was purged for an additional 10 min. After refluxing overnight and cooling to room temperature, the reaction was quenched with H_2O and extracted with Et_2O (3×). The combined organic layers were then washed with brine (3×), dried ($MgSO_4$) and concentrated in vacuo. Recrystallization of the crude mixture from EtOH/ chlorobenzene yielded pure white crystals of the desired cross-coupled product in excellent yield.

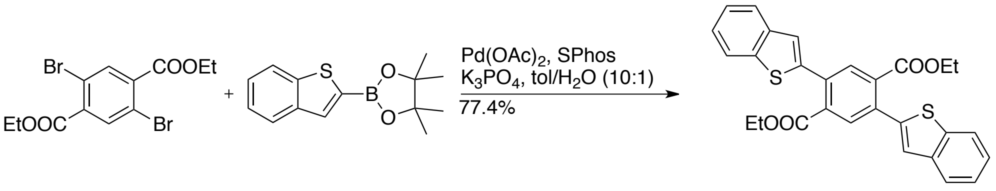

***anti*-Diester 12:** Following general procedure A with 2-(4,4,5,5-tetramethyl-1,3,2-dioxaborolan-2-yl)benzo[*b*]thiophene[3] as the cross-coupling partner furnished diester **12** (3.46 g, 77%). Spectroscopic data matched those previously reported.[1]

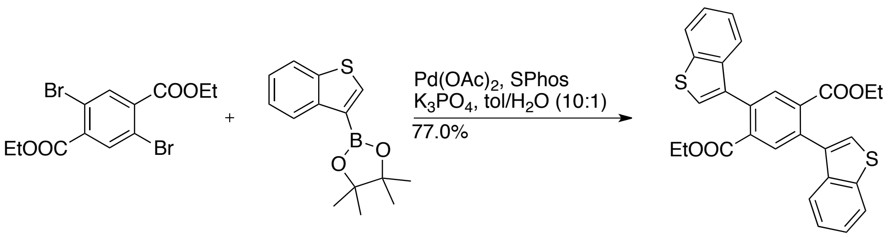

***syn*-Diester 13:** Following general procedure A with 3-(4,4,5,5-tetramethyl-1,3,2-dioxaborolan-2-yl)-benzo[*b*]thiophene[3] as the cross-coupling partner furnished diester **13** (3.45 g, 77%). Spectroscopic data matched those previously reported.[1]

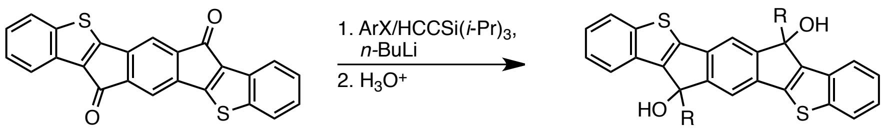

10

S1, R = (4-*t*-Bu)C_6H_4
S2, R = C_6H_5
S3, R = (3,5-$(CF_3)_2$)C_6H_3
S4, R = CCSi(*i*-Pr)$_3$

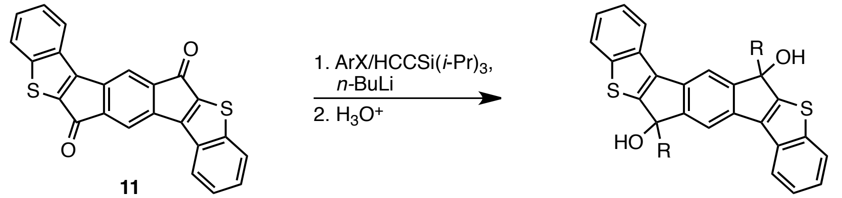

11

S5, R = (4-*t*-Bu)C_6H_4
S6, R = C_6H_5
S7, R = (3,5-$(CF_3)_2$)C_6H_3
S8, R = CCSi(*i*-Pr)$_3$

General Procedure B for diols S1-S8: In an oven-dried round bottomed flask, a suspension of dione (1 equiv.) in dry THF was cooled to −78 °C under a N_2 atmosphere. In a separate oven-dried round bottomed flask, the appropriate haloarene or silyl-protected acetylene (6 equiv.) was dissolved in dry THF, cooled to −78 °C under a N_2 atmosphere, and BuLi (2.5 M in hexanes, 5.5 equiv.) was added dropwise. This mixture was stirred at −78 °C for 1 h after which it was transferred via cannula to the flask containing the dione. This reaction mixture was slowly warmed to rt with stirring overnight. The reaction was then quenched with a saturated aq. NH_4Cl solution and extracted with Et_2O (3×). The combined organic layer was washed with brine (3×) dried ($MgSO_4$) and concentrated in vacuo. The resulting crude oil was passed through a silica-gel plug (4:1 hexanes/EtOAc) and then carried onto the reductive dearomatization without further purification or characterization.

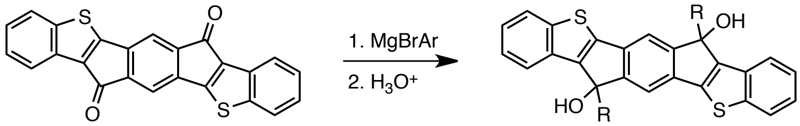

10

S9, R = (2,4,6-Me_3)C_6H_2
S10, R = C_6F_5

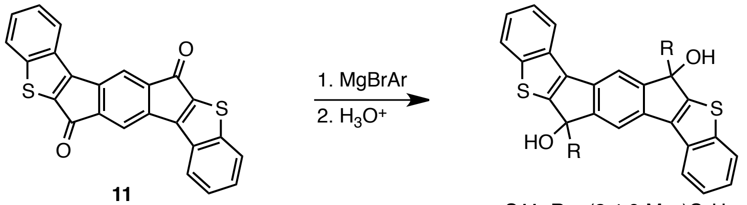

11

S11, R = (2,4,6-Me_3)C_6H_2
S12, R = C_6F_5

General Procedure C for diols S9-S12: To a stirred suspension of dione (1 equiv.) in THF (0.01 M) at −20 °C under a N_2 atmosphere, the appropriate Grignard reagent (1.0 M in THF, 10 equiv) was added dropwise. The mixture was then slowly warmed overnight to rt with stirring. The reaction was then quenched with a saturated NH_4Cl solution and then extracted with Et_2O (3×). The combined organic fractions were then washed with brine (3×), dried ($MgSO_4$) and concentrated *in vacuo*. The resulting crude oil was passed through a silica-gel plug (4:1 hexanes/EtOAc) and then carried onto the reductive dearomatization step without further purification or characterization.

General Procedure D for the reductive dearomatization of diols S1-S12 to IDBTs 6a-f and 7a-f: In an N_2-filled dry box, the diol (1 equiv.) was dissolved in dry degassed toluene (12.7 mM). Anhydrous $SnCl_2$ (4 equiv.) was added and this mixture was then vigorously stirred. For compounds **6d**,**e** and **7d**,**e**, trifluoroacetic acid (10 equiv.) was added to the reaction mixture. For compounds **6b-e** and **7b-e**, the reaction mixture was heated to 70 °C. For compounds **6a**,**f** and **7a**,**f**, the reactions were run at room temperature. The reaction was monitored via TCL (9:1 hexanes/CH_2Cl_2) and when complete (typically 2–4 h), the mixture was immediately worked up according to the following procedures.

***anti*-IDBT 6a:** Following general procedure C, dione **10** (200 mg, 0.50 mmol) and mesitylmagnesium bromide (5.0 mmol) were reacted to yield diol **S9**. Following general procedure D, diol **S9** and $SnCl_2$ (379 mg, 2.0 mmol) were reacted. Upon completion, the mixture was filtered through a silica gel plug (CH_2Cl_2). The filtrate was concentrated *in vacuo* and the residue purified via flash chromatography (6:1 hexanes/CH_2Cl_2) to yield **6a** (110 mg, 36%) as a deep purple solid. Spectroscopic data matched those previously reported.[1]

***anti*-IDBT 6b:** Following general procedure B, dione **10** (150 mg, 0.38 mmol) and aryl lithiate (2.09 mmol) were reacted to yield diol **S1**. Following general procedure D, diol **S1** and $SnCl_2$, (288.2 mg, 1.52 mmol) were reacted. Upon completion, the mixture was cooled in an ice bath and the solid was collected via vacuum filtration. The product was washed with acetone and EtOH to remove residual $SnCl_2$, and then further washed with hexanes and CH_2Cl_2. The iridescent deep purple powder (**6b**, 182 mg, 76%)) was collected and dried overnight under a high vacuum. ^1H NMR (600 MHz, $CDCl_2CDCl_2$, 70 °C) δ 7.58 (d, J = 7.7 Hz, 4H), 7.54 (d, J = 8.0 Hz, 2H), 7.48 (d, J = 7.4 Hz, 2H), 7.28 (t, J = 7.4 Hz, 2H), 7.24−7.18 (m, 2H), 7.18−7.10 (m,

4H), 6.64 (s, 2H), 1.53 (s, 18H). Due to poor solubility, ^{13}C NMR data could not be obtained. HRMS (TOF MS ES+) for $C_{44}H_{37}S_2$ $(M+H)^+$: calcd 629.2337, found 629.2360.

***anti*-IDBT 6c:** Following general procedure B, dione **10** (150 mg, 0.38 mmol) and aryl lithiate (2.09 mmol) were reacted to yield diol **S2**. Following general procedure D, diol **S2** and $SnCl_2$, (288.2 mg, 1.52 mmol) were reacted. Upon completion, the mixture was cooled in an ice bath and the solid was collected via vacuum filtration. The product was washed with acetone and EtOH to remove residual $SnCl_2$, and then further washed with hexanes and CH_2Cl_2. The iridescent deep purple powder (**6c**, 53.5 mg, 27%) was collected and dried overnight under a high vacuum. ^{1}H NMR (500 MHz, $CDCl_2CDCl_2$, 70 °C) δ 7.67−7.54 (m, 6H), 7.54−7.48 (m, 2H), 7.37 (s, 2H), 7.28 (t, J = 7.7 Hz, 2H), 7.24−7.17 (m, 2H), 7.12 (s, 4H), 6.56 (s, 2H). Due to poor solubility, ^{13}C NMR data could not be obtained. HRMS (TOF MS ES+) for $C_{36}H_{21}S_2$ $(M+H)^+$: calcd 517.1085, found 517.1093.

***anti*-IDBT 6d:** Following general procedure C, dione **10** (800 mg, 2.03 mmol) and pentafluorophenylmagnesium bromide (20.3 mmol) were reacted to yield diol **S10**. Following general procedure D, diol **S10**, $SnCl_2$, (1.539 g, 8.11 mmol) and TFA (1.6 mL) were reacted. Upon completion, the mixture was cooled in an ice bath and the solid was collected via vacuum filtration. The product was washed with CH_3CN and EtOH to remove residual $SnCl_2$, and then further washed with hexanes and acetone. The dark blue powder (**6d**, 824 mg, 59%) was collected and dried overnight under a high vacuum. ^{1}H NMR (500 MHz, $CDCl_2CDCl_2$, 100 °C) δ 7.53−7.47 (m, 2H), 7.17−7.09 (m, 4H), 6.87−6.82 (m, 2H), 5.93 (s, 2H). ^{19}F NMR (471 MHz, $CDCl_2CDCl_2$, 100 °C) δ −136.54 (dd, J = 23.9, 7.9 Hz), −152.63 (t, J = 20.6 Hz), −160.99 (td, J = 22.4, 22.0, 7.9 Hz). Due to poor solubility, ^{13}C NMR data could not be obtained. HRMS (TOF MS ES+) for $C_{36}H_{10}F_{10}S_2$ $(M+H)^+$: calcd 696.0064, found 696.0026.

***anti*-IDBT 6e:** Following general procedure B, dione **10** (150 mg, 0.38 mmol) and aryl lithiate (2.09 mmol) were reacted to yield diol **S3**. Following general procedure D, diol **S3**, $SnCl_2$ (288 mg, 1.52 mmol) and TFA (0.3 mL) were reacted. Upon completion, the mixture was cooled in an ice bath and the solid was collected via vacuum filtration. The product was washed with acetone and EtOH to remove residual $SnCl_2$, and then further washed with hexanes and CH_2Cl_2. The bright blue powder (**6e**, 220 mg, 74%) was collected and dried overnight under a high vacuum. ^{1}H NMR (500 MHz, $CDCl_2CDCl_2$, 100 °C) δ 8.02 (s, 6H), 7.56 (d, J = 7.5 Hz, 2H), 7.19−7.12 (m, 4H), 7.09 (d, J = 7.1 Hz, 2H), 6.28 (s, 2H). ^{19}F NMR (471 MHz, $CDCl_2CDCl_2$,

100 °C) δ −64.19. Due to poor solubility, ^{13}C NMR data could not be obtained. HRMS (TOF MS ES+) for $C_{40}H_{17}F_{12}S_2$ $(M+H)^+$: calcd 789.0580, found 789.0610.

***anti*-IDBT 6f:** Following general procedure B, dione **10** (197 mg, 0.50 mmol) and (triisopropylsilyl)ethynyl lithiate (2.75 mmol) were reacted to yield diol **S4**. Following general procedure D, diol **S4** and $SnCl_2$, (375.0 mg, 2.0 mmol) were reacted. Upon completion, the mixture was filtered through a silica gel plug (CH_2Cl_2). The filtrate was concentrated *in vacuo* and purified via flash chromatography (6:1 hexanes/CH_2Cl_2) to yield **6f** (215 mg, 59%) as a dark green solid. ^{1}H NMR (600 MHz, $CDCl_3$) δ 7.81 (d, J = 7.5 Hz, 2H), 7.48 (d, J = 7.5 Hz, 2H), 7.18 (d, J = 7.5 Hz, 2H), 7.10 (d, J = 7.5 Hz, 2H), 6.38 (s, 2H), 1.25−1.16 (m, 42H). ^{13}C NMR (151 MHz, $CDCl_3$) δ 150.34, 143.77, 143.17, 142.43, 132.76, 131.27, 126.66, 125.81, 124.82, 124.47, 123.83, 121.59, 112.85, 102.92, 18.91, 11.58. HRMS (TOF MS ES+) for $C_{46}H_{53}Si_2S_2$ $(M+H)^+$: calcd 725.3127, found 725.3105.

***syn*-IDBT 7a:** Following general procedure B, dione **11** (200 mg, 0.50 mmol) and mesitylmagnesium bromide (5.0 mmol) were reacted to yield diol **S11**. Following general procedure D, diol **S11** and $SnCl_2$ (379 mg, 2.0 mmol) were reacted. Upon completion, the mixture was filtered through a silica gel plug (CH_2Cl_2). The filtrate was concentrated *in vacuo* and purified via flash chromatography (6:1 hexanes/CH_2Cl_2) to yield **7a** (140 mg, 47%) as a deep purple solid. Spectroscopic data matched those previously reported.[1]

***syn*-IDBT 7b:** Following general procedure B, dione **11** (150 mg, 0.38 mmol) and aryl lithiate (2.09 mmol) were reacted to yield diol **S5**. Following general procedure D, diol **S5** and $SnCl_2$ (288 mg, 1.52 mmol) were reacted. Upon completion, the mixture was cooled in an ice bath and the solid was collected via vacuum filtration. The product was washed with acetone and EtOH to remove residual $SnCl_2$, and then further washed with hexanes and CH_2Cl_2. The iridescent deep purple powder (**7b**, 116.4 mg, 49%) was collected and dried overnight under a high vacuum. ^{1}H NMR (600 MHz, CD_2Cl_2) δ 7.63−7.52 (m, 12H), 7.24 (t, J = 7.3 Hz, 2H), 7.15 (t, J = 7.5 Hz, 2H), 6.93 (s, 2H), 1.40 (s, 18H). ^{13}C NMR (151 MHz, CD_2Cl_2) δ 153.66, 147.16, 146.36, 144.58, 142.82, 135.58, 135.08, 133.61, 131.49, 128.73, 126.81, 125.97, 125.67, 125.43, 124.48, 122.60, 35.46, 31.50. HRMS (TOF MS ES+) for $C_{44}H_{37}S_2$ $(M+H)^+$: calcd 629.2337, found 629.2358.

***syn*-IDBT 7c:** Following general procedure B, dione **11** (150 mg, 0.38 mmol) and aryl lithiate (2.09 mmol) were reacted to yield diol **S6**. Following general procedure D, diol **S6** and

$SnCl_2$ (288 mg, 1.52 mmol) were reacted. Upon completion, the mixture was cooled in an ice bath and the solid was collected via vacuum filtration. The product was washed with acetone and EtOH to remove residual $SnCl_2$, and then further washed with hexanes and CH_2Cl_2. The iridescent deep purple powder (**7c**, 130.5 mg, 67%) was collected and dried overnight under a high vacuum. ^{1}H NMR (600 MHz, $CDCl_2CDCl_2$, 70 °C) δ 7.63 (d, J = 6.7 Hz, 4H), 7.62−7.46 (m, 10H), 7.25 (t, J = 7.7 Hz, 2H), 7.17 (t, J = 7.3 Hz, 2H), 6.85 (s, 2H). Due to poor solubility, ^{13}C NMR data could not be obtained. HRMS (TOF MS ES+) for $C_{36}H_{21}S_2$ $(M+H)^+$: calcd 517.1085, found 517.1068.

***syn*-IDBT 7d:** Following general procedure C, dione **11** (150 mg, 0.38 mmol) and pentafluorophenylmagnesium bromide (3.80 mmol) were reacted to yield diol **S12**. Following general procedure D, diol **S12**, $SnCl_2$ (288 mg, 1.52 mmol) and TFA (0.3 mL) were reacted. Upon completion, the mixture was cooled in an ice bath and the solid was collected via vacuum filtration. The product was washed with CH_3CN and EtOH to remove residual $SnCl_2$, and then further washed with hexanes and acetone. The dark blue powder (**7d**, 189.5 mg, 72%) was collected and dried overnight under a high vacuum. ^{1}H NMR (600 MHz, C_6D_6) δ 7.21 (d, J = 8.0 Hz, 2H), 7.02 (d, J = 8.3 Hz, 2H), 6.75 (d, J = 7.5 Hz, 2H), 6.69 (d, J = 7.5 Hz, 2H), 5.93 (d, J = 2.4 Hz, 2H). It should be noted that the doublet at 7.02 ppm is overlaid over the ^{13}C satellite peak and as such the integration reads artificially high. The other ^{13}C satellite peak has been intergrated to show the magnitude of this calculation error. ^{19}F NMR (282 MHz, C_4D_8O) δ –135.10 (d, J = 22.5 Hz), –153.12 (t, J = 20.8 Hz), –161.03 (t, J = 18.6 Hz). Due to poor solubility, ^{13}C NMR data could not be obtained. HRMS (TOF MS ES+) for $C_{36}H_{10}F_{10}S_2$ $(M+H)^+$: calcd 697.0143, found 697.0146.

***syn*-IDBT 7e:** Following general procedure B, dione **11** (150 mg, 0.38 mmol) and aryl lithiate (2.09 mmol) were reacted to yield diol **S7**. Following general procedure D, diol **S7**, $SnCl_2$ (288 mg, 1.52 mmol) and TFA (0.3 mL) were reacted. Upon completion, the mixture was cooled in an ice bath and the solid was collected via vacuum filtration. The product was washed with acetone and EtOH to remove residual $SnCl_2$, and then further washed with hexanes and CH_2Cl_2. The bright blue powder (**7e**, 206 mg, 69%) was collected and dried overnight under a high vacuum. ^{1}H NMR (500 MHz, $CDCl_2CDCl_2$, 100 °C) δ 8.00 (s, 4H), 7.97 (s, 2H), 7.58 (d, J = 8.0 Hz, 2H), 7.39 (d, J = 8.0 Hz, 2H), 7.26 (t, J = 7.2 Hz, 2H), 7.21 (d, J = 7.3 Hz, 2H), 6.54 (s, 2H). ^{19}F NMR (471 MHz, $CDCl_2CDCl_2$, 100 °C) δ −64.37. Due to poor solubility, ^{13}C NMR data

could not be obtained. HRMS (TOF MS ES+) for $C_{40}H_{17}F_{12}S_2$ $(M+H)^+$: calcd 789.0580, found 789.0590.

***syn*-IDBT 7f:** Following general procedure B, dione **11** (150 mg, 0.38 mmol) and (triisopropylsilyl)ethynyl lithiate (2.75 mmol) were reacted to yield diol **S8**. Following general procedure D, diol **S8** and $SnCl_2$ (288 mg, 1.52 mmol) were reacted. Upon completion, the mixture was filtered through a silica gel plug (CH_2Cl_2). The filtrate was concentrated *in vacuo* and purified via flash chromatography (6:1 hexanes/CH_2Cl_2) to yield **7f** (3.5 mg, 1.4%) as a dark green solid. ^{1}H NMR (600 MHz, $CDCl_3$) δ 7.49 (d, *J* = 8.1 Hz, 2H), 7.37 (d, *J* = 8.0 Hz, 2H), 7.17 (d, *J* = 7.6 Hz, 2H), 7.10 (d, *J* = 7.6 Hz, 2H), 6.39 (s, 2H), 1.25–1.11 (m, 42H). Due to rapid degradation of the compound, ^{13}C NMR data was unable to be collected. HRMS (TOF MS ES+) for $C_{46}H_{53}Si_2S_2$ $(M+H)^+$: calcd 725.3127, found 725.3152.

Cyclic Voltammetry (CV)

General. All electrochemical experiments were conducted with traditional 3-electrode geometry using a Biologic SP-50 potentiostat. Electrolyte solutions (0.1 M) were prepared from HPLC grade CH_2Cl_2 or THF and anhydrous Bu_4NBF_4 or Bu_4NOTf and the stock solutions were degassed via freeze-pump-thaw (3x) cycles. The working electrode was a glassy carbon electrode (3-mm diameter), with a Pt-coil counter electrode and a Ag wire pseudo reference. The ferrocene/ferrocenium (Fc/Fc^+) couple was used as an internal standard following each experiment. Potential values were re-referenced to SCE using a value of 0.46 (V vs. SCE) for the Fc/Fc^+ couple in CH_2Cl_2 and 0.56 (V vs. SCE) for the Fc/Fc^+ couple in THF. When necessary, potentials were re-referenced to NHE using SCE = –0.24 (V vs. NHE). LUMO and HOMO levels were approximated using SCE = –4.68 eV vs. vacuum.[4] CV experiments were conducted in a N_2-filled drybox at sweep rates of 50 (reported), 75, 100 and 125 mV s^{-1}. All scan rates show quasi-reversible kinetics with no alteration of peak splitting with scan rate. $E_{1/2}$ values were calculated assuming $E_{1/2} \approx E^{o\prime}=(E_{anodic} + E_{cathodic})/2$ based on these observations for reversible couples; for irreversible couples the $E^{o\prime}$ value is estimated as the potential at peak current. The $E_{a,c}$ peak splitting of the Fc/Fc^+ couple was similar to that of the analyte (~100 mV). The anodic peak current increased linearly with the square root of the scan rate in the range of 50–125 mV s^{-1}, indicating a diffusion-controlled process. Analyte concentrations were ca. 1-5 mM.

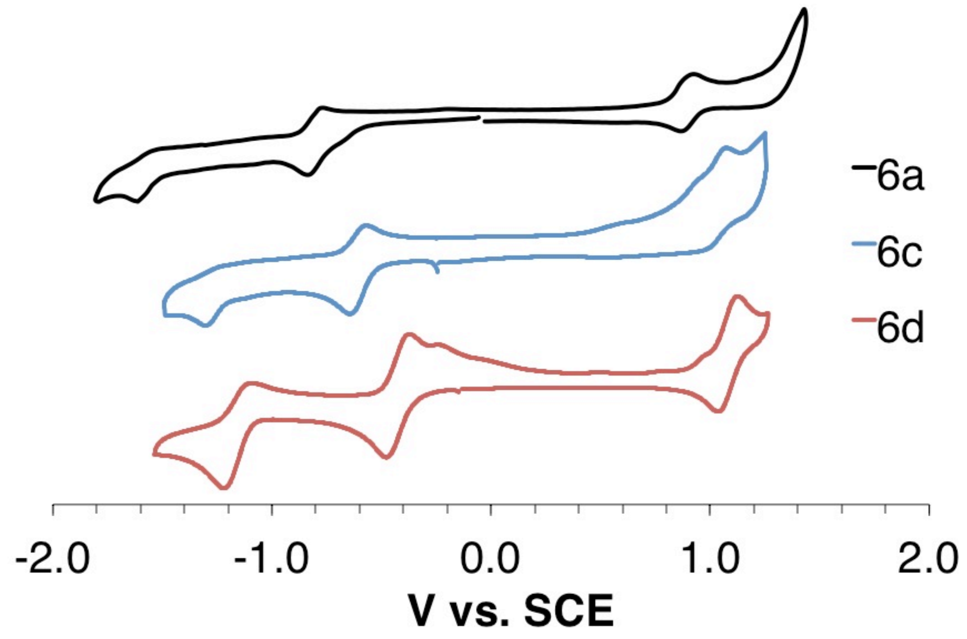

Figure S1. CV data of *anti*-IDBTs **6a**, **6c**, and **6d**.

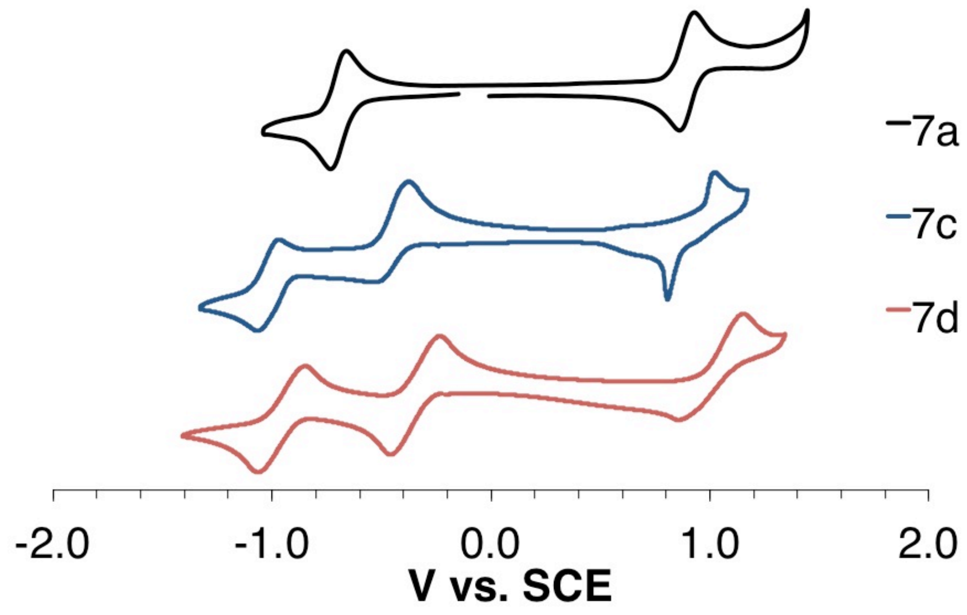

Figure S2. CV data of *syn*-IDBTs **7a**, **7c**, and **7d**.

X-ray Crystallography

General. Diffraction intensities for **6d**,**f**, and **7b**,**d** were collected at 150 K (**6f** and **7b**) and 223 K (**6d** and **7d**) on a Bruker Apex2 CCD diffractometer using MoKα (**6f**) and CuKα radiation, λ= 0.71073 Å and 1.54178 Å, respectively. Space groups were determined based on systematic absences (**6d** and **7b**) and intensity statistics (**6f** and **7d**). Absorption corrections were applied by SADABS.[5] Structures were solved by direct methods and Fourier techniques and refined on F^2 using full matrix least-squares procedures. All non-H atoms were refined with anisotropic thermal parameters. H atoms in **6d** and **7d** were found from the residual density map and refined with isotropic thermal parameter. H atoms in **6f** and **7b** were refined in calculated positions in a rigid group model. The solvent molecule $CHCl_3$ in **7b** is disordered over two positions with the same positions for the Cl atoms and opposite orientations for the CH group. The disordered C atom in this solvent molecule was refined with isotropic thermal parameters. Crystals of **7b** were very thin plates and intensities at the high angles for all crystals were very weak even with using a strong Incoatec I*μS* Cu source; thus, only reflections up to $2\theta_{max} = 107.8°$ were collected and used in the refinement. All calculations were performed by the Bruker SHELXL-2013 package.[6]

Crystallographic Data for **6d**: $C_{36}H_{10}F_{10}S_2$, M = 696.56, 0.14 × 0.10 × 0.02 mm, T = 223 K, Orthorhombic, space group *Pbca*, a = 9.5254(3) Å, b = 12.2658(5) Å, c = 24.0109(7) Å, V = 2805.35(17) Å^3, Z = 4, D_c = 1.649 Mg/m^3, μ(Cu) = 2.590 mm^{-1}, F(000) = 1392, $2\theta_{max}$ = 133.2°, 12847 reflections, 2481 independent reflections [R_{int} = 0.0300], R1 = 0.0385, wR2 = 0.1152 and GOF = 1.030 for 2481 reflections (237 parameters) with I>2σ(I), R1 = 0.0480, wR2 = 0.1244 and GOF = 1.030 for all reflections, max/min residual electron density +0.245/–0.231 eÅ^3. CCDC 1451107.

Crystallographic Data for **6f**: $C_{46}H_{52}S_2Si_2$, M = 725.17, 0.18 × 0.04 × 0.03 mm, T = 150 K, Triclinic, space group *P*-1, a = 8.041(3) Å, b = 11.320(4) Å, c = 12.549(5) Å, α = 106.204(11)°, β = 106.976(9)°, γ = 99.176(11)°, V = 1012.3(7) Å^3, Z = 1, D_c = 1.190 Mg/m^3, μ(Mo) = 0.222 mm^{-1}, F(000) = 388, $2\theta_{max}$ = 50.0°, 13155 reflections, 3395 independent reflections [R_{int} = 0.1610], R1 = 0.0667, wR2 = 0.1233 and GOF = 0.985 for 3395 reflections (226 parameters) with I>2σ(I), R1 = 0.1488, wR2 = 0.1460 and GOF = 0.985 for all reflections, max/min residual electron density +0.395/–0.352 eÅ^3. CCDC 1451110.

Crystallographic Data for **7b**: $C_{44.5}H_{36.5}S_2Cl_{1.5}$, $C_{44}H_{36}S_2$•0.5($CHCl_3$), M = 688.53, 0.09 × 0.05 × 0.01 mm, T = 150 K, Monoclinic, space group $P2_1/c$, a = 13.7863(11) Å, b = 37.373(3) Å, c = 7.0980(6) Å, β = 100.313(5)°, V = 3598.1(5) $Å^3$, Z = 4, D_c = 1.271 Mg/m^3, μ(Cu) = 2.593 mm^{-1}, F(000) = 1444, $2\theta_{max}$ = 107.8°, 13782 reflections, 4023 independent reflections [R_{int} = 0.0825], R1 = 0.0737, wR2 = 0.1903 and GOF = 1.031 for 4023 reflections (450 parameters) with I>2σ(I), R1 = 0.1126, wR2 = 0.2203 and GOF = 1.031 for all reflections, max/min residual electron density +0.713/–0.376 e$Å^3$. CCDC 1451109.

Crystallographic Data for **7d**: $C_{36}H_{10}F_{10}S_2$, M = 696.56, 0.12 × 0.08 × 0.04 mm, T = 223 K, Triclinic, space group P-1, a = 11.1062(5) Å, b = 14.1291(6) Å, c = 14.3389(6) Å, α = 70.677(2)°, β = 76.199(3)°, γ = 86.760(3)°, V = 2061.40(16) $Å^3$, Z = 3, D_c = 1.683 Mg/m^3, μ(Cu) = 2.643 mm^{-1}, F(000) = 1044, $2\theta_{max}$ = 133.2°, 27258 reflections, 7106 independent reflections [R_{int} = 0.0444], R1 = 0.0467, wR2 = 0.1170 and GOF = 1.044 for 7106 reflections (709 parameters) with I>2σ(I), R1 = 0.0579, wR2 = 0.1253 and GOF = 1.044 for all reflections, max/min residual electron density +0.315/–0.270 e$Å^3$. CCDC 1451108.

NICS-XY Scan Computations

General. All calculations related to the NICS-XY scans were performed using Gaussian 09.[7] All geometries were optimized using RB3LYP/6-31++G(d,p) level of theory and the structures were verified to be real minima via analytical frequency calculations to ensure no imaginary frequencies, A closed shell ground state was confirmed by running a stability check on the final geometry. All NICS-XY scans were carried out using the Aroma package described by Stanger and co-workers[8-11] at the B3LYP/6-311+G* level of theory following established procedures.[8-10]

Comparison of $NICS_{zz}$ and $NICS_{\pi ZZ}$

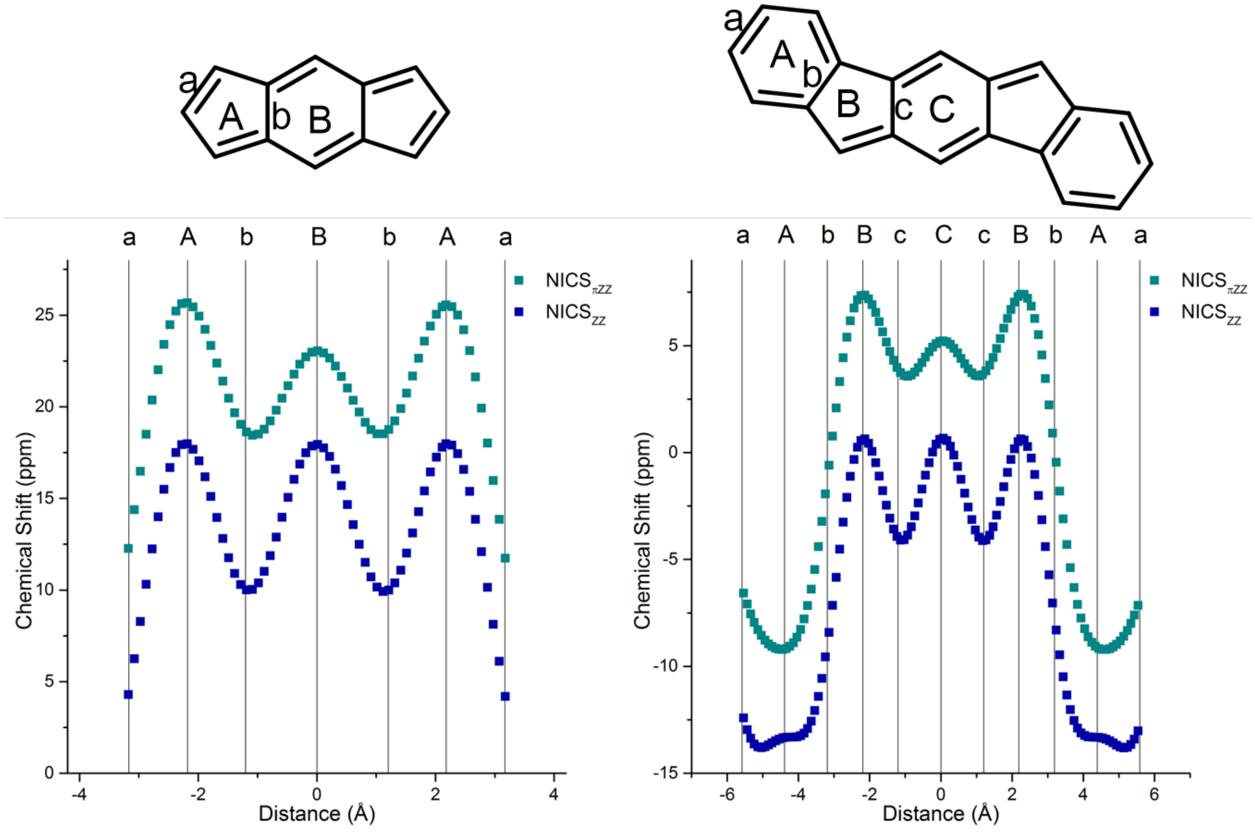

Figure S3. Compairson of $NICS_{ZZ}$ and $NICS_{\pi ZZ}$ for *s*-indacene (left) and indeno[1,2-*b*]fluorene **1`** (right).

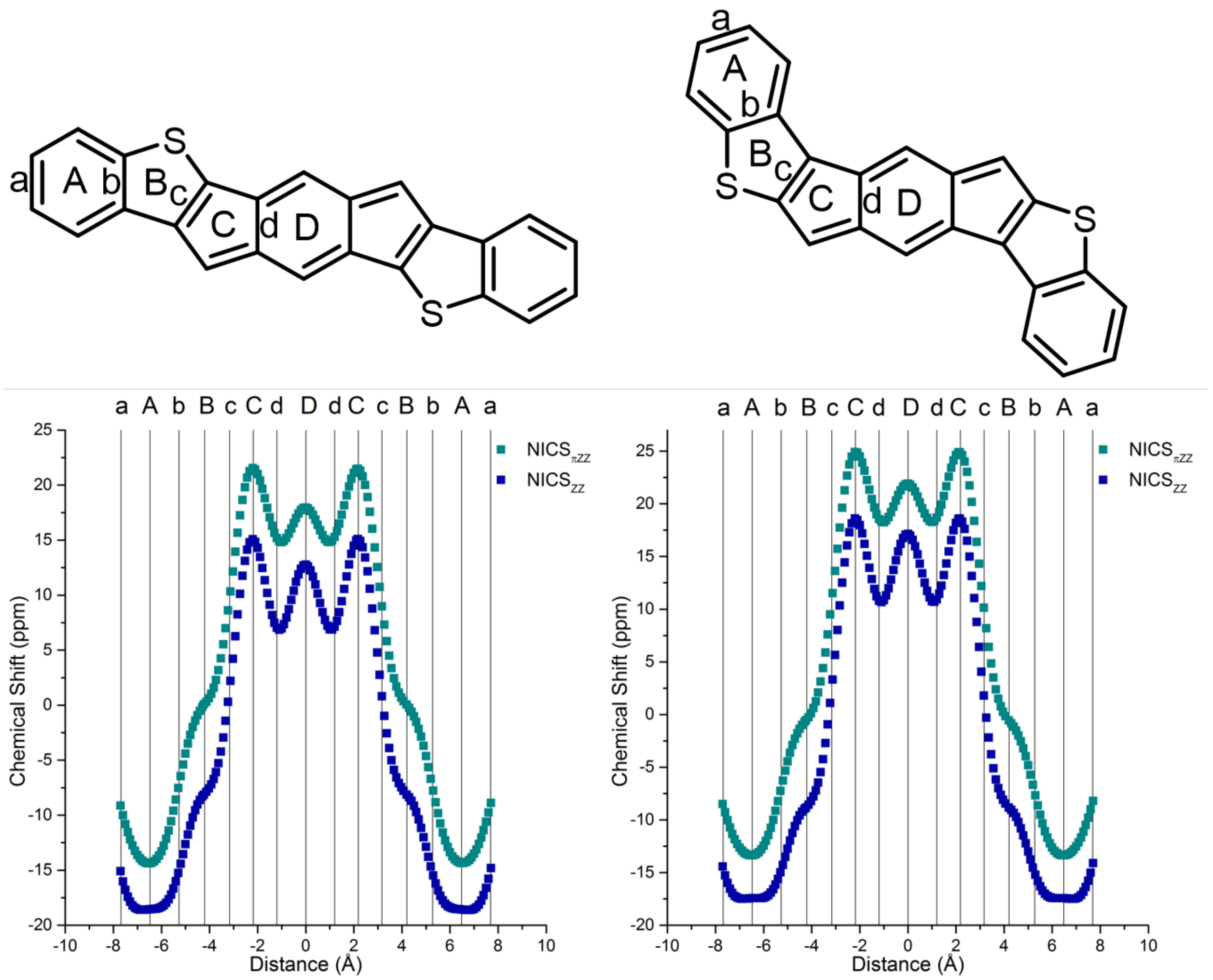

Figure S4. Comparison of $NICS_{ZZ}$ and $NICS_{\pi ZZ}$ for *anti*-IDBT **6`** (left) and *syn*-IDBT **7`** (right).

Cartesian Coordinates and Energy Data For NICS-XY Geometries

s-Indacene

RB3LYP/6-31++G(d,p)

Zero-point correction =	0.156060 (Hartree/Particle)
Thermal correction to Energy =	0.164354
Thermal correction to Enthalpy =	0.165298
Thermal correction to Gibbs Free Energy =	0.123646
Sum of electronic and zero-point Energies =	-461.902145
Sum of electronic and thermal Energies =	-461.893851
Sum of electronic and thermal Enthalpies =	-461.892907
Sum of electronic and thermal Free Energies =	-461.934558

Symbol	X	Y	Z
C	-1.21028	0.72205	0.00000
C	-1.19519	-0.72871	0.00000
C	0.02912	-1.44385	0.00000
C	1.21028	-0.72205	0.00000
C	-0.02912	1.44385	0.00000
C	1.19519	0.72871	0.00000
C	2.59552	-1.12893	0.00000
C	2.5323	1.15841	0.00000
C	3.38635	0.01159	0.00000
H	0.03211	-2.53229	0.00000
H	2.94882	-2.15363	0.00000
H	4.46861	0.03755	0.00000
C	-2.59552	1.12893	0.00000
C	-2.5323	-1.15841	0.00000
C	-3.38635	-0.01159	0.00000
H	2.86686	2.19016	0.00000
H	-0.03211	2.53229	0.00000
H	-2.86686	-2.19016	0.00000
H	-4.46861	-0.03755	0.00000
H	-2.94882	2.15363	0.00000

Indeno[1,2-*b*]fluorene (compound **1`**)

RB3LYP/6-31++G(d,p)

Zero-point correction =	0.251575 (Hartree/Particle)
Thermal correction to Energy =	0.264910
Thermal correction to Enthalpy =	0.265854
Thermal correction to Gibbs Free Energy =	0.212308
Sum of electronic and zero-point Energies =	-769.141567
Sum of electronic and thermal Energies =	-769.128233
Sum of electronic and thermal Enthalpies =	-769.127288
Sum of electronic and thermal Free Energies =	-769.180834

Symbol	X	Y	Z
H	-2.43941	3.46846	0.00000
H	2.34832	4.94205	0.00000
H	2.43941	-3.46846	0.00000
H	-2.34832	-4.94205	0.00000
H	1.85782	-5.8825	0.00000
H	-0.50516	-6.61454	0.00000
H	0.50516	6.61454	0.00000
H	-1.85782	5.8825	0.00000
H	2.42643	-0.74692	0.00000
H	-2.42643	0.74692	0.00000
C	0.37194	-1.36626	0.00000
C	1.38063	-0.44883	0.00000
C	1.03666	0.94686	0.00000
C	-0.37194	1.36626	0.00000
C	-1.38063	0.44883	0.00000
C	-1.03666	-0.94686	0.00000
C	1.83487	2.07039	0.00000
C	0.99155	3.25502	0.00000
C	-0.37194	2.83469	0.00000
C	0.37194	-2.83469	0.00000
C	-1.83487	-2.07039	0.00000
C	-0.99155	-3.25502	0.00000
C	-1.39683	3.77515	0.00000
C	1.31193	4.61521	0.00000
C	1.39683	-3.77515	0.00000
C	-1.31193	-4.61521	0.00000
C	1.06593	-5.13921	0.00000
C	-0.27243	-5.55371	0.00000
C	0.27243	5.55371	0.00000
C	-1.06593	5.13921	0.00000
H	2.91968	2.08058	0.00000
H	-2.91968	-2.08058	0.00000

Anti-IDBT (compound **6`**)

RB3LYP/6-31++G(d,p)

Zero-point correction = 0.277989 (Hartree/Particle)
Thermal correction to Energy = 0.296369
Thermal correction to Enthalpy = 0.297313
Thermal correction to Gibbs Free Energy = 0.231248
Sum of electronic and zero-point Energies = -1717.916565
Sum of electronic and thermal Energies = -1717.898185
Sum of electronic and thermal Enthalpies = -1717.897241
Sum of electronic and thermal Free Energies = -1717.963306

Symbol	X	Y	Z
C	1.31507	0.50226	-0.00002
C	1.04960	-0.93617	0.00000
C	-0.28120	-1.41906	0.00001
C	-1.31507	-0.50226	-0.00001
C	-1.04960	0.93617	-0.00004
C	0.28120	1.41906	-0.00004
C	2.74903	0.62133	-0.00002
C	3.32863	-0.66125	0.00000
C	-2.74903	-0.62133	0.00000
C	-3.32863	0.66125	-0.00001
C	2.27656	-1.62189	0.00000
S	3.91940	1.90023	-0.00004
C	5.24619	0.71193	-0.00003
C	4.77055	-0.62749	-0.00002
C	-2.27656	1.62189	-0.00004
S	-3.91940	-1.90023	0.00004
C	-5.24619	-0.71193	0.00005
C	-4.77055	0.62749	0.00002
C	6.60886	1.01065	-0.00005
C	7.52127	-0.04584	-0.00004
C	-6.60886	-1.01065	0.00008
C	-7.52127	0.04584	0.00008
C	7.07242	-1.37702	-0.00002
C	5.71118	-1.67172	-0.00001
C	-7.07242	1.37702	0.00005
C	-5.71118	1.67172	0.00002
H	6.95474	2.03995	-0.00006
H	8.58600	0.16638	-0.00005
H	-6.95474	-2.03995	0.00010
H	-8.58600	-0.16638	0.00010
H	7.79637	-2.18645	-0.00001
H	5.37379	-2.70422	0.00001
H	-7.79637	2.18645	0.00005
H	-5.37379	2.70422	0.00000
H	2.39568	-2.69955	0.00002
H	0.47331	2.48992	-0.00006
H	-0.47331	-2.48992	0.00003
H	-2.39568	2.69955	-0.00006

Syn-IDBT (compound **7`**)

RB3LYP/6-31++G(d,p)

Zero-point correction = 0.278306 (Hartree/Particle)
Thermal correction to Energy = 0.296665
Thermal correction to Enthalpy = 0.297609
Thermal correction to Gibbs Free Energy = 0.231563
Sum of electronic and zero-point Energies = -1717.914495
Sum of electronic and thermal Energies = -1717.896137
Sum of electronic and thermal Enthalpies = -1717.895192
Sum of electronic and thermal Free Energies = -1717.961238

Symbol	X	Y	Z
C	-1.41574	-0.09840	-0.00004
C	-0.72999	1.19276	-0.00005
C	0.69513	1.26754	-0.00003
C	1.41574	0.0984	-0.00001
C	0.72999	-1.19276	0.00001
C	-0.69513	-1.26754	-0.00001
C	2.84507	-0.19914	0.00003
C	2.97160	-1.59054	0.00005
C	1.68611	-2.21203	0.00003
C	-2.84507	0.19914	-0.00005
C	-2.97160	1.59054	-0.00007
C	-1.68611	2.21203	-0.00008
C	-4.10615	-0.47679	-0.00004
S	-4.63110	2.13843	-0.00008
C	-5.18568	0.45931	-0.00004
C	4.10615	0.47679	0.00004
S	4.63110	-2.13843	0.00007
C	5.18569	-0.45931	0.00007
C	-4.41329	-1.85424	-0.00001
C	-5.73879	-2.26785	0.00000
C	-6.78787	-1.32808	-0.00001
C	-6.51833	0.03887	-0.00003
C	4.41329	1.85424	0.00003
C	5.73879	2.26785	0.00005
C	6.78787	1.32808	0.00008
C	6.51833	-0.03887	0.00009
H	-3.61329	-2.58758	-0.00001
H	-5.97090	-3.32865	0.00002
H	-7.81824	-1.67042	0.00000
H	-7.32848	0.76177	-0.00004
H	3.61329	2.58758	0.00001
H	5.97090	3.32865	0.00004
H	7.81824	1.67042	0.00009
H	7.32848	-0.76177	0.00011
H	1.48591	-3.27695	0.00004
H	-1.18104	-2.23996	0.00001
H	1.18104	2.23996	-0.00004
H	-1.48591	3.27695	-0.00010

***Anti*-IDN** (compound **8`**)

RB3LYP/6-31++G(d,p)

Zero-point correction=	0.344851 (Hartree/Particle)
Thermal correction to Energy=	0.363751
Thermal correction to Enthalpy=	0.364695
Thermal correction to Gibbs Free Energy=	0.297491
Sum of electronic and zero-point Energies=	-1076.343768
Sum of electronic and thermal Energies=	-1076.324868
Sum of electronic and thermal Enthalpies=	-1076.323924
Sum of electronic and thermal Free Energies=	-1076.391128

C	-0.6929700000	0.6503900000	1.0866200000
C	0.6162900000	1.0534700000	0.6643400000
C	1.3130500000	0.3859400000	-0.4421200000
C	2.6153000000	1.0596000000	-0.5883500000
C	2.6606200000	2.0733000000	0.3980100000
C	1.4277100000	2.0543600000	1.1549500000
C	3.7830800000	2.9199700000	0.5409100000
C	4.8587200000	2.7516300000	-0.3015100000
C	4.8583500000	1.7444300000	-1.3099600000
C	3.7184200000	0.8727100000	-1.4694600000
C	3.7742800000	-0.1135300000	-2.4956900000
C	4.8772800000	-0.2392300000	-3.3132300000
C	5.9935700000	0.6169100000	-3.1526500000
C	5.9770500000	1.5844400000	-2.1714800000
H	-1.1501800000	1.1871900000	1.9105600000
H	3.7923900000	3.6909800000	1.3063700000
H	5.7328000000	3.3907000000	-0.2100800000
H	2.9353900000	-0.7811800000	-2.6416200000
H	4.8900500000	-1.0012800000	-4.0871800000
H	6.8572000000	0.5093400000	-3.8021300000
H	6.8290900000	2.2469400000	-2.0414600000
C	0.6929700000	-0.6503900000	-1.0866200000
C	-0.6162900000	-1.0534700000	-0.6643400000
C	-1.3130500000	-0.3859400000	0.4421200000
C	-2.6153000000	-1.0596000000	0.5883500000
C	-2.6606200000	-2.0733000000	-0.3980100000
C	-1.4277100000	-2.0543600000	-1.1549500000
C	-3.7830800000	-2.9199700000	-0.5409100000
C	-4.8587200000	-2.7516300000	0.3015100000
C	-4.8583500000	-1.7444300000	1.3099600000
C	-3.7184200000	-0.8727100000	1.4694600000
C	-3.7742800000	0.1135300000	2.4956900000
C	-4.8772800000	0.2392300000	3.3132300000
C	-5.9935700000	-0.6169100000	3.1526500000
C	-5.9770500000	-1.5844400000	2.1714800000
H	1.1501800000	-1.1871900000	-1.9105600000
H	-3.7923900000	-3.6909800000	-1.3063700000
H	-5.7328000000	-3.3907000000	0.2100800000
H	-2.9353900000	0.7811800000	2.6416200000
H	-4.8900500000	1.0012800000	4.0871800000
H	-6.8572000000	-0.5093400000	3.8021300000
H	-6.8290900000	-2.2469400000	2.0414600000
H	1.1917200000	2.7247600000	1.9748300000
H	-1.1917200000	-2.7247600000	-1.9748300000

***Syn*-IDN** (compound **9`**)

RB3LYP/6-31++G(d,p)

Zero-point correction= 0.344756 (Hartree/Particle)
Thermal correction to Energy= 0.363570
Thermal correction to Enthalpy= 0.364515
Thermal correction to Gibbs Free Energy= 0.297860
Sum of electronic and zero-point Energies= -1076.350386
Sum of electronic and thermal Energies= -1076.331572
Sum of electronic and thermal Enthalpies= -1076.330628
Sum of electronic and thermal Free Energies= -1076.397283

C	0.1728070000	1.4393410000	0.0000030000
C	-1.1254300000	0.8332770000	0.0000010000
C	-1.2698990000	-0.6225270000	-0.0000030000
C	-2.7079060000	-0.8993550000	-0.0000010000
C	-3.3811590000	0.3437990000	0.0000000000
C	-2.3898250000	1.3980120000	0.0000010000
C	-3.4255830000	-2.1092820000	-0.0000010000
H	-2.9050860000	-3.0629900000	-0.0000010000
C	-4.8080790000	-2.0725090000	-0.0000010000
H	-5.3753430000	-2.9995600000	-0.0000010000
C	-5.5252240000	-0.8406380000	-0.0000010000
C	-4.8058910000	0.4050660000	-0.0000010000
C	-5.5425310000	1.6188630000	-0.0000010000
H	-5.0087860000	2.5645380000	-0.0000010000
C	-6.9226930000	1.6109840000	-0.0000010000
H	-7.4693450000	2.5496030000	-0.0000010000
C	-7.6331420000	0.3856230000	-0.0000010000
H	-8.7191090000	0.3914710000	-0.0000010000
C	-6.9463470000	-0.8102300000	-0.0000010000
H	-7.4879320000	-1.7528510000	-0.0000010000
C	-0.1728070000	-1.4393410000	-0.0000060000
C	1.1254300000	-0.8332770000	-0.0000010000
C	1.2698990000	0.6225270000	0.0000020000
C	2.7079060000	0.8993550000	0.0000040000
C	3.3811590000	-0.3437990000	0.0000010000
C	2.3898250000	-1.3980120000	0.0000030000
C	3.4255830000	2.1092820000	0.0000050000
H	2.9050860000	3.0629900000	0.0000070000
C	4.8080790000	2.0725090000	0.0000050000
H	5.3753430000	2.9995590000	0.0000060000
C	5.5252240000	0.8406380000	0.0000020000
C	4.8058910000	-0.4050660000	-0.0000010000
C	5.5425310000	-1.6188630000	-0.0000040000
H	5.0087850000	-2.5645380000	-0.0000050000
C	6.9226930000	-1.6109840000	-0.0000040000
H	7.4693450000	-2.5496030000	-0.0000060000
C	7.6331420000	-0.3856230000	-0.0000020000
H	8.7191090000	-0.3914720000	-0.0000020000
C	6.9463470000	0.8102300000	0.0000010000
H	7.4879320000	1.7528510000	0.0000030000
H	-0.2648020000	-2.5230640000	-0.0000070000
H	0.2648020000	2.5230640000	0.0000050000
H	-2.6001840000	2.4618650000	0.0000030000
H	2.6001850000	-2.4618650000	0.0000040000

ACID Plot Computations

General. The geometries were optimized at the TPSSh/SVP level of density functional theory using Gaussian 09 Rev D.01.[12] The anisotropy of the current density was calculated using our ACID method implemented in the Gaussian program (NMR=CSGT IOp(10/93=1).[13] The orientation of the magnetic field is chosen to be orthogonal to the ring planes and pointing towards the viewer. To quantify delocalization effects we use the critical isosurface values (CIV). At these points the gradients of the ACID scalar field are zero. The CIV values give the positions and the ACID values at the minima in the density of delocalized electrons (e.g., a small CIV value indicates a weak conjugation).

The ACID plot of the parent s-indacene (Figure S5, top) exhibits a distinct paratropic ring current (counterclockwise) along the indacene periphery. The CIV values along the ring periphery (0.133, 0.132, 0.111) indicate a strong (antiaromatic) conjugation. In contrast to the parent indacene, the dibenzo derivative **1`** (Figure S5, bottom) does not exhibit a distinct ring current along the periphery of the indacene unit. Diatropic ring currents are restricted to the terminal benzene rings, which disturb the paratropic ring current in the indacene core. The CIV values in the periphery of the indacene core are considerably smaller (CIV = 0.076, 0.074, 0.071) than in the parent system and confirm the weaker conjugation indicated by the current density.

The ACID plot of *anti*-IDBT **6`** (Figure S6, top) exhibits diatropic ring currents in the terminal benzene units and a paratropic ring current along the indacene unit. The smallest CIV in the core unit is 0.084; thus, the antiaromatic conjugation is weaker than in the parent system but stronger than in the dibenzo- (**1`**) and dinaphthaleno-fused (**8`** and **9`**, see Figure S7) derivatives. The antiaromaticity of the *syn*-IDBT **7`** (Figure S6, bottom) is more pronounced than in *anti*-IDBT **6`** and is almost as strong as in the parent indacene system. The *anti* conformation is energetically more favorable (ΔE = 1.70 kcal mol^{-1}; TPSSh/SVP level of theory) than the *syn*-IDBT, which is in good agreement with the weaker antiaromaticity of *anti*-IDBT.

The ACID plots of *anti*-IDN **8`** (Figure S3, top) and *syn*-IDN **9`** (Figure S3, bottom) exhibit diatropic ring currents along the naphthalene periphery which is disturbed in close vicinity to the paratropic ring current of the indacene core. The smallest CIVs in the core units are 0.079 in *anti*-IDN **8`** and 0.074 in *syn*-IDN **9`**; hence, the antiaromatic conjugation is stronger than in the dibenzo derivative **1`** but weaker than in the other investigated compounds. Surprisingly the *anti*-IDN **8`** exhibits the higher antiaromatic character but is energetically more favorable (ΔE = 4.39

kcal mol^{-1}) than the *syn*-IDN **9`**. This can be explained by steric repulsion between the two neighboring hydrogen atoms in the bay region of **9`**.

In conclusion, the parent system as well as *anti*-IDBT **6`**, *syn*-IDBT **7`**, *anti*-IDN **8`** and *syn*-IDN **9`** exhibit a distinct paratropic ring current along the ring periphery of the indacene unit. The antiaromatic character decreases from the parent system (CIV = 0.111) via the *syn*-IDBT **7`** (CIV = 0.099) to the *anti*-IDBT **6`** (CIV = 0.084). The antiaromatic character decreases further from the *anti*-IDN **8`** (CIV = 0.079) to the *syn*-IDN **9`** (CIV = 0.074) while the paratropic ring current in the indacene core slighty disturbs the aromatic ring current along the terminal naphthalene unit. The ACID plot of indenofluorene **1`** differs from the other five compounds. It exhibits diatropic ring currents in the outer benzene rings which disturb the paratropic ring current along the indacene core periphery which has the lowest CIV value of all investigated compounds (CIV = 0.071).

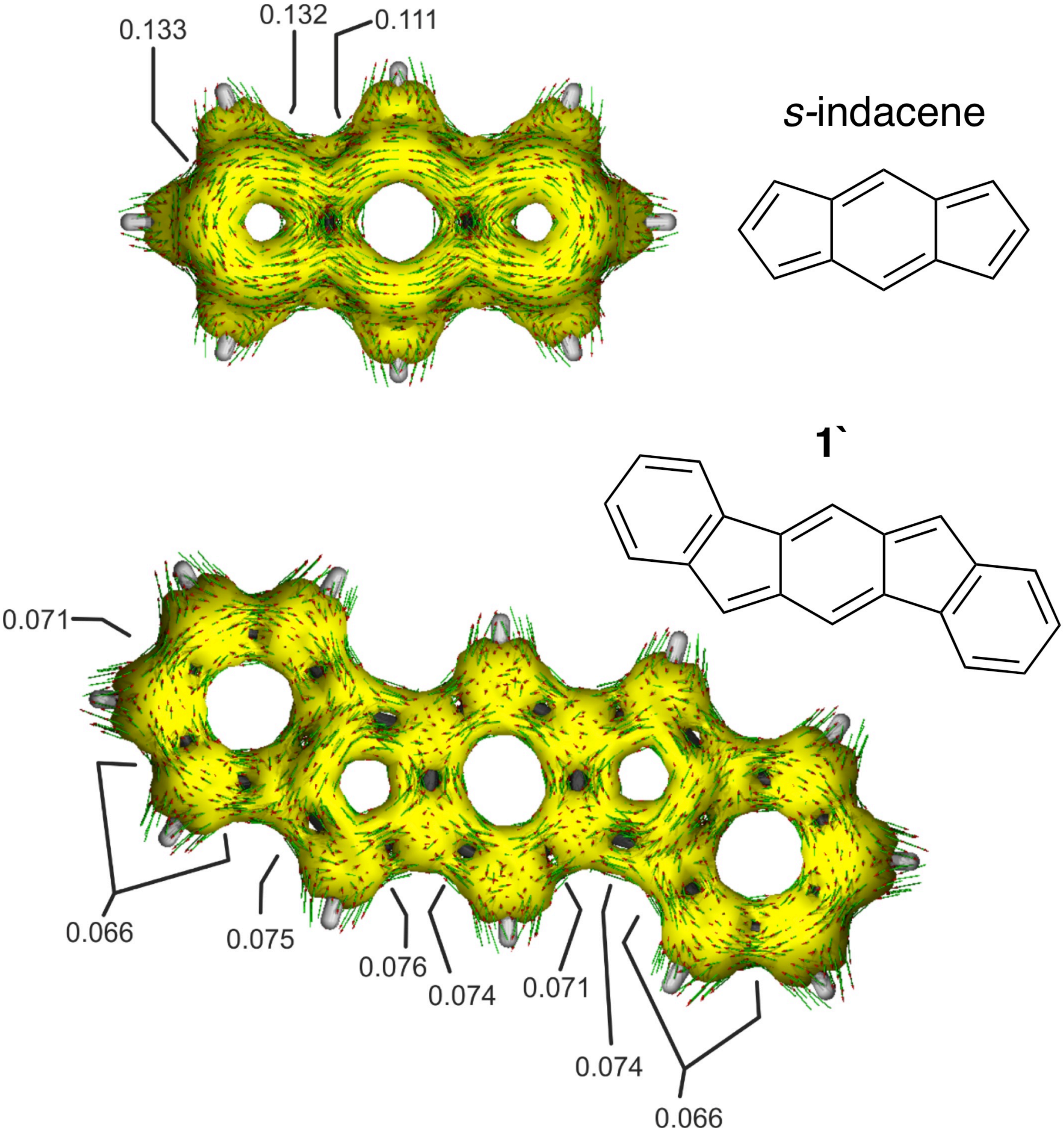

Figure S5. ACID plot of s-indacene (top) and indeno[1,2-b]fluorene **1`** (bottom) at an isosurface value (IV) of 0.05. Current density vectors (green arrows with red arrowheads) are plotted onto the isosurface. Positions and values of the critical isosurface values (CIV) are given.

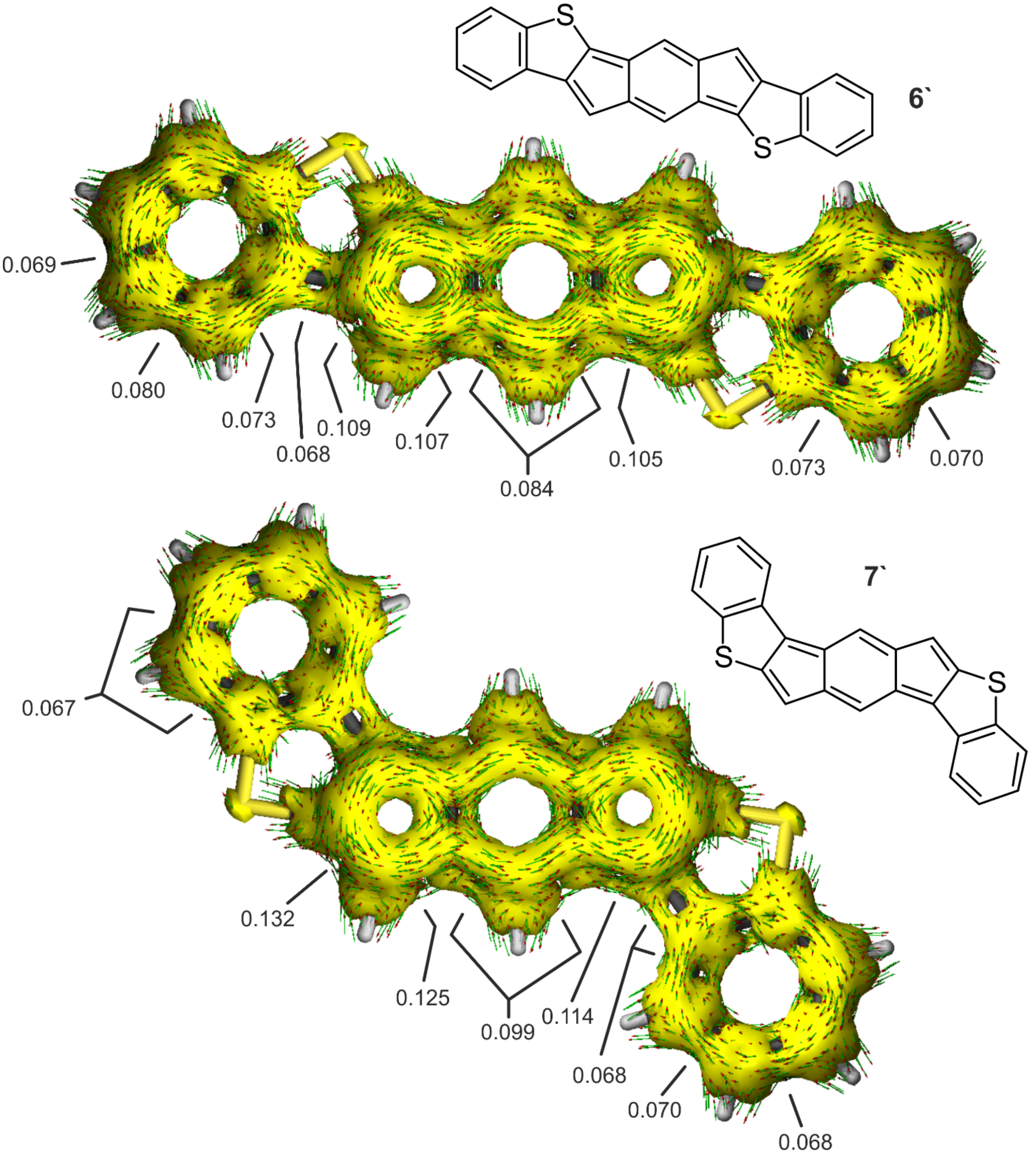

Figure S6. ACID plots of *anti*-IDBT **6`** (top) and *syn*-IDBT **7`** (bottom), at an isosurface value of 0.05 with current density vectors mapped onto the isosurface.

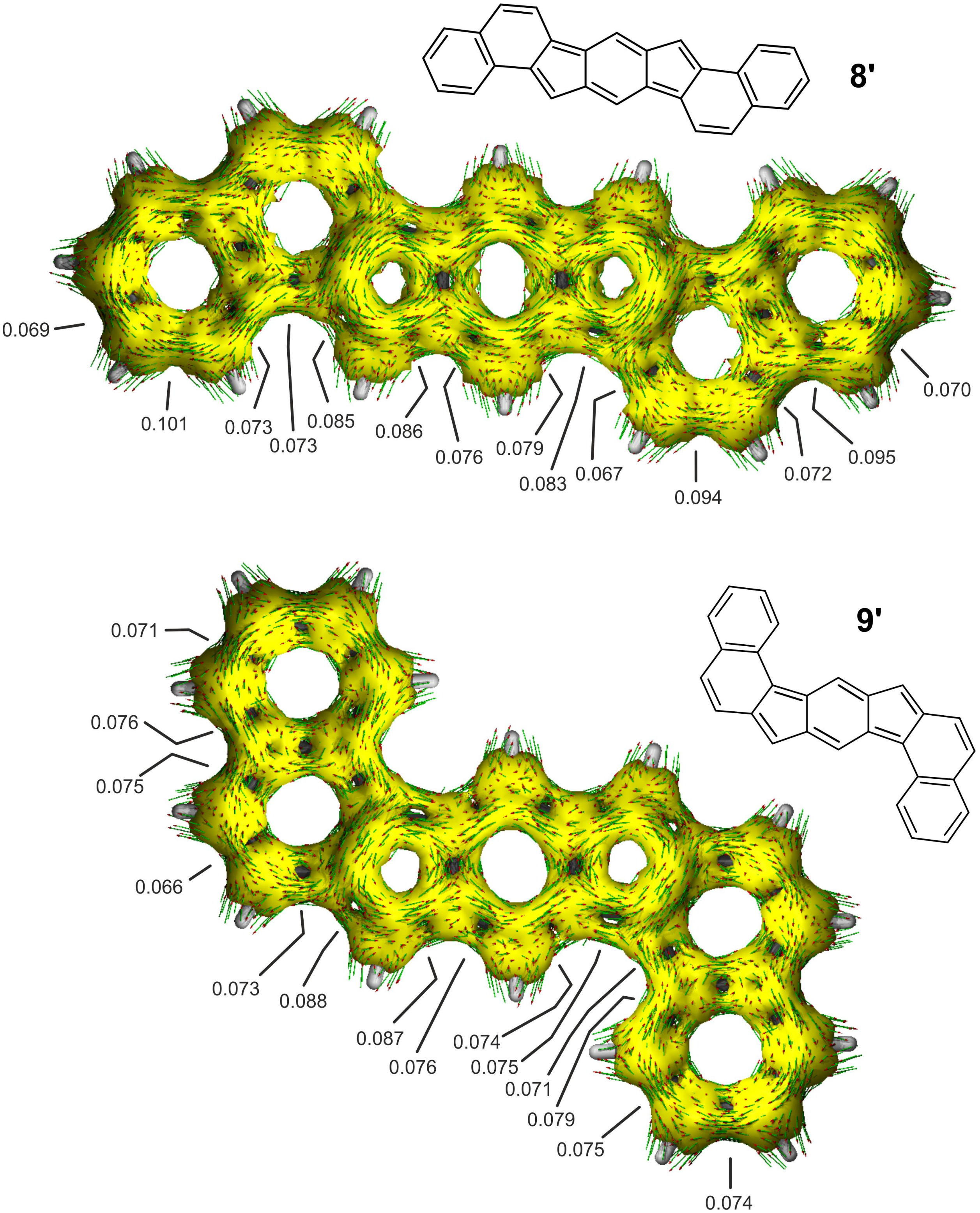

Figure S7. ACID plots of *anti*-IDN **8`** (top) and *syn*-IDN **9`** (bottom), at an isosurface value of 0.05 with current density vectors mapped onto the isosurface.

Cartesian Coordinates of TPSSh/SVP optimized structures

s-indacene

$E_{TPSSh/SVP}$ = -461.7533965 Hartree

NImag = 0

C	0.000000	0.723800	1.204610
C	0.000000	-0.723800	1.204610
C	0.000000	-1.443240	0.000000
C	0.000000	-0.723800	-1.204610
C	0.000000	0.723800	-1.204610
C	0.000000	1.443240	0.000000
C	0.000000	1.142930	2.568230
C	0.000000	0.000000	3.391340
C	0.000000	-1.142930	2.568230
H	0.000000	-2.537720	0.000000
H	0.000000	2.537720	0.000000
H	0.000000	2.178280	2.912810
H	0.000000	0.000000	4.480870
H	0.000000	-2.178280	2.912810
C	0.000000	-1.142930	-2.568230
C	0.000000	1.142930	-2.568230
C	0.000000	0.000000	-3.391340
H	0.000000	0.000000	-4.480870
H	0.000000	-2.178280	-2.912810
H	0.000000	2.178280	-2.912810

Indeno[1,2-*b*]fluorene

$E_{TPSSh/SVP}$ = -768.8865800 Hartree

NImag = 0

C	0.003030	1.404470	0.000000
C	1.259970	0.647730	0.000000
C	1.259970	-0.721690	0.000000
C	-0.003030	-1.404470	0.000000
C	-1.259970	-0.647730	0.000000
C	-1.259970	0.721690	0.000000
C	0.298940	2.757240	0.000000
C	1.740120	2.928850	0.000000
C	2.345960	1.633740	0.000000
H	2.189930	-1.296780	0.000000
H	-2.189930	1.296780	0.000000
H	-0.428320	3.570720	0.000000
C	-0.298940	-2.757240	0.000000
C	-2.345960	-1.633740	0.000000
C	-1.740120	-2.928850	0.000000
H	0.428320	-3.570720	0.000000
C	3.734020	1.507090	0.000000
C	2.535050	4.082470	0.000000
C	4.521610	2.670150	0.000000
H	5.610830	2.583530	0.000000
C	3.929000	3.941910	0.000000
H	4.563200	4.831660	0.000000
C	-2.535050	-4.082470	0.000000
C	-3.734020	-1.507090	0.000000
C	-4.521610	-2.670150	0.000000
H	-5.610830	-2.583530	0.000000
C	-3.929000	-3.941910	0.000000
H	-4.563200	-4.831660	0.000000
H	-2.078070	-5.075380	0.000000
H	-4.209800	-0.523070	0.000000
H	4.209800	0.523070	0.000000
H	2.078070	5.075380	0.000000

anti-IDBT 6`

$E_{TPSSh/SVP}$ = -1717.3890610 Hartree

NImag = 0

C	1.190310	-0.753610	0.000000
C	-0.118820	-1.402650	0.000000
C	-1.289960	-0.656410	0.000000
C	-1.190310	0.753610	0.000000
C	0.118820	1.402650	0.000000
C	1.289960	0.656410	0.000000
C	2.192380	-1.749680	0.000000
C	1.559020	-3.021290	0.000000
C	0.159150	-2.811730	0.000000
H	-2.270640	-1.140500	0.000000
H	2.270640	1.140500	0.000000
H	3.267280	-1.565100	0.000000
C	-2.192380	1.749680	0.000000
C	-0.159150	2.811730	0.000000
C	-1.559020	3.021290	0.000000
H	-3.267280	1.565100	0.000000
C	-0.746940	5.237300	0.000000
C	0.746940	-5.237300	0.000000
C	1.913320	-4.420160	0.000000
S	-0.746940	-4.282600	0.000000
S	0.746940	4.282600	0.000000
C	-1.913320	4.420160	0.000000
C	-0.820340	6.632890	0.000000
C	-3.171380	5.051370	0.000000
C	-2.083260	7.230890	0.000000
H	-2.162620	8.320340	0.000000
C	-3.248290	6.443640	0.000000
H	-4.226550	6.930000	0.000000
H	0.085880	7.242220	0.000000
H	-4.082100	4.448060	0.000000
C	0.820340	-6.632890	0.000000
C	3.171380	-5.051370	0.000000
C	3.248290	-6.443640	0.000000
H	4.226550	-6.930000	0.000000
C	2.083260	-7.230890	0.000000
H	2.162620	-8.320340	0.000000
H	4.082100	-4.448060	0.000000
H	-0.085880	-7.242220	0.000000

syn-IDBT 7`

$E_{TPSSh/SVP}$ = -1717.3863472 Hartree

NImag = 0

C	-0.004560	1.399670	0.000000
C	1.254750	0.663720	0.000000
C	1.254750	-0.716820	0.000000
C	0.004560	-1.399670	0.000000
C	-1.254750	-0.663720	0.000000
C	-1.254750	0.716820	0.000000
C	0.273880	2.777520	0.000000
C	1.689640	2.922810	0.000000
C	2.315180	1.664230	0.000000
H	2.184710	-1.290640	0.000000
H	-2.184710	1.290640	0.000000
H	-0.461650	3.581930	0.000000
C	-0.273880	-2.777520	0.000000
C	-2.315180	-1.664230	0.000000
C	-1.689640	-2.922810	0.000000
H	0.461650	-3.581930	0.000000
C	-3.742610	-1.756990	0.000000
C	-4.162850	-3.126430	0.000000
S	-2.813240	-4.254830	0.000000
C	3.742610	1.756990	0.000000
C	4.162850	3.126430	0.000000
S	2.813240	4.254830	0.000000
C	4.737750	0.751980	0.000000
C	5.517850	3.478710	0.000000
C	6.471650	2.461710	0.000000
H	7.533110	2.719990	0.000000
C	6.081480	1.106460	0.000000
H	6.846570	0.326750	0.000000
C	-5.517850	-3.478710	0.000000
C	-4.737750	-0.751980	0.000000
C	-6.081480	-1.106460	0.000000
H	-6.846570	-0.326750	0.000000
C	-6.471650	-2.461710	0.000000
H	-7.533110	-2.719990	0.000000
H	5.821190	4.527810	0.000000
H	4.447920	-0.300440	0.000000
H	-5.821190	-4.527810	0.000000
H	-4.447920	0.300440	0.000000

***anti*-IDN 8`**

$E_{TPSSh/SVP}$ = -1075.9884317 Hartree

NImag = 0

C	-0.617830	1.272650	0.000000
C	0.835100	1.125470	0.000000
C	1.439640	-0.171080	0.000000
C	0.617830	-1.272650	0.000000
C	-0.835100	-1.125470	0.000000
C	-1.439640	0.171080	0.000000
C	-0.894670	2.708570	0.000000
C	0.352600	3.385060	0.000000
C	1.403610	2.396400	0.000000
H	2.529100	-0.266110	0.000000
H	-2.529100	0.266110	0.000000
H	2.473780	2.607140	0.000000
C	0.894670	-2.708570	0.000000
C	-1.403610	-2.396400	0.000000
C	-0.352600	-3.385060	0.000000
H	-2.473780	-2.607140	0.000000
C	-2.105410	3.428400	0.000000
C	0.413210	4.813160	0.000000
C	2.105410	-3.428400	0.000000
C	-0.413210	-4.813160	0.000000
C	2.067570	-4.813620	0.000000
H	2.999830	-5.384490	0.000000
C	-2.067570	4.813620	0.000000
H	-2.999830	5.384490	0.000000
C	-0.835100	5.532660	0.000000
C	0.835100	-5.532660	0.000000
C	0.806680	-6.955030	0.000000
H	1.755840	-7.497820	0.000000
C	-1.626360	-5.551940	0.000000
H	-2.578120	-5.016070	0.000000
C	-1.616830	-6.934930	0.000000
C	-0.390670	-7.644360	0.000000
C	1.626360	5.551940	0.000000
C	-0.806680	6.955030	0.000000
C	0.390670	7.644360	0.000000
H	0.395830	8.736970	0.000000
C	1.616830	6.934930	0.000000
H	2.560530	7.485880	0.000000
H	2.578120	5.016070	0.000000
H	-1.755840	7.497820	0.000000
H	-3.064940	2.905640	0.000000
H	-0.395830	-8.736970	0.000000
H	-2.560530	-7.485880	0.000000
H	3.064940	-2.905640	0.000000

***syn*-IDN 9`**

$E_{TPSSh/SVP}$ = -1075.9814368 Hartree

NImag = 0

C	1.273970	0.667000	0.000000
C	0.000050	1.390940	0.000000
C	-1.258020	0.708010	0.000000
C	-1.273970	-0.667000	0.000000
C	-0.000050	-1.390940	0.000000
C	1.258020	-0.708010	0.000000
C	2.338780	1.681130	0.000000
C	1.697210	2.947840	0.000000
C	0.266840	2.751040	0.000000
H	-2.170230	1.306370	0.000000
H	2.170230	-1.306370	0.000000
H	-0.475370	3.550970	0.000000
C	-2.338780	-1.681130	0.000000
C	-0.266840	-2.751040	0.000000
C	-1.697210	-2.947840	0.000000
H	0.475370	-3.550970	0.000000
C	3.762300	1.602850	0.000000
C	2.437780	4.154750	0.000000
C	-3.762300	-1.602850	0.000000
C	-2.437780	-4.154750	0.000000
C	-4.503610	-2.845190	0.000000
C	4.503610	2.845190	0.000000
C	3.815240	4.095150	0.000000
C	-3.815240	-4.095150	0.000000
C	4.503610	0.385550	0.000000
C	5.924760	2.801760	0.000000
C	6.606690	1.600910	0.000000
H	7.699110	1.587720	0.000000
C	5.884910	0.382520	0.000000
H	6.424800	-0.567460	0.000000
C	-4.503610	-0.385550	0.000000
C	-5.924760	-2.801760	0.000000
C	-5.884910	-0.382520	0.000000
H	-6.424800	0.567460	0.000000
C	-6.606690	-1.600910	0.000000
H	-7.699110	-1.587720	0.000000
H	-1.919660	-5.116830	0.000000
H	-4.408160	-5.013320	0.000000
H	-6.474050	-3.747110	0.000000
H	-3.973050	0.565270	0.000000
H	4.408160	5.013320	0.000000
H	1.919660	5.116830	0.000000
H	6.474050	3.747110	0.000000
H	3.973050	-0.565270	0.000000

DFT and TDDFT Computational Details

General. Analyses of neutral ground-state and radical-ion states were carried out using density functional theory (DFT) with the B3LYP functional the cc-pVDZ basis set. Time-dependent DFT (TDDFT) calculations were carried out at the same level of theory to examine the low-lying excited states. Gaussian 09 (revision A.02) was used for these isolated molecule and dimer calculations.[14] DFT band-structure calculations were carried out with the VASP program[15] using PAW potentials (v.52)[15,16] and the GGA-PBE functional.[17] The kinetic energy cut-off was set to 400 eV. The Brillouin zone was sampled with the following Monkhorst-Pack grids: 3×2×1, 2×3×2, 2×2×2 for the **6d**, **6f**, **7d** crystals, respectively. Gaussian smearing with a width of 0.05 eV was employed. We report band structures of geometrically relaxed and unrelaxed (experimental) structures. Relaxations were performed with a fixed unit cell until the forces were smaller than 0.01 eV/Å. Van der Waals interactions were modeled with the DFT-D2 functional by Grimme.[18] We used the AFLOW software (version 31024) to order the unit cell vectors and to obtain a standardized path in the reciprocal space.[19]

Table S1. $S_0 \rightarrow S_2$ vertical transition energies, wavelengths, oscillator strengths (*f*), and the corresponding electronic configurations as determined with TDDFT at the B3LYP/cc-pVDZ level of theory.

	$S_0 \rightarrow S_2$ (eV)	$S_0 \rightarrow S_2$ (nm)	*f*	Configuration
**6a**	2.03	610		HOMO−1 → LUMO (93%)
**6b**	1.91	650		HOMO → LUMO (97%)
**6c**	1.92	644		HOMO → LUMO (96%)
**6d**	1.83	676		HOMO−1 → LUMO (97%)
**6e**	1.84	672		HOMO → LUMO (97%)
**6f**	1.76	703		HOMO → LUMO (99%)
**7a**	1.97	629		HOMO−1 → LUMO (97%)
**7b**	1.79	693		HOMO → LUMO (100%)
**7c**	1.81	683		HOMO−1 → LUMO (100%)
**7d**	1.78	698		HOMO−1 → LUMO (99%)
**7e**	1.74	713		HOMO−1 → LUMO (100%)
**7f**	1.72	722		HOMO−1 → LUMO (100%)

Table S2. Adiabatic ionization potentials (AIP) and electron affinities (AEA), energies of the HOMO–1 (E_{HOMO-1}), HOMO (E_{HOMO}), and LUMO (E_{LUMO}), and the HOMO-LUMO energy gap (E_{gap}) as determined at the B3LYP/cc-pVDZ level of theory.

	**AIP (eV)**	**AEA (eV)**	**E_{HOMO-1} (eV)**	**E_{HOMO} (eV)**	**E_{LUMO} (eV)**	**E_{gap} (eV)**
**6a**	6.04	−2.03	−5.10	−5.06	−3.05	2.01
**6b**	5.88	−2.06	−5.03	−4.95	−3.05	1.90
**6c**	6.02	−2.10	−5.10	−5.05	−3.13	1.91
**6d**	6.43	−2.60	−5.45	−5.43	−3.63	1.80
**6e**	6.48	−2.69	−5.52	−5.51	−3.68	1.83
**6f**	6.00	−2.43	−5.18	−5.07	−3.40	1.67
**7a**	6.03	−2.21	−5.21	−5.04	−3.18	1.85
**7b**	5.89	−2.25	−4.99	−4.98	−3.20	1.78
**7c**	6.05	−2.29	−5.09	−5.06	−3.29	1.77
**7d**	6.37	−2.74	−5.54	−5.37	−3.75	1.62
**7e**	6.47	−2.89	−5.57	−5.48	−3.85	1.63
**7f**	6.05	−2.56	−5.16	−5.13	−3.51	1.63

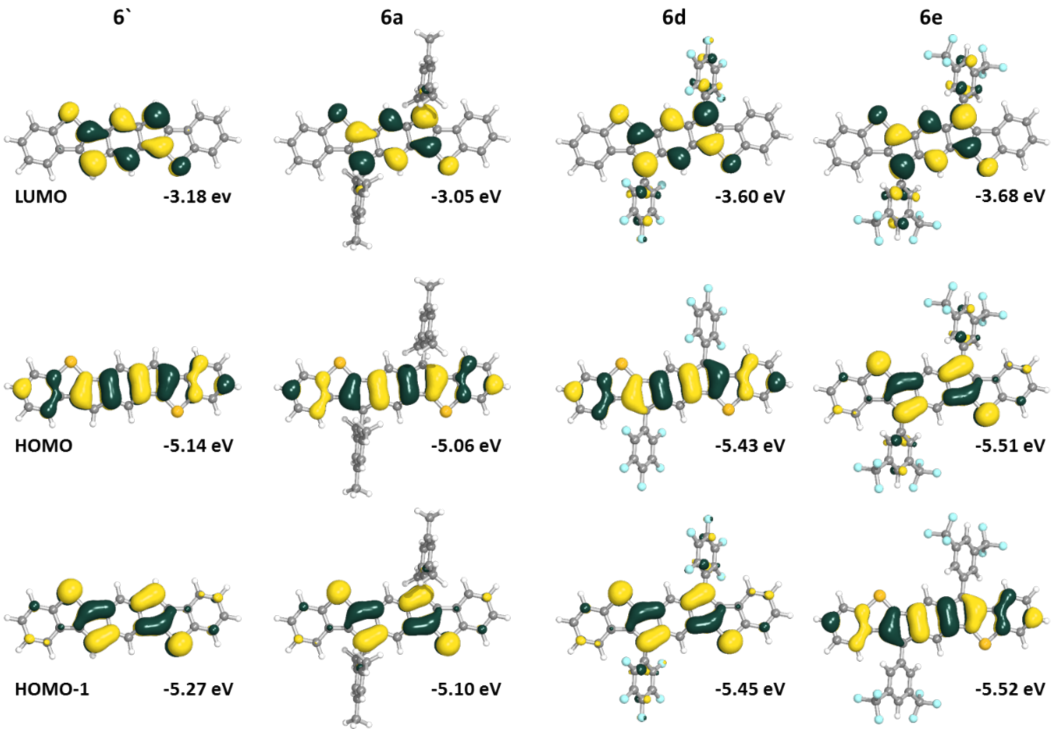

Figure S8. Pictorial representations of select frontier molecular orbitals (and energies) of **6`**, **6a**, **6d**, and **6e** as determined at the B3LYP/cc-pVDZ level of theory.

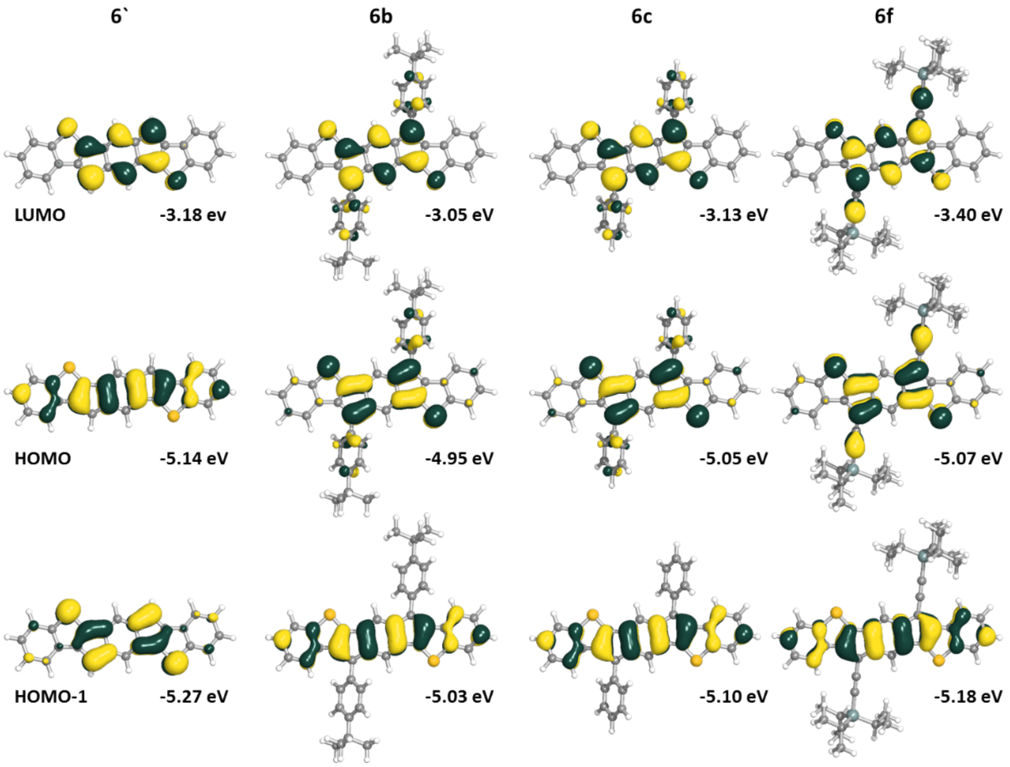

Figure S9. Pictorial representations of select frontier molecular orbitals (and energies) of **6`**, **6b**, **6c**, and **6f** as determined at the B3LYP/cc-pVDZ level of theory.

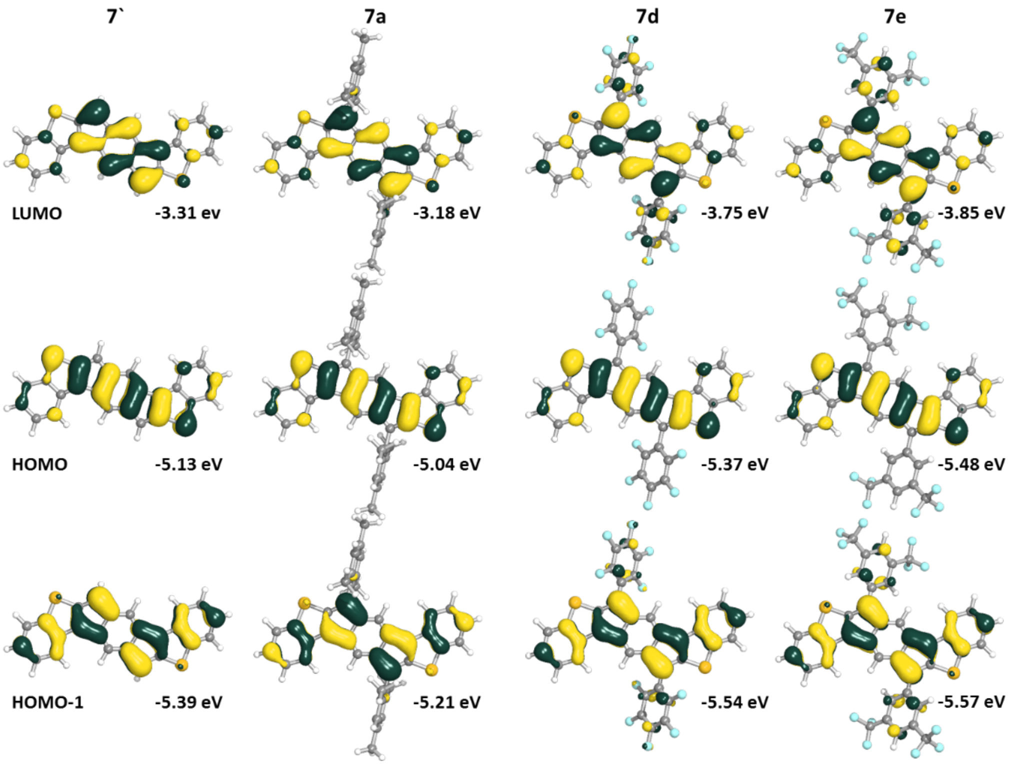

Figure S10. Pictorial representations of select frontier molecular orbitals (and energies) of **7`**, **7a**, **7d**, and **7e** as determined at the B3LYP/cc-pVDZ level of theory.

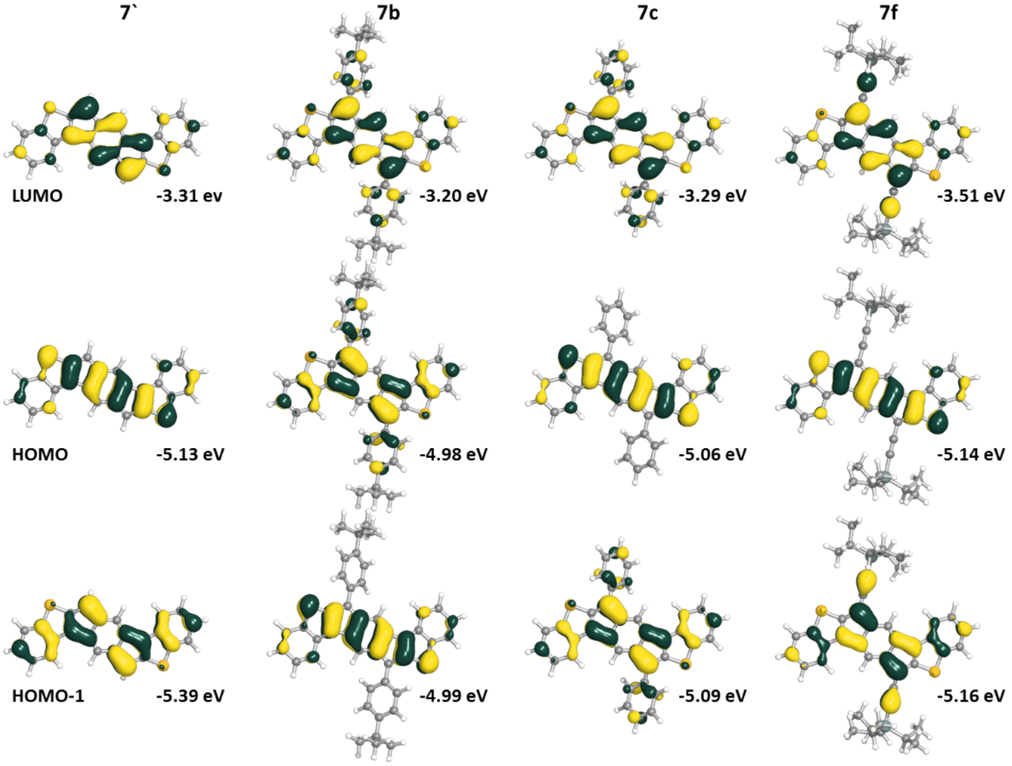

Figure S11. Pictorial representations of select frontier molecular orbitals (and energies) of **7`**, **7b**, **7c**, and **7f** as determined at the B3LYP/cc-pVDZ level of theory.

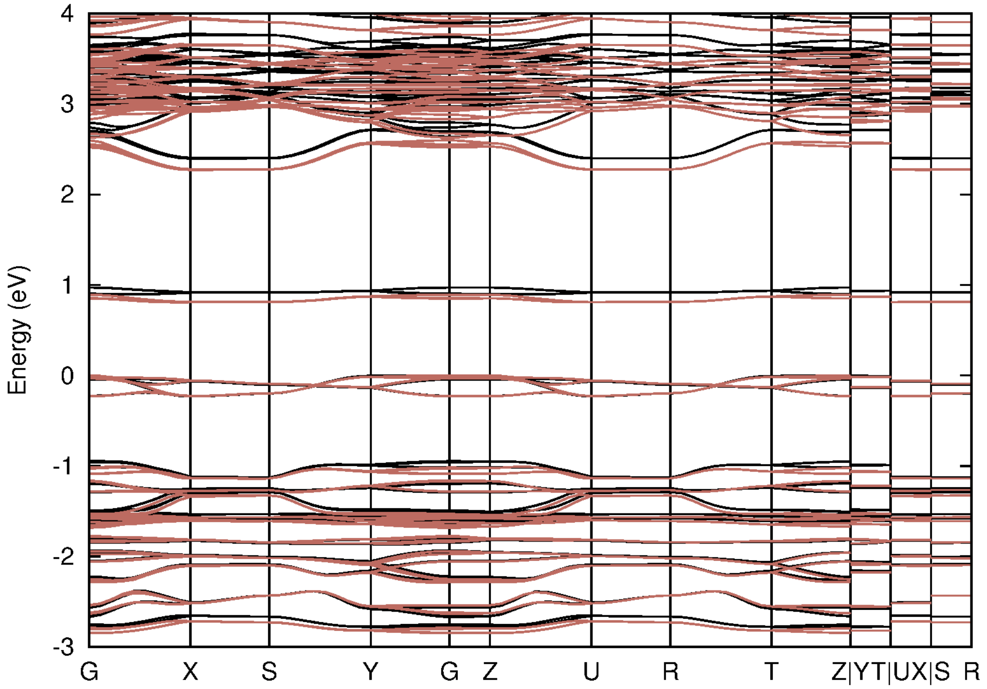

Figure S12. Electronic band structure of the experimental (black) and relaxed (red) structures of **6d** in the orthorhombic lattice (ORC). The origin of the energy axis is set at the top of the valence band.

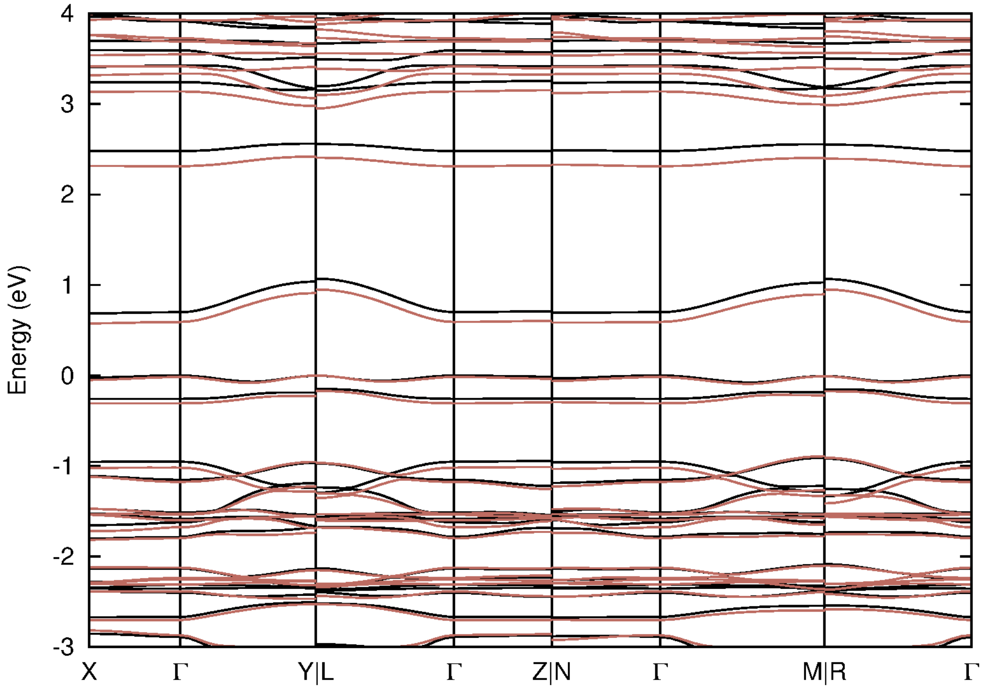

Figure S13. Electronic band structure of the experimental (black) and relaxed (red) structures of **6f** in the triclinic lattice (TRI_{1a}). The origin of the energy axis is set at the top of the valence band.

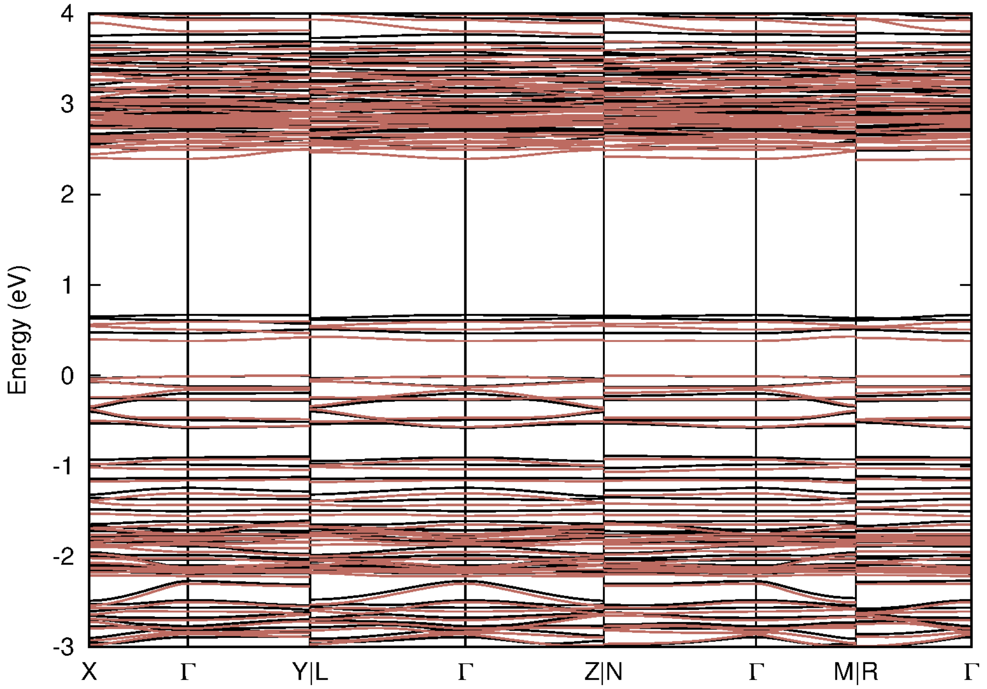

Figure S14. Electronic band structure of the experimental (black) and relaxed (red) structures of **7d** in the triclinic lattice (TRI_{1b}). The origin of the energy axis is set at the top of the valence band.

References

1. Young, B. S.; Chase, D. T.; Marshall, J. L.; Vonnegut, C. L.; Zakharov, L. N.; Haley, M. M. *Chem. Sci.* **2014**, *5*, 1008-1014.
2. Wong, K.-T.; Chao, T.-C.; Chi, L.-C.; Chu, Y.-Y.; Balaiah, A.; Chiu, S.-F.; Liu, Y.-H.; Wang, Y. *Org. Lett.* **2006**, *8*, 5033-5036.
3. Nakagawa, H.; Kawai, S.; Nakashima, T.; Kawai, T. *Org. Lett.* **2009**, *11*, 1475-1478.
4. Reiss, H.; Heller, A. *J. Phys. Chem.* **1985**, *89*, 4207-4213.
5. Sheldrick, G. M. *Bruker/Siemens Area Detector Absorption Correction Program*; Bruker AXS: Madison, WI, 1998.
6. Sheldrick, G. M. *Acta Crystallogr. Sect. A: Found. Crystallogr.* **2007**, *64*, 112-122.
7. Frisch, M. J.; Trucks, G. W.; Schlegel, H. B.; Scuseria, G. E.; Robb, M. A.; Cheeseman, J. R.; Scalmani, G.; Barone, V.; Mennucci, B.; Petersson, G. A.; Nakatsuji, H.; Caricato, M.; Li, X.; Hratchian, H. P.; Izmaylov, A. F.; Bloino, J.; Zheng, G.; Sonnenberg, J. L.; Hada, M.; Ehara, M.; Toyota, K.; Fukuda, R.; Hasegawa, J.; Ishida, M.; Nakajima, T.; Honda, Y.; Kitao, O.; Nakai, H.; Vreven, T.; Montgomery Jr., J. A.; Peralta, J. E.; Ogliaro, F.; Bearpark, M. J.; Heyd, J.; Brothers, E. N.; Kudin, K. N.; Staroverov, V. N.; Kobayashi, R.; Normand, J.; Raghavachari, K.; Rendell, A. P.; Burant, J. C.; Iyengar, S. S.; Tomasi, J.; Cossi, M.; Rega, N.; Millam, N. J.; Klene, M.; Knox, J. E.; Cross, J. B.; Bakken, V.; Adamo, C.; Jaramillo, J.; Gomperts, R.; Stratmann, R. E.; Yazyev, O.; Austin, A. J.; Cammi, R.; Pomelli, C.; Ochterski, J. W.; Martin, R. L.; Morokuma, K.; Zakrzewski, V. G.; Voth, G. A.; Salvador, P.; Dannenberg, J. J.; Dapprich, S.; Daniels, A. D.; Farkas, Ö.; Foresman, J. B.; Ortiz, J. V.; Cioslowski, J.; Fox, D. J. *Gaussian 09*; Gaussian, Inc.: Wallingford, CT, USA, 2009.
8. Gershoni-Poranne, R.; Stanger, A. *Chem. - Eur. J.* **2014**, *20*, 5673-5688.
9. Stanger, A. *J. Org. Chem.* **2006**, *71*, 883-893.
10. Stanger, A. *J. Org. Chem.* **2010**, *75*, 2281-2288.
11. Rahalkar, A.; Stanger, A. http://schulich.technion.ac.il/Amnon_Stanger.htm.
12. Frisch, M. J.; Trucks, G. W.; Schlegel, H. B.; Scuseria, G. E.; Robb, M. A.; Cheeseman, J. R.; Scalmani, G.; Barone, V.; Mennucci, B.; Petersson, G. A.; Nakatsuji, H.; Caricato, M.; Li, X.; Hratchian, H. P.; Izmaylov, A. F.; Bloino, J.; Zheng, G.; Sonnenberg, J. L.; Hada, M.; Ehara, M.; Toyota, K.; Fukuda, R.; Hasegawa, J.; Ishida, M.; Nakajima, T.; Honda, Y.; Kitao, O.; Nakai, H.; Vreven, T.; Montgomery Jr., J. A.; Peralta, J. E.; Ogliaro, F.; Bearpark, M. J.;

Heyd, J.; Brothers, E. N.; Kudin, K. N.; Staroverov, V. N.; Kobayashi, R.; Normand, J.; Raghavachari, K.; Rendell, A. P.; Burant, J. C.; Iyengar, S. S.; Tomasi, J.; Cossi, M.; Rega, N.; Millam, N. J.; Klene, M.; Knox, J. E.; Cross, J. B.; Bakken, V.; Adamo, C.; Jaramillo, J.; Gomperts, R.; Stratmann, R. E.; Yazyev, O.; Austin, A. J.; Cammi, R.; Pomelli, C.; Ochterski, J. W.; Martin, R. L.; Morokuma, K.; Zakrzewski, V. G.; Voth, G. A.; Salvador, P.; Dannenberg, J. J.; Dapprich, S.; Daniels, A. D.; Farkas, Ö.; Foresman, J. B.; Ortiz, J. V.; Cioslowski, J.; Fox, D. J. *Gaussian 09, Revision D.01*; Gaussian, Inc.: Wallingford, CT, USA, 2009.

13. Herges, R.; Geuenich, D. *J. Phys. Chem. A* **2001**, *105*, 3214-3220.
14. Frisch, M. J.; Trucks, G. W.; Schlegel, H. B.; Scuseria, G. E.; Robb, M. A.; Cheeseman, J. R.; Scalmani, G.; Barone, V.; Mennucci, B.; Petersson, G. A.; Nakatsuji, H.; Caricato, M.; Li, X.; Hratchian, H. P.; Izmaylov, A. F.; Bloino, J.; Zheng, G.; Sonnenberg, J. L.; Hada, M.; Ehara, M.; Toyota, K.; Fukuda, R.; Hasegawa, J.; Ishida, M.; Nakajima, T.; Honda, Y.; Kitao, O.; Nakai, H.; Vreven, T.; Montgomery Jr., J. A.; Peralta, J. E.; Ogliaro, F.; Bearpark, M. J.; Heyd, J.; Brothers, E. N.; Kudin, K. N.; Staroverov, V. N.; Kobayashi, R.; Normand, J.; Raghavachari, K.; Rendell, A. P.; Burant, J. C.; Iyengar, S. S.; Tomasi, J.; Cossi, M.; Rega, N.; Millam, N. J.; Klene, M.; Knox, J. E.; Cross, J. B.; Bakken, V.; Adamo, C.; Jaramillo, J.; Gomperts, R.; Stratmann, R. E.; Yazyev, O.; Austin, A. J.; Cammi, R.; Pomelli, C.; Ochterski, J. W.; Martin, R. L.; Morokuma, K.; Zakrzewski, V. G.; Voth, G. A.; Salvador, P.; Dannenberg, J. J.; Dapprich, S.; Daniels, A. D.; Farkas, Ö.; Foresman, J. B.; Ortiz, J. V.; Cioslowski, J.; Fox, D. J. *Gaussian 09, Revision A.02*; Gaussian, Inc.: Wallingford, CT, USA, 2009.
15. Kresse, G.; Furthmüller, J. *Phys. Rev. B* **1996**, *54*, 11169-11186.
16. Blöchl, P. E. *Phys. Rev. B* **1994**, *50*, 17953-17979.
17. Perdew, J. P.; Burke, K.; Ernzerhof, M. *Phys. Rev. Lett.* **1996**, *77*, 3865-3868.
18. Grimme, S. *J. Comp. Chem.* **2006**, *27*, 1787-1799.
19. Setyawan, W.; Curtarolo, S. *Comp. Mater. Sci.* **2010**, *49*, 299-312.

Copies of NMR Spectra

^{1}H NMR (600 MHz, 1,1,2,2,-tetrachloroethane-d_2) δ 7.58 (d, J = 7.7 Hz, 4H), 7.54 (d, J = 8.0 Hz, 2H), 7.48 (d, J = 7.4 Hz, 2H), 7.28 (t, J = 7.4 Hz, 2H), 7.24 – 7.18 (m, 2H), 7.18 – 7.10 (m, 4H), 6.64 (s, 2H), 1.53 (s, 18H).

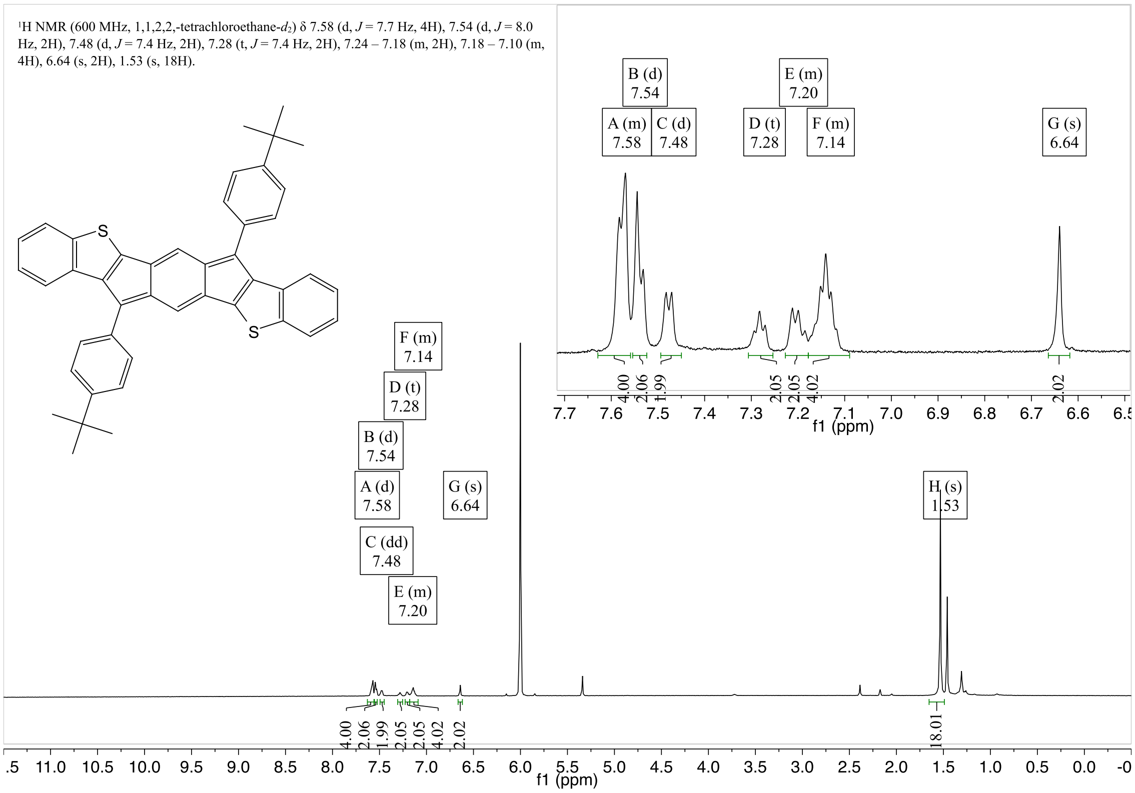

^{1}H NMR (500 MHz, 1,1,2,2-tetrachloroethane-d_2) δ 7.67 – 7.54 (m, 6H), 7.54 – 7.48 (m, 2H), 7.37 (s, 2H), 7.28 (t, J = 7.7 Hz, 2H), 7.24 – 7.17 (m, 2H), 7.12 (s, 4H), 6.56 (s, 2H).

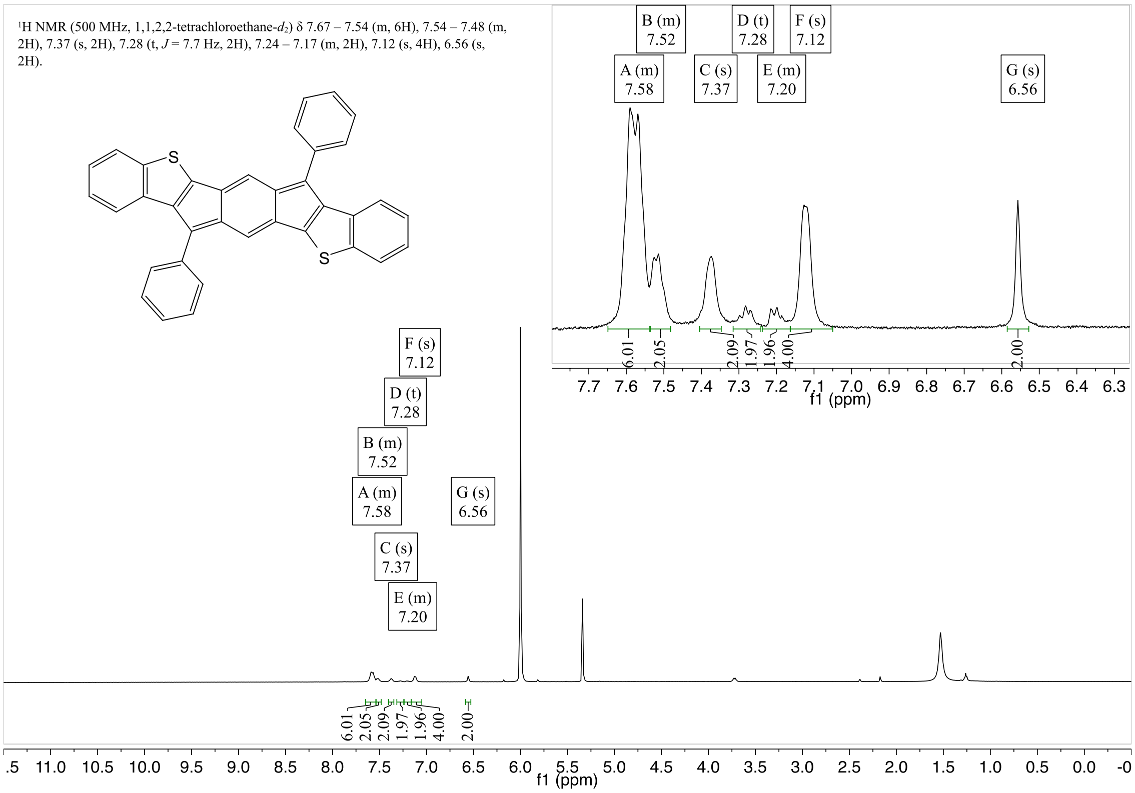

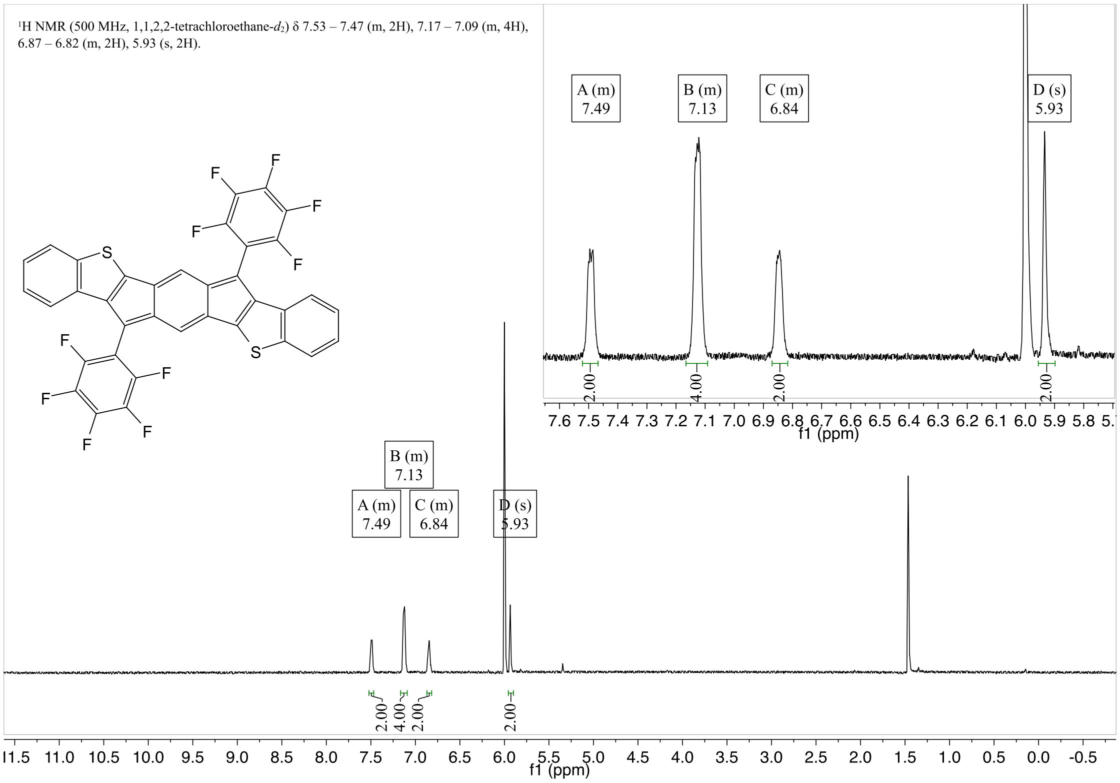

1H NMR (500 MHz, 1,1,2,2-tetrachloroethane-d2) δ 7.53 – 7.47 (m, 2H), 7.17 – 7.09 (m, 4H), 6.87 – 6.82 (m, 2H), 5.93 (s, 2H).
A (m) 7.49
B (m) 7.13
C (m) 6.84
D (s) 5.93
2.00
4.00
2.00
2.00
f1 (ppm)

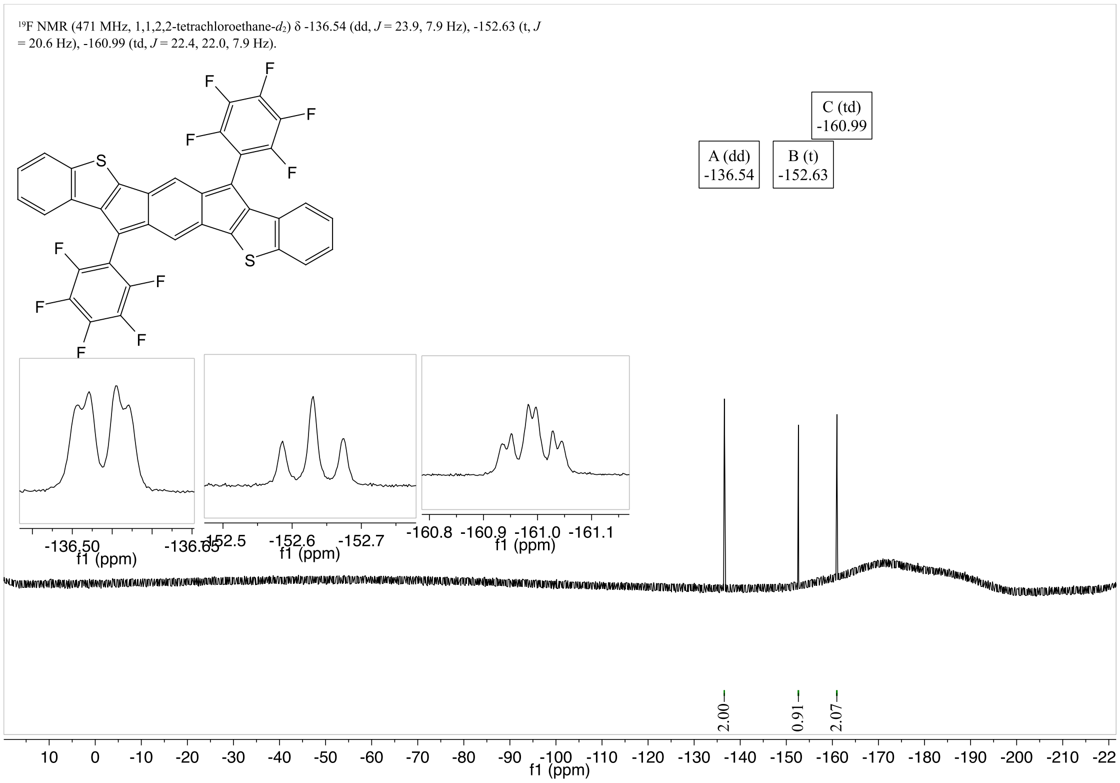

19F NMR (471 MHz, 1,1,2,2-tetrachloroethane-d2) δ -136.54 (dd, J = 23.9, 7.9 Hz), -152.63 (t, J = 20.6 Hz), -160.99 (td, J = 22.4, 22.0, 7.9 Hz).
A (dd) -136.54
B (t) -152.63
C (td) -160.99
2.00
0.91
2.07
f1 (ppm)

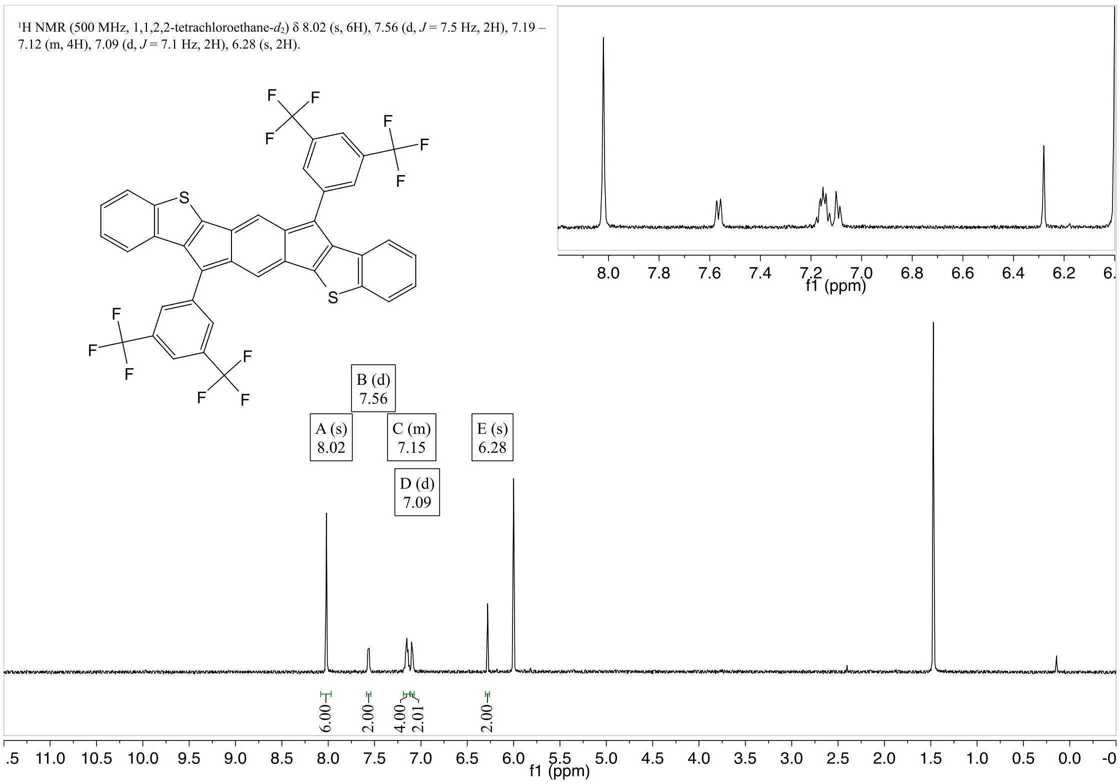
¹H NMR (500 MHz, 1,1,2,2-tetrachloroethane-d_2) δ 8.02 (s, 6H), 7.56 (d, J = 7.5 Hz, 2H), 7.19 – 7.12 (m, 4H), 7.09 (d, J = 7.1 Hz, 2H), 6.28 (s, 2H).
A (s) 8.02
B (d) 7.56
C (m) 7.15
D (d) 7.09
E (s) 6.28
6.00
2.00
4.00
2.01
2.00
f1 (ppm)

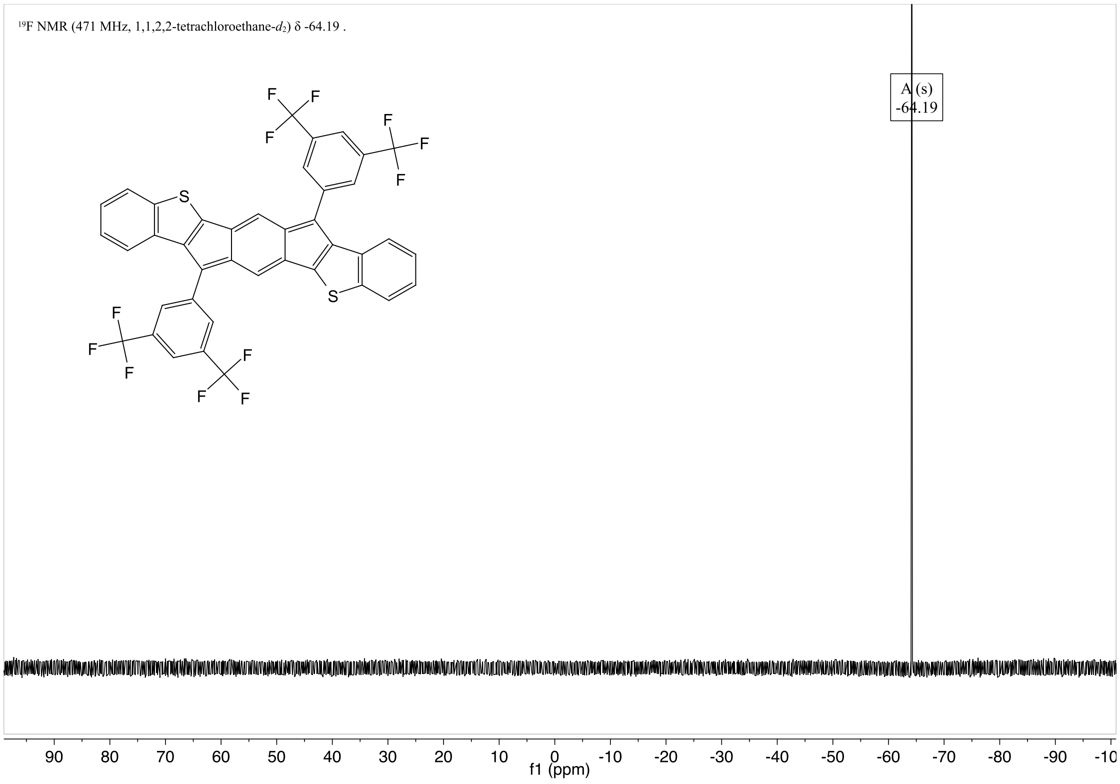
¹⁹F NMR (471 MHz, 1,1,2,2-tetrachloroethane-d_2) δ -64.19 .
A (s) -64.19
f1 (ppm)

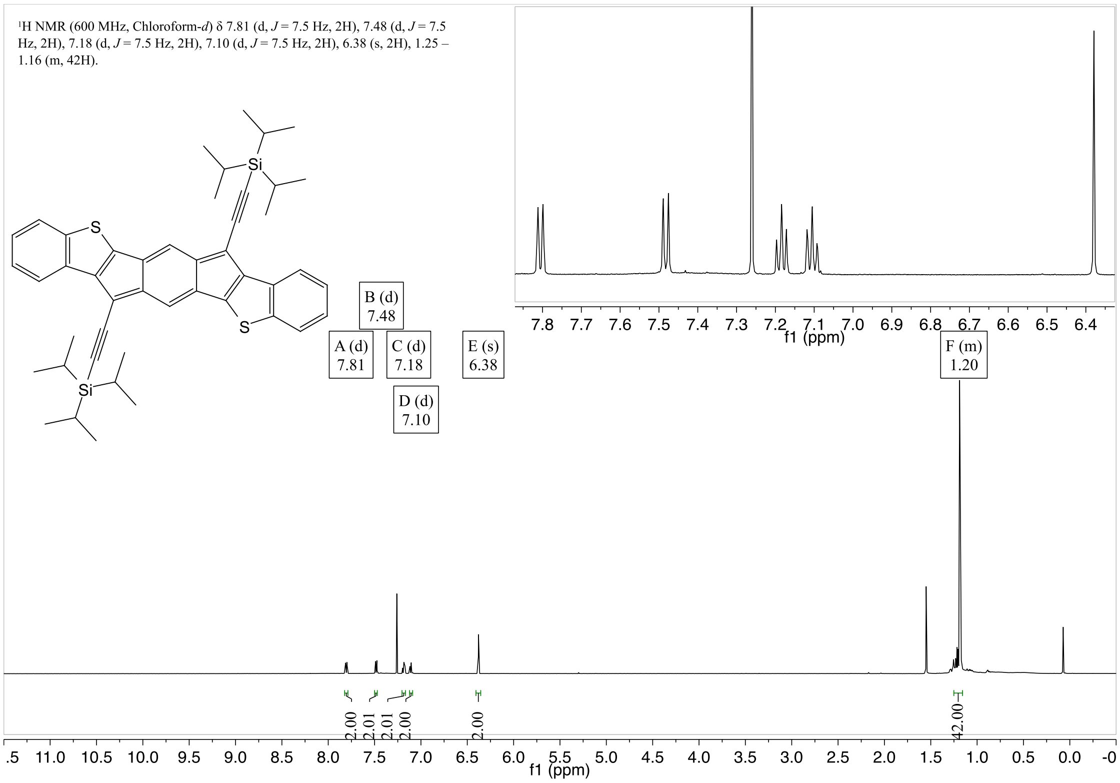

¹H NMR (600 MHz, Chloroform-*d*) δ 7.81 (d, *J* = 7.5 Hz, 2H), 7.48 (d, *J* = 7.5 Hz, 2H), 7.18 (d, *J* = 7.5 Hz, 2H), 7.10 (d, *J* = 7.5 Hz, 2H), 6.38 (s, 2H), 1.25 – 1.16 (m, 42H).
Si
S
S
Si
A (d) 7.81
B (d) 7.48
C (d) 7.18
D (d) 7.10
E (s) 6.38
F (m) 1.20
2.00
2.01
2.01
2.00
2.00
42.00
f1 (ppm)

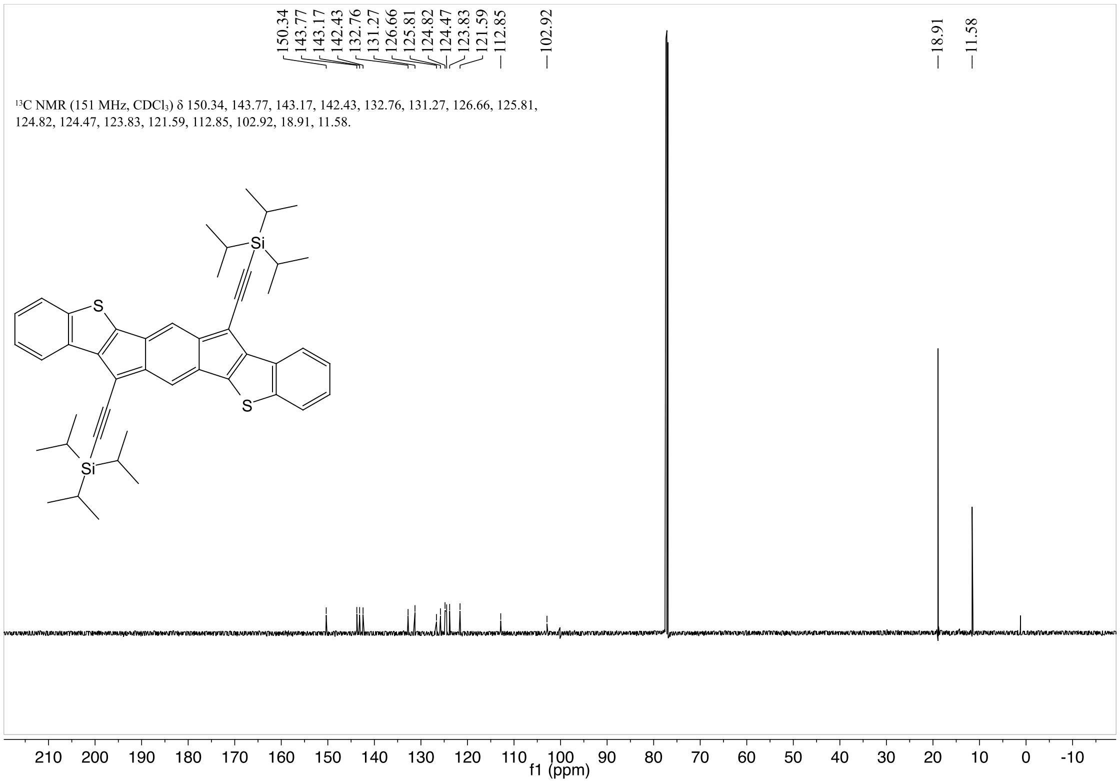

150.34
143.77
143.17
142.43
132.76
131.27
126.66
125.81
124.82
124.47
123.83
121.59
112.85
102.92
18.91
11.58
¹³C NMR (151 MHz, CDCl₃) δ 150.34, 143.77, 143.17, 142.43, 132.76, 131.27, 126.66, 125.81, 124.82, 124.47, 123.83, 121.59, 112.85, 102.92, 18.91, 11.58.
Si
S
S
Si
f1 (ppm)

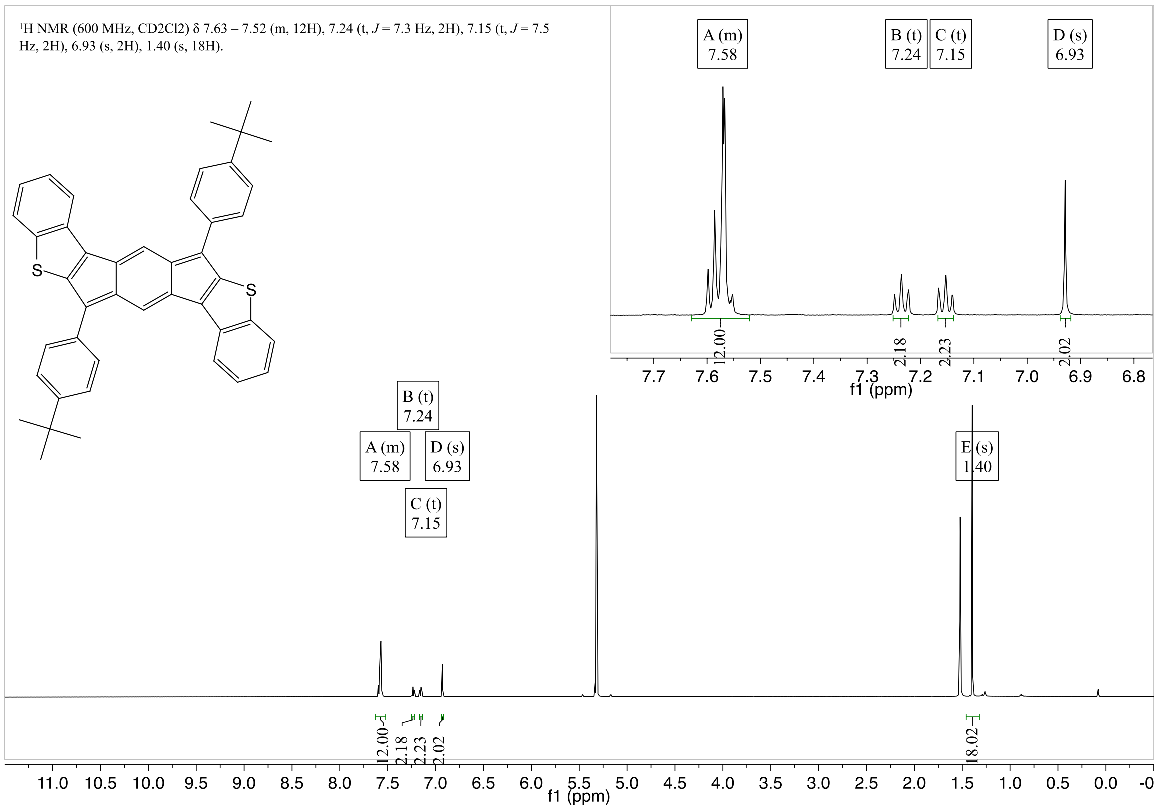
1H NMR (600 MHz, CD2Cl2) δ 7.63 – 7.52 (m, 12H), 7.24 (t, J = 7.3 Hz, 2H), 7.15 (t, J = 7.5 Hz, 2H), 6.93 (s, 2H), 1.40 (s, 18H).
S
S
A (m) 7.58
B (t) 7.24
C (t) 7.15
D (s) 6.93
E (s) 1.40
12.00
2.18
2.23
2.02
18.02
f1 (ppm)

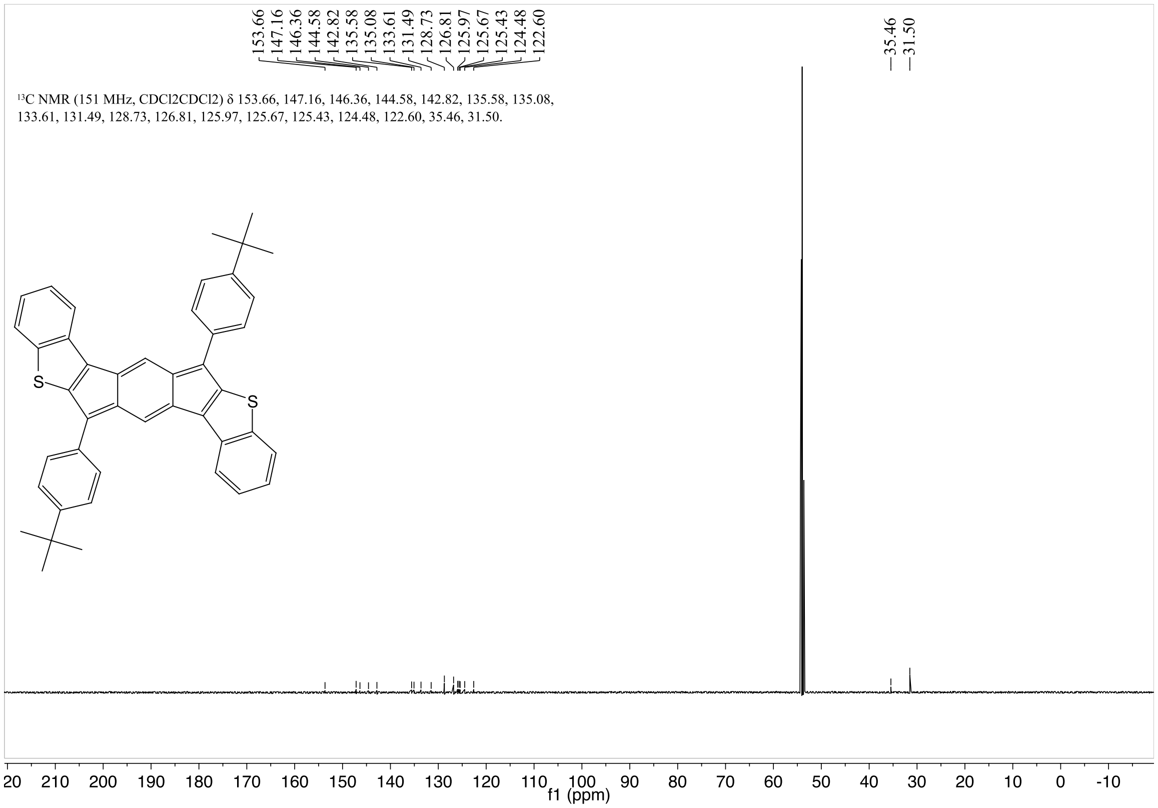
153.66 147.16 146.36 144.58 142.82 135.58 135.08 133.61 131.49 128.73 126.81 125.97 125.67 125.43 124.48 122.60
35.46 31.50
13C NMR (151 MHz, CDCl2CDCl2) δ 153.66, 147.16, 146.36, 144.58, 142.82, 135.58, 135.08, 133.61, 131.49, 128.73, 126.81, 125.97, 125.67, 125.43, 124.48, 122.60, 35.46, 31.50.
S
S
f1 (ppm)

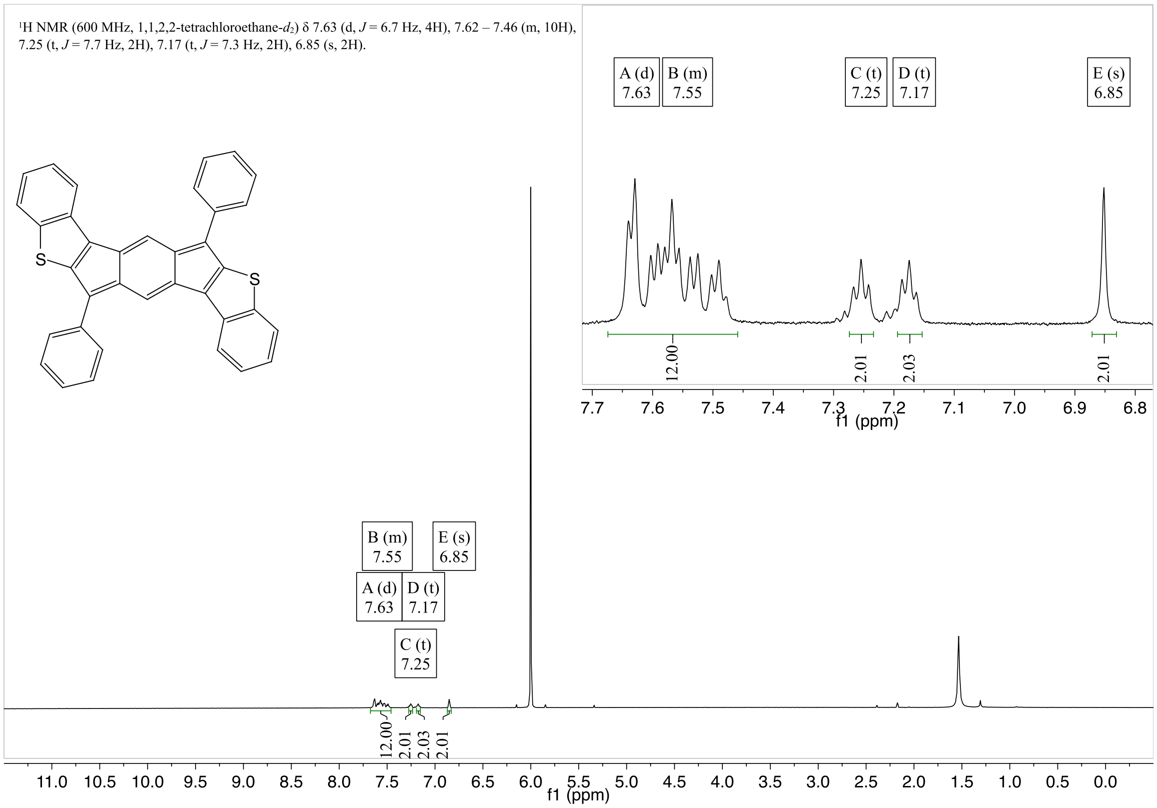
¹H NMR (600 MHz, 1,1,2,2-tetrachloroethane-d_2) δ 7.63 (d, J = 6.7 Hz, 4H), 7.62 – 7.46 (m, 10H), 7.25 (t, J = 7.7 Hz, 2H), 7.17 (t, J = 7.3 Hz, 2H), 6.85 (s, 2H).
S
S
A (d) 7.63
B (m) 7.55
C (t) 7.25
D (t) 7.17
E (s) 6.85
12.00
2.01
2.03
2.01
f1 (ppm)

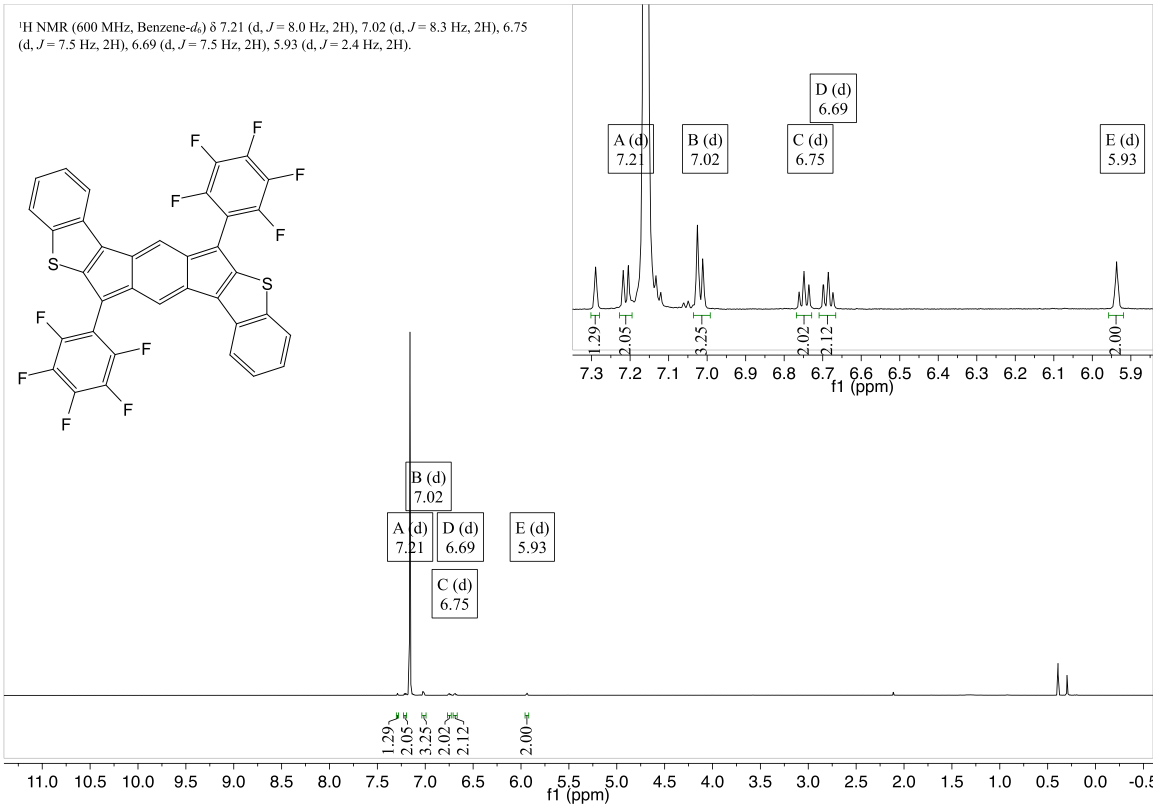
¹H NMR (600 MHz, Benzene-d_6) δ 7.21 (d, J = 8.0 Hz, 2H), 7.02 (d, J = 8.3 Hz, 2H), 6.75 (d, J = 7.5 Hz, 2H), 6.69 (d, J = 7.5 Hz, 2H), 5.93 (d, J = 2.4 Hz, 2H).
F
S
S
A (d) 7.21
B (d) 7.02
C (d) 6.75
D (d) 6.69
E (d) 5.93
1.29
2.05
3.25
2.02
2.12
2.00
f1 (ppm)

^{19}F NMR (282 MHz, THF-d_8) δ -135.10 (d, J = 22.5 Hz), -153.12 (t, J = 20.8 Hz), -161.03 (t, J = 18.6 Hz).

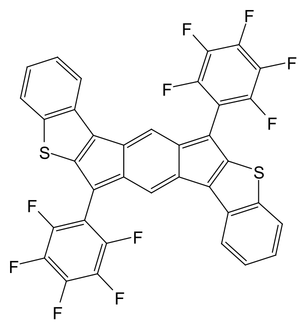

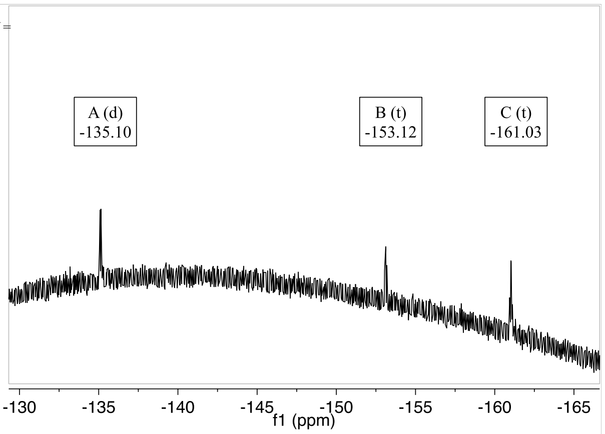

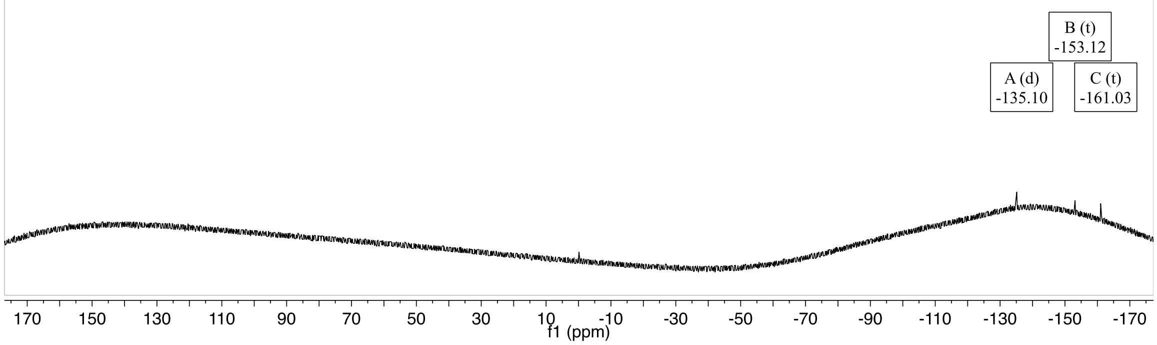

^{1}H NMR (500 MHz, 1,1,2,2-tetrachloroethane-d_2) δ 8.00 (s, 4H), 7.97 (s, 2H), 7.58 (d, J = 8.0 Hz, 2H), 7.39 (d, J = 8.0 Hz, 2H), 7.26 (t, J = 7.2 Hz, 2H), 7.21 (d, J = 7.3 Hz, 2H), 6.54 (s, 2H).

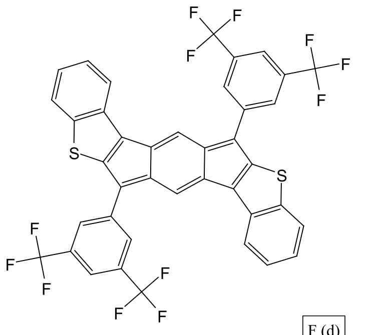

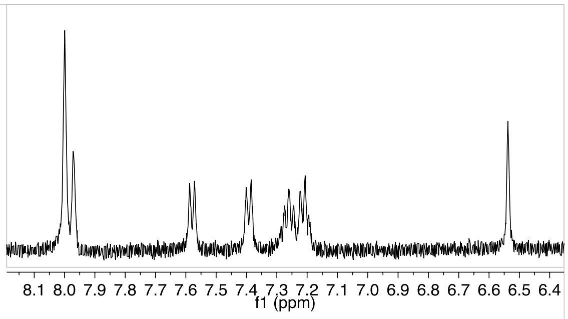

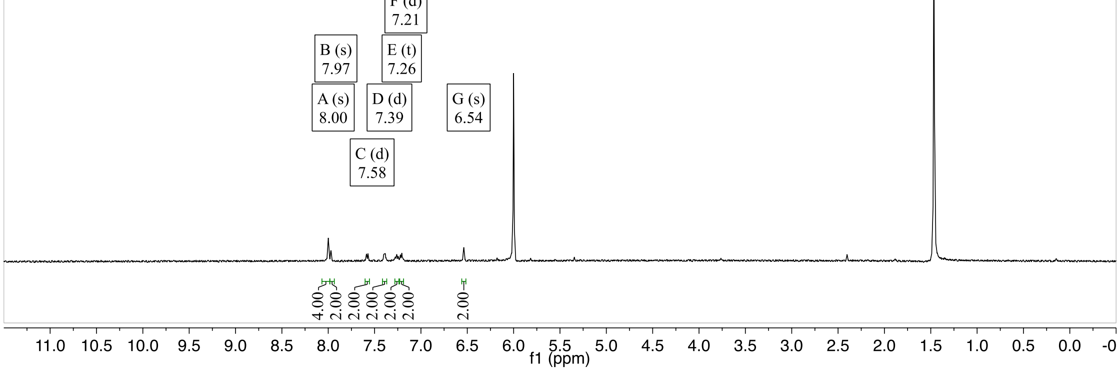

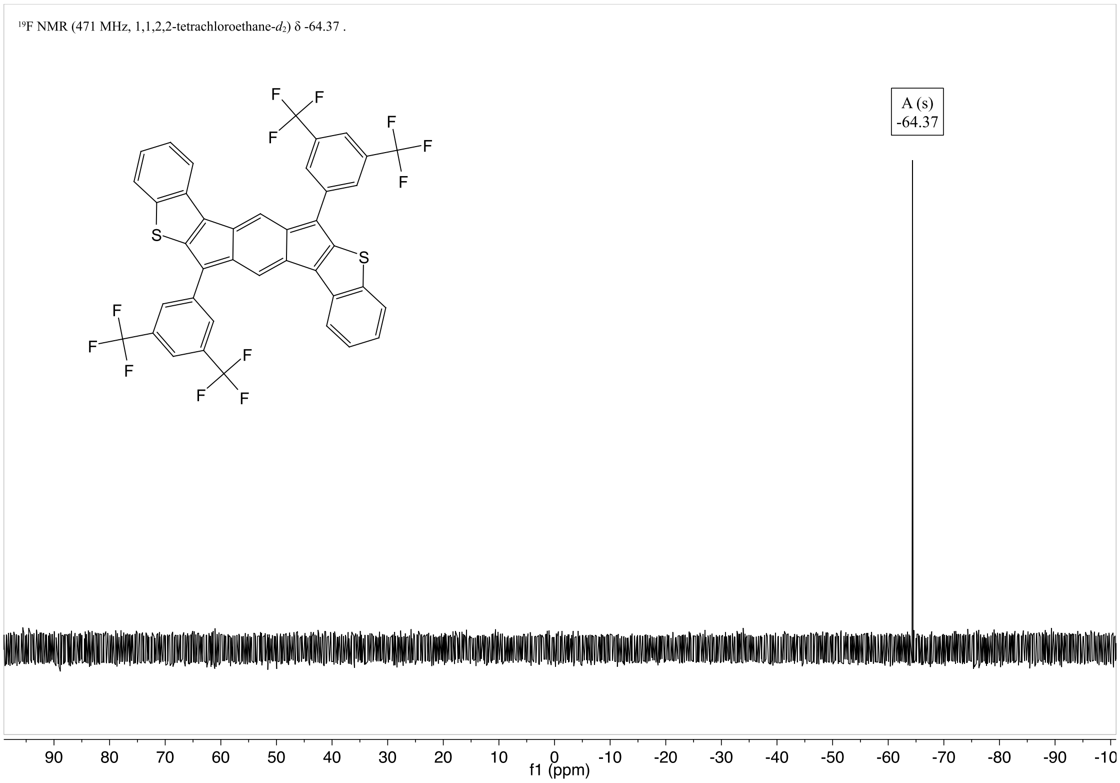

19F NMR (471 MHz, 1,1,2,2-tetrachloroethane-d2) δ -64.37 .
A (s)
-64.37
f1 (ppm)

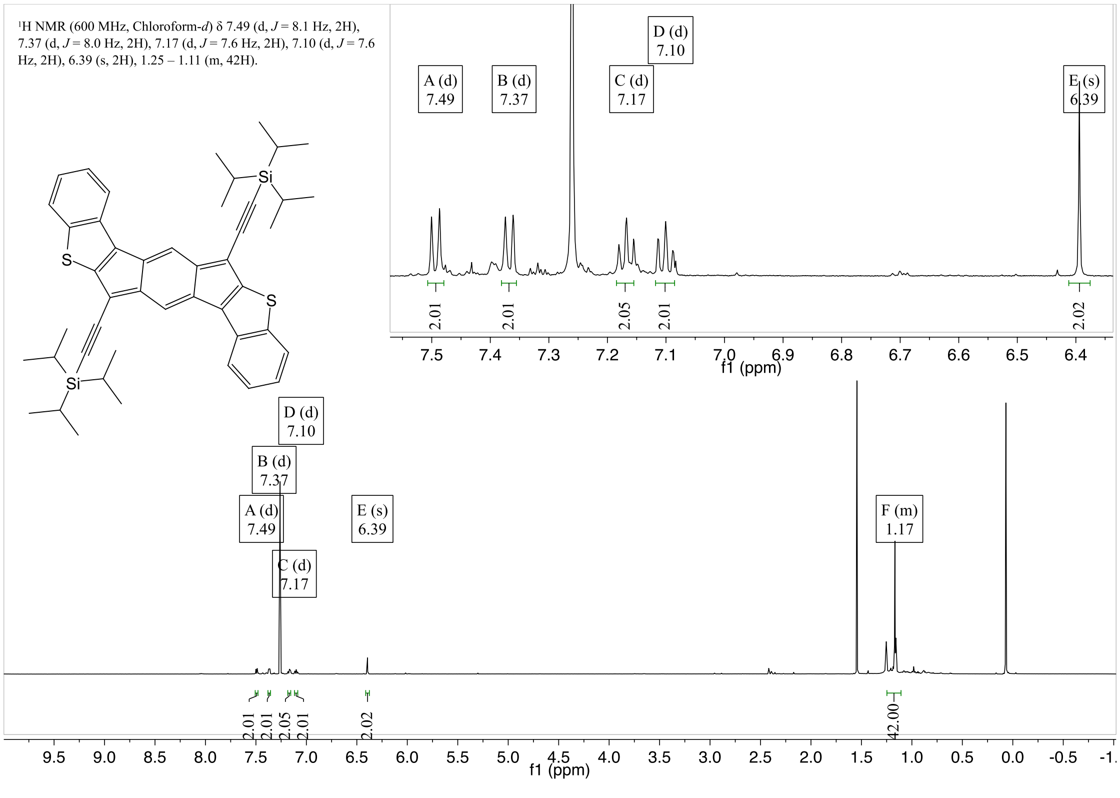

1H NMR (600 MHz, Chloroform-d) δ 7.49 (d, J = 8.1 Hz, 2H), 7.37 (d, J = 8.0 Hz, 2H), 7.17 (d, J = 7.6 Hz, 2H), 7.10 (d, J = 7.6 Hz, 2H), 6.39 (s, 2H), 1.25 – 1.11 (m, 42H).
A (d)
7.49
B (d)
7.37
C (d)
7.17
D (d)
7.10
E (s)
6.39
F (m)
1.17
2.01
2.01
2.05
2.01
2.02
42.00
f1 (ppm)
